# Targeting *Bacillus anthracis* toxicity with a genetically selected inhibitor of the PA/CMG2 protein-protein interaction

**DOI:** 10.1038/s41598-017-03253-3

**Published:** 2017-06-08

**Authors:** Abigail L. Male, Fedor Forafonov, Francesco Cuda, Gong Zhang, Siqi Zheng, Petra C. F. Oyston, Peng R. Chen, E. Diane Williamson, Ali Tavassoli

**Affiliations:** 1 Chemistry, University of Southampton, Southampton, SO17 1BJ United Kingdom; 2 Peking-Tsinghua Center for Life Sciences, Academy for Advanced Interdisciplinary Studies, Peking University, Beijing, China; 3 Beijing National Laboratory for Molecular Sciences, Synthetic and Functional Biomolecules Center, College of Chemistry and Molecular Engineering, Peking University, Beijing, China; 4 Defence Science and Technology Laboratory, Porton Down, Salisbury, UK; 5 Institute for Life Sciences, University of Southampton, Southampton, United Kingdom

## Abstract

The protein-protein interaction between the human CMG2 receptor and the *Bacillus anthracis* protective antigen (PA) is essential for the transport of anthrax lethal and edema toxins into human cells. We used a genetically encoded high throughput screening platform to screen a SICLOPPS library of 3.2 million cyclic hexapeptides for inhibitors of this protein-protein interaction. Unusually, the top 3 hits all contained stop codons in the randomized region of the library, resulting in linear rather than cyclic peptides. These peptides disrupted the targeted interaction *in vitro*; two act by binding to CMG2 while one binds PA. The efficacy of the most potent CMG2-binding inhibitor was improved through the incorporation of non-natural phenylalanine analogues. Cell based assays demonstrated that the optimized inhibitor protects macrophages from the toxicity of lethal factor.

## Introduction

Anthrax is caused by *Bacillus anthracis* (*B. anthracis*), a spore-forming encapsulated Gram positive bacterium^1, 2^. The disease is classified depending on the route of exposure; the most common form in humans is cutaneous anthrax, associated with skin lesions and is manageable with antibiotics. Gastrointestinal anthrax causes a much more serious systemic disease but primarily affects livestock that have ingested the bacterial spores. The third form of the disease is pulmonary anthrax, which results from inhalation of airborne spores and is potentially fatal. Pulmonary anthrax is asymptomatic for several weeks as lung macrophages and dendritic cells engulf and kill most inhaled spores^3^. A fraction of the spores survive within the alveolar macrophages and are transported to tracheobronchial and mediastinal lymph nodes, where they germinate, giving rise to mild non-specific symptoms (fever, aches, cough). The disease progresses rapidly from these flu-like symptoms as bacteria reach high levels in the circulation, causing fulminant disease characterised by respiratory impairment, shock and widespread haemorrhage. Antibiotics are without therapeutic benefit from this point onwards due to the accumulation of the bacterial toxins, and death usually occurs within 24 hours^4^.

The basis for anthrax virulence is well understood at the molecular level. The genes encoding the secreted binary toxins of *B. anthracis*, named lethal toxin (LT) and edema toxin (ET), reside on a self-replicating 184-kb plasmid termed pXO1. Anthrax toxin consists of three distinct proteins named protective antigen (PA), lethal factor (LF), and edema factor (EF)^5^. PA is an 83 kDa protein that binds to one of two cell surface receptors, then undergoes furin protease-mediated cleavage to yield a 63 kDa fragment. This cleavage is essential for toxin action and PA harbouring mutations in the furin cleavage site is completely non-toxic and devoid of pathogenic effects *in vivo* ^6^. EF is an 89 kDa calcium and calmodulin-dependent adenylate cyclase that causes a dramatic increase in cytoplasmic cAMP levels, impairing neutrophil function and affecting water homeostasis, leading to edema. LF is a 90 kDa zinc-dependent metalloproteinase that specifically cleaves and inactivates mitogen activated protein kinase kinases (MAPKK), which blocks several signal transduction pathways, leading to apoptosis and lysis within a few hours. The furin-cleaved PA binds one of its two target cell receptors; tumour endothelial marker 8 (TEM8), or capillary morphogenesis gene 2 (CMG2). Once bound to the cell, PA assembles into a ring shaped heptamer that forms membrane spanning pores^7^, acting as a protein translocator, escorting three molecules of LF or EF from the extracellular environment into the cytoplasm^8^. Association of PA monomers occurs spontaneously, and LF and EF can only bind to the oligomeric forms of PA^9, 10^. A series of elegant experiments with mice lacking TEM8, CMG2 or both, have demonstrated that the main receptor for anthrax toxicity is CMG2^11^. This may be due to the higher affinity of PA for CMG2 (170 pM) versus TEM8 (1.1 µM)^12^, or the fact that CMG2 is preferentially expressed in cells important for infection and/or toxin-induced death^13^.

Previous attempts at targeting anthrax toxicity have included inhibition of proteolytic activation of PA^14^, inhibiting LF enzymatic activity^15–17^, and inhibiting EF enzymatic activity^18^. In addition to this, cisplatin was identified in a high-throughput screen as binding CMG2^19^, and phage display has been used to identify a 12-residue peptide (AWPLSQLDHSYN) that binds to CMG2 and TEM8, with multiple copies of this peptide used to assemble polyvalent liposomes that inhibited anthrax toxicity^20^. Here we describe the identification and *in vitro* validation of a linear pentapeptide that binds CMG2 and inhibits the protein-protein interaction (PPI) with PA.

## Results

### Construction of a PA/CMG2 RTHS and SICLOPPS screening

**Figure 1:**
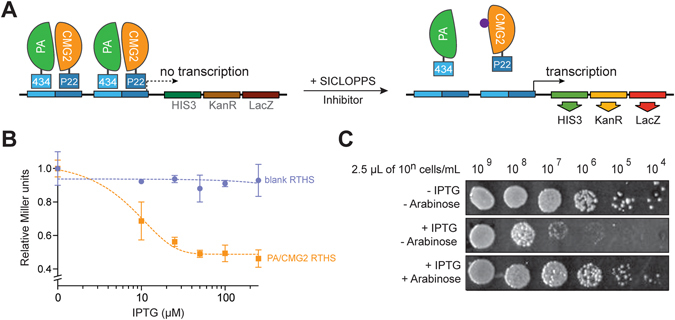
PA/CMG2 reverse two-hybrid system. (**A**) PA_488–735_ is expressed as a fusion with the 434 bacteriophage DNA binding protein and CMG2_38–218_ is expressed as a fusion with a chimeric P22 DNA binding protein. These proteins associate to form a functional repressor that prevents transcription of the 3 reporter genes (HIS3, KanR and LacZ) downstream of the operator sites, leading to cell death on selective media. In the presence of an inhibitor of the PA/CMG2 interaction from the SICLOPPS library, the repressor complex is disrupted, enabling expression of the reporter genes and survival of the host on selective media. (**B**) ONPG assay of the PA/CMG2 RTHS shows a loss of lacZ expression in response to increased doses of IPTG, with no such effect in the blank strain expression the repressor domains alone. Data represented as mean ± SEM. (**C**) drop spotting 10-fold serial dilutions (2.5 µL of 10^n^ cells/mL) of the PA/CMG2 RTHS with a potential SCILOPPS inhibitor. In the absence of IPTG and arabinose, full growth is observed, whereas in the presence of 50 µM IPTG growth of the RTHS is repressed by ~4 spots. In the presence of 6.5 µM arabinose (inducing SICLOPPS) and 50 µM IPTG growth of the RTHS is restored, likely via disruption of the PA/CMG2 PPI.

We employed a genetically encoded, high-throughput screening platform that combines a bacterial reverse two-hybrid system (RTHS)^21, 22^ to screen a library of 3.2 million cyclic peptides generated by split-intein circular ligation of peptides and proteins (SICLOPPS)^23–25^. The RTHS links the survival and growth of engineered *E. coli* to the interaction of the targeted PPI via three reporter genes (Fig. 1A). PA is composed of four distinct domains, termed domain I–IV (Supplemental Figure 1A)^26, 27^; three isopropyl β-D-1-thiogalactopyranoside (IPTG) induced plasmids each encoding full length PA, domain II to domain IV of PA_259–735_, or domain III and IV of PA_488–735_ as an N-terminal fusion with the 434 repressor, and the extracellular portion of CMG2_38–218_ as an N-terminal fusion with a chimeric P22 repressor were constructed. These plasmids were integrated onto the chromosome of the *E. coli* heterodimeric RTHS strain as previously detailed^28^. Association of PA with CMG2 will enable the formation of a functional functional 434/P22 repressor that binds to operator sites engineered onto the chromosome of *E. coli*, preventing expression of 3 reporter genes (HIS3, the yeast auxotroph of the HISB histidine biosynthesis gene which has been deleted form the reporter strain; KanR, encoding kanamycin resistance; and LacZ, encoding β-galactosidase). Thus interaction of the targeted proteins will lead to cell death on selective media. The resulting PA/CMG2 RTHS were assessed for functional repression upon addition of IPTG (causing expression and interaction of PA/CMG2) by drop-spotting. Only the domain III–IV PA_488–735_ RTHS showed a reduction in growth in response to IPTG by drop-spotting (Supplemental Figure 1B). This RTHS was further characterised by *o*-nitrophenyl-β-galactoside (ONPG) assay and additional drop-spotting. An IPTG-dependent reduction in β-galactosidase activity was observed, indicating the formation of a functional repressor with 25 µM IPTG (Fig. 1B). The addition of 25 µM IPTG was sufficient to reduce the survival and growth of the PA/CMG2 RTHS on selective media lacking histidine and containing kanamycin (Fig. 1C, top row versus second row), confirming the formation of a functional repressor.

An arabinose-induced SICLOPPS library encoding cyclic hexa-peptides with a cysteine in position 1 as required for intein splicing (Supplemental Figure 2A), followed by five random amino acids (CX_5_) was constructed as previously detailed^24, 29^ and transformed into the PA/CMG2 RTHS. Peptides disrupting the PA/CMG2 PPI would also disrupt the 434/P22 repressor and enable expression of the reporter genes and host survival and growth on selective media (Fig. 1A). The transformation efficiency of the CX_5_ library into electro competent PA/CMG2 RTHS cells was measured as 3 × 10^7^, ensuring ten-fold coverage of each member of the library in our screen. 480 colonies survived and grew on selective media supplemented with IPTG and arabinose. These colonies were isolated and assessed for retention of phenotype by drop spotting (Fig. 1C); IPTG-dependent formation of the functional repressor was assessed by drop-spotting onto selective media with and without IPTG, and the ability of the SICLOPPS-derived cyclic peptide to disrupt the PA/CMG2 PPI was assessed by drop-spotting onto selective media containing both IPTG and arabinose (Fig. 1C). The relative potency of each cyclic peptide may be indirectly assessed through the number of spots of growth on the IPTG + arabinose plate, with more potent inhibitors enabling further growth. The 27 cyclic peptides that restored the growth of the PA/CMG2 RTHS by two or more spots than the IPTG alone plate (~100-fold improvement in survival) were taken forward for secondary screening. The SICLOPPS plasmid from each of these 27 colonies was isolated and re-transformed into the PA/CMG2 RTHS for re-confirmation of phenotype. These plasmids were also transformed into another RTHS monitoring for the p6/UEV PPI^30^; this RTHS is identical to the PA/CMG2 RTHS except for the targeted PPI. Cyclic peptides that enable survival by targeting components of the RTHS other than the PA/CMG2 PPI (e.g. inhibiting the interaction of the repressor domains with DNA) would also be active in the p6/UEV RTHS. Any isolated SICLOPPS plasmids that also enabled survival and growth of the p6/UEV RTHS were therefore discarded. The remaining nine SICLOPPS plasmids were ranked for activity by drop spotting, and sequenced to reveal the identity of the cyclic peptide encoded (Supplemental Table 1). Surprisingly, 7 of the 9 sequences, including all of the top ranking hits, contained a stop codon. This produces a truncated SICLOPPS protein that displays a short peptide aptamer from the C-intein (Supplemental Figure 2B and C), instead of a cyclic peptide. SICLOPPS libraries are constructed with a degenerate oligonucleotide that uses an NNS codon set (N = any base, S = C or G) for each of the 5 random amino acid positions, resulting in only the TAG stop codons being present in the screened library. It should be noted that this is highly unusual; there are no previous reports of this occurrence, and we very rarely isolate sequences containing a stop codon in SICLOPPS screens^31, 32^. Interestingly, 2 of the top 9 hits were linear heptamers, generated by deletion of the first T in the SICLOPPS N-intein; this causes a frameshift, changing TGC TTA AGT (the sequence following the last randomized amino acid, encoding C, L, and S) to GCT TAA GT (encoding A and Stop). The observed prevalence of stop codons in the most potent hits from our SICLOPPS library strongly suggests that the cyclic hexapeptide scaffold is not optimal for disrupting the PA/CMG2 PPI, and/or the optimal pocket for disrupting this PPI does not accept residues displayed by this scaffold. The stop codon results in only linear aptamers being presented, either through incorporation of an amber stop codon, or selective pressure leading to a point deletion, which also results in a stop codon. It is also worth noting that beyond the prevalence of stop codons, the only other consensus in the isolated sequences is between 2 of the 3 most potent hits, which differ by only 1 amino acid (CLRFT and CLRPT). There is little consensus in the sequence or any motif(s) present amongst the other, less potent hits.

### *In vitro* quantification of the PA/CMG2 PPI inhibitors

**Figure 2:**
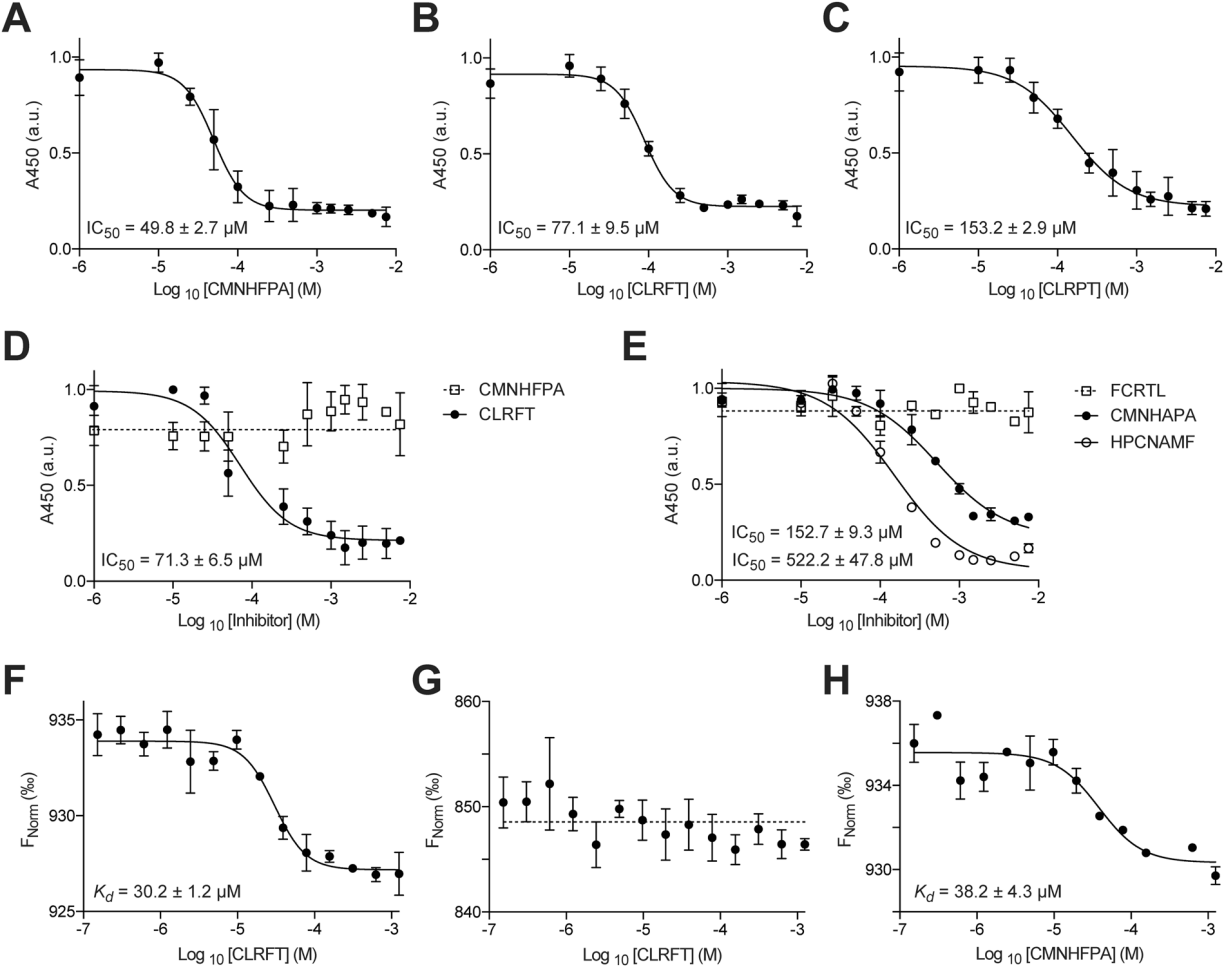
*In vitro* analysis of the selected PA/CMG2 inhibitors by ELISA and MST. (**A**) CMNHFPA disrupts the interaction of His_6_-PA_488–735_ and GST-CMG2_38–218_ with an IC_50_ of 49.8 ± 2.7 µM. (**B**) CLRFT disrupts the interaction of His_6_-PA_488–735_ and GST-CMG2_38–218_ with an IC_50_ of 77.1 ± 9.5 µM. (**C**) CLRPT disrupts the interaction of His_6_-PA_488–735_ and GST-CMG2_38–218_ with an IC_50_ of 153.2 ± 2.9 µM. (**D**) CLRFT disrupts the interaction of His_6_-PA_596–735_ and GST-CMG_238–218_ with an IC_50_ of 71.3 ± 6.5 µM, whereas CMNHFPA is not active in the absence of domain III of PA. (**E**) FCRTL (scrambled CLRFT) does not inhibit the interaction of His_6_-PA_488–735_ and GST-CMG2_38–218_, whereas HPCNAMF (scramble of CMNHFPA) inhibits this interaction with an IC_50_ of 152.7 ± 9.3 µM. CMNHAPA inhibits this interaction IC_50_ of 522.2 ± 47.8 µM. **(F)** CLRFT binds to CMG_238–218_ with a K_d_ of 30.2 ± 1.2 µM. **(G)** CLRFT does not bind PA_488–735_. **(H)** CMNHFPA binds to PA_488–735_ with a K_d_ of 38.2 ± 4.3 µM. All data represented as mean ± SEM, n = 3.

The 3 top ranking compounds isolated from our screen were synthesized by Fmoc solid-phase peptide synthesis and assessed for the ability to disrupt the PA/CMG2 PPI *in vitro*. We developed a sandwich ELISA to monitor the interaction between His_6_-PA_488–735_ (domains III and IV) and GST-CMG2_38–218_ and used this assay to assess the activity of our top 3 inhibitors. The most potent compound was CMNHFPA with an IC_50_ of 49.8 ± 2.7 µM, followed by CLRFT with an IC_50_ of 77.1 ± 9.5 µM and CLRPT with IC_50_ of 153.2 ± 2.9 µM (Fig. 2A,B and C). Given the relatively weak activity of CLRPT, it was not carried forward for further assessment.

We repeated the above ELISA using domain IV of PA (His_6_-PA_596–735_) and GST-CMG2_38–218_; CLRFT showed a similar level of activity as before with an IC_50_ of 71.3 ± 6.5 µM, whereas CMNHFPA lost all activity (Fig. 2D). Given that CMNHFPA is inactive in the absence of domain III of PA, one may hypothesise that this cyclic peptide functions by binding to domain III of PA; however, structural data indicate that domain III of PA is not in direct contact with CMG2 (Supplemental Fig. 1A)^26^. Considering these two points together, one explanation may be that CMNHFPA inhibits the of the PA/CMG2 PPI by binding to an allosteric site on domain III of PA. We next synthesized scrambled analogues of our top 2 inhibitors as negative controls, to assess the sequence dependence of activity. FCRTL (scramble of CLRFT) was found to be inactive in the PA/CMG2 ELISA, whereas HPCNAMF (scramble of CMNHFPA) inhibited the PA/CMG2 PPI with an IC_50_ of 152.7 ± 9.3 µM, a 3-fold loss of activity over the selected peptide. Given the retention of some activity of the scramble peptide, we further assessed the sequence specificity of CMNHFPA by replacing phenylalanine with alanine; the resulting molecule (CMNHAPA) disrupted the PA/CMG2 PPI with an IC_50_ of 522.2 ± 47.8 µM, a 10-fold loss of activity from the parent molecule. The retention of activity in these control molecules may result from part of the active motif of the parent molecule being retained in the scramble molecule (or reconstituted through folding of the peptide); alternatively, the parent molecule may be a false positive.

The protein target of CLRFT was identified, and the binding affinity quantified, using microscale thermophoresis (MST). CLRFT bound CMG2 with a *K*_*d*_ of 30.2 ± 1.2 µM (Fig. 2F), while no binding was measured to PA (Fig. 2G). Our ELISA data indicated that CMNHFPA bound to PA (Fig. 2A and D), and we measured a *K*_*d*_ of 38.2 ± 4.3 µM (Fig. 2F) for this interaction by MST.

Although both of the 2 most potent PA/CMG2 inhibitors identified from the CX_5_ library were linear, the *in vitro* data shows that one acts by binding PA, while the other acts by binding CMG2. CMNHFPA is more potent inhibitor of the PA/CMG2 PPI than CLRFT (Fig. 2A and B), but both peptides bind their respective targets with similar affinity (Fig. 2F and H). Although both these molecules could form the starting point for hit optimization, the binding of CLRFT to CMG2 (rather than PA) may be seen as an advantage, especially with respect to the reduced potential for resistance through mutation.

### Improving the affinity of CLRFT for CMG2 through non-natural analogues

**Figure 3:**
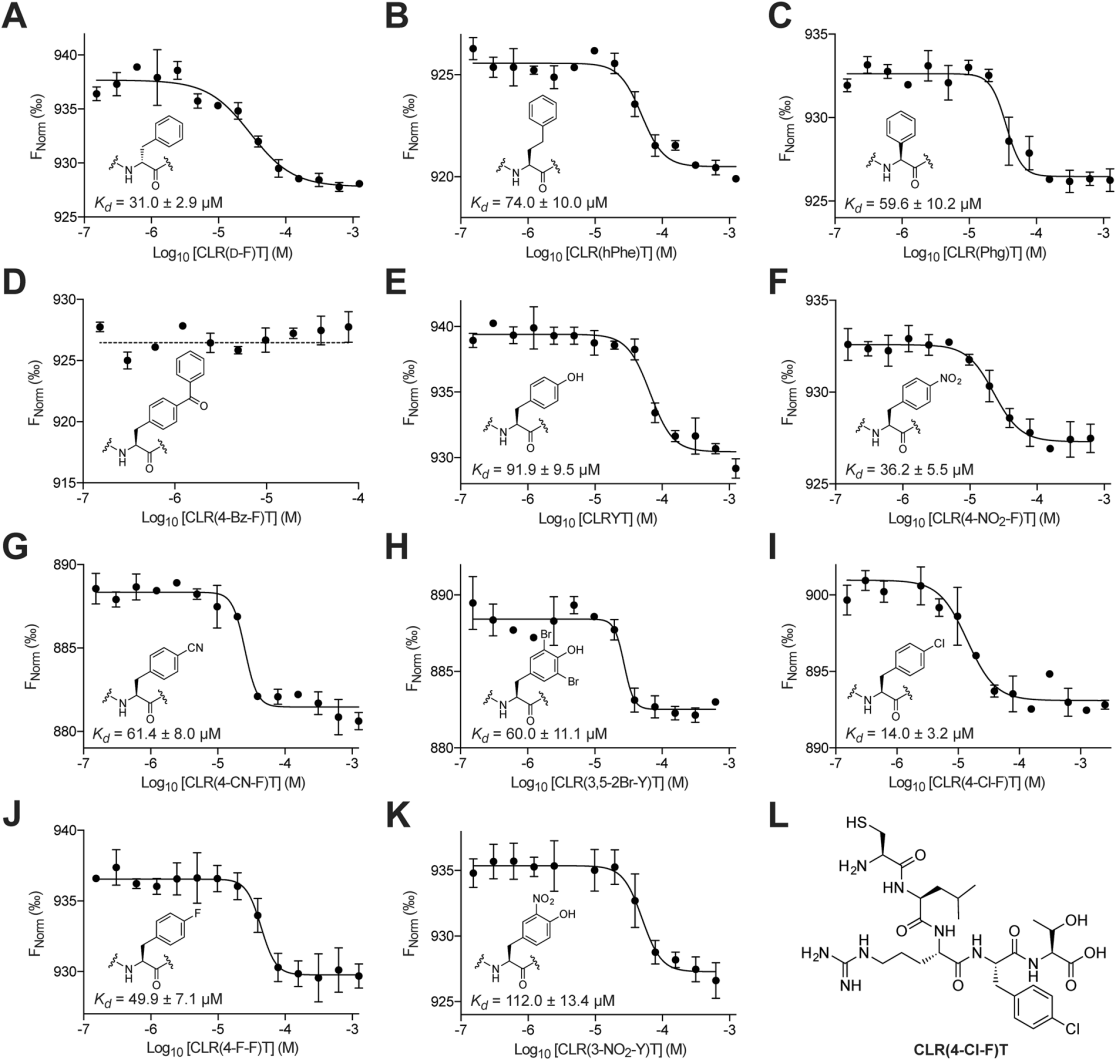
MST analysis of the affinity of CLRFT analogues containing the illustrated non-natural phenylalanine derivatives in place of phenylalanine. (**A**) The D-phenylalanine analogue binds to CMG2_38–218_ with a *K*_*d*_ of 31.0 ± 2.9 µM. (**B**) The homophenylalanine analogue binds to CMG2_38–218_ with a *K*_*d*_ of 74.0 ± 10.0 µM. (**C**) The phenylglycine analogue binds to CMG2_38–218_ with a *K*_*d*_ of 59.6 ± 10.2 µM. (**D**) The 4-benzoyl-phenylalanine analogue does not bind to CMG2_38–218_. (**E**) The tyrosine analogue binds to CMG2_38–218_ with a *K*_*d*_ of 91.9 ± 9.5 µM. (**F**) The 4-nitro-phenylalanine analogue binds to CMG2_38–218_ with a *K*_*d*_ of 36.2 ± 5.5 µM. (**G**) The 4-cyano-phenylalanine analogue binds to CMG2_38–218_ with a *K*_*d*_ of 61.4 ± 8.0 µM. (**H**) The 3,5-dibromo-tyrosine analogue binds to CMG2_38–218_ with a *K*_*d*_ of 60.0 ± 11.1 µM. (**I**) The 4-chlorophenylalanine analogue binds to CMG2_38–218_ with a *K*_*d*_ of 14.0 ± 3.2 µM. (**J**) The 4-fluoro-phenylalanine analogue binds to CMG2_38–218_ with a *K*_*d*_ of 49.9 ± 7.1 µM. (**K**) The 3-nitro-tyrosine analogue binds to CMG2_38–218_ with a *K*_*d*_ of 112.0 ± 13.4 µM. (**L**) The structure of the most potent analogue CLR(4-Cl-F)T. All data represented as mean ± SEM, n = 3.

We synthesized several analogues of CLRFT containing non-natural phenylalanine derivatives, with the aim of probing binding efficacy and improving the potency of this molecule. Phenylalanine was chosen as the residue due to the large number of commercially available non-natural analogues, as well as the loss of activity observed when this amino acid was replaced with a proline (in the third most potent hit identified, CLRPT, Fig. 2A and C), although it should be noted that this loss of activity may be due to the effect of proline on peptide backbone conformation. The analogues were synthesized and their binding affinity for CMG2 measured by MST (Fig. 3). We initially probed the effect of stereochemistry of this residue by incorporating D-phenylalanine in this position, however, we saw little effect on binding (*K*_*d*_ = 31.0 ± 2.9 μM, Fig. 3A). We next probed the length of the binding cavity; a 2.5-fold reduction in *K*_*d*_ was observed when using homophenylalanine (Fig. 3B), and a 2-fold reduction in *K*_*d*_ was observed when using phenylglycine (Fig. 3C). In line with this data, complete loss of binding was observed when 4-benzoyl-phenylalanine was used (Fig. 3D). We next probed the electronic requirements of the binding pocket by using a variety of electron donating and withdrawing substituents (Fig. 3E–K, however, we observed little correlation between this and binding affinity. For example, using tyrosine caused a 3-fold reduction in the binding affinity (*K*_*d*_ = 91.9 ± 9.5 μM, Fig. 3E), while using 4-nitrophenylalanine had little effect (*K*_*d*_ = 36.2 ± 5.5 μM, Fig. 3F, yet the weaker electron-withdrawing 4-cyanophenylalanine reduced the binding affinity by 2-fold (*K*_*d*_ = 61.4 ± 8.0 μM, Fig. 3G). Of the compounds synthesized, only the 4-chlorophenylalanine derivative (Fig. 3L)showed an improvement in the binding, with a 2-fold increase in its affinity for CMG2 (*K*_*d*_ = 14.0 ± 3.2 μM, Fig. 3I).

### Assessing the activity of CLR(4-Cl-F)T in cells

**Figure 4:**
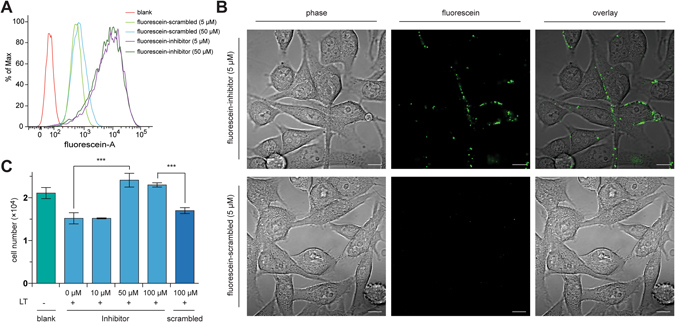
Cell-based analysis of CLR(4-Cl-F)T(inhibitor) and (4-Cl-F)CRTL(scrambled) activity. (**A**) Fluorescence-activated cell sorting shows that fluorescein-tagged CLR(4-Cl-F)T (at 5 or 50 µM) binds to BHK-21 cells, whereas the fluorescence from BHK-21 cells incubated with the fluorescein-tagged scrambled control, at 5 or 50 µM is weaker. (**B**) Confocal microscopy with the fluorescein-tagged CLR(4-Cl-F)T (5 µM) shows binding of the inhibitor to surface of BHK-21 cells, while binding by fluorescein-tagged scrambled control at 5 µM is not observed. Scale bar is 10 µm. (**C**) Toxin neutralization assay; CLR(4-Cl-F)T at 50 µM and above protects J774 cells from 0.1 mg/mL of lethal toxin, with a similar number of cells present as the no toxin control. No protective effect was observed from 100 µM (4-Cl-F)CRTL (scrambled control). ***p < 0.00; data represented as mean ± SEM.

We next sought to assess the activity of CLR(4-Cl-F)T (Fig. 3L), our most potent PA/CMG2 inhibitor, in cells. We initially determined binding of this molecule to its extracellular target by using a fluorescent derivative and its scrambled analogue (4-Cl-F)CRTL, generated by tagging cysteine with fluorescein-5-maleimide. The resulting molecules were incubated with BHK-21 cells, and binding to these cells was assessed by fluorescence-activated cell sorting. The data demonstrated that CLR(4-Cl-F)T binds to BHK-21 cells, with similar amount of fluorescence observed using 5 µM or 50 µM of this molecule (Fig. 4A), while 5 µM or 50 µM of the scrambled analogue showed weaker binding (Fig. 4A). Fluorescent microscopy was used to probe the cellular localization of these molecules. CLR(4-Cl-F)T was observed in the membrane of BHK-21 cells, while no binding was observed with the scrambled control at the same dose (Fig. 4B).

The ability of CLR(4-Cl-F)T to inhibit CMG2 for LT activity was probed using a toxin neutralization assay. J774 cells were treated with LT in the presence of increasing doses of CLR(4-Cl-F)T or its scrambled analogue, and the number of live cells determined after 18 hours. We observed a significant effect on cell viability from 50 µM of CLR(4-Cl-F)T, with the number of live cells equivalent to those not treated with LT (Fig. 4C). There was no such protection from LT for cells treated with 100 µM of the scrambled control (Fig. 4C).

## Discussion

A library of 3.2 million SICLOPPS peptides was screened for inhibitors of the PA/CMGS PPI. The 3 most potent inhibitors were found to contain a stop codon in the randomized region of the library, leading to the production of linear, rather than cyclic peptides. While the NNS codon used for the randomized region of SICLOPPS libraries eliminates 2 of the 3 stop codons, translation termination may still occur via a TAG codon in any of the 5 randomized positions. This is an unusual occurrence; we have not previously selected SICLOPPS hits containing stop codons, and this has not been reported by others^31^. Our findings suggest that members of the cyclic hexa-peptide library do not present their amino acid side chains in an orientation that enables binding to the pockets in the two targeted proteins, forcing the system to select linear peptides. Supporting our hypothesis of selective pressure for linear peptides are the hit peptides that contain a stop codon generated via a point mutation (resulting in a frame shift to give a stop codon). We are currently working to obtain structural information on the selected peptide/protein complexes, which will provide insight into the binding of each of these peptides and the reason for the prevalence of stop codons in their randomized region. Given the high binding affinity of the CMG2/PA interaction (*K*_*d*_ of 170 pM)^12^, the identified peptides are unlikely to dislodge PA bound to CMG2 by competing for the same binding interface. Given their *K*_*d*_ values, our inhibitors are much more likely to act by binding to an allosteric site on their target protein, and indirectly inhibiting the PPI.

The peptides reported above are not the first peptidic inhibitors of the PA/CMG2 PPI; phage display has been previously used to identify linear 12-mer peptides that bind to CMG2^20^. We are unable to compare the affinity of our hits with those previously reported, as the *in vitro* binding affinity of the previously reported peptides for their target proteins was not reported by the authors. However, there is no homology in the sequence of the previously reported peptides, and those reported here. This suggests that the peptides are binding to different regions for the target proteins, likely a result of the different methods used to identify the hits. Phage display selects for the most potent binding sequence, whereas our RTHS is a functional assay, seeking to identify the sequence that most effectively disrupts the interaction between the two given proteins. In addition, the linear pentamer reported here is substantially smaller than the previously reported 12-mers, and likely to be more readily translated to small molecule inhibitors of the PA/CMG2 PPI.

The similarity in the sequence of 2 of the top 3 hits, and the loss of potency caused by the change of phenylalanine to proline indicated the key role played by phenylalanine in binding to CMG2. A modest library of derivatives was therefore synthesized with non-natural phenylalanine analogues in order to improve binding affinity. The most potent analogue contained *para-*chlorophenylalanine, which bound CMG2 with a *K*_*d*_ of 14.0 ± 3.2 μM. This molecule was shown to be active in cells, protecting macrophages from lethal toxin at a dose of 50 µM. While this demonstrates the therapeutic potential of compounds derived from the molecules reported here, additional SAR studies, such as alanine scanning of the lead molecule are required for the design more potent inhibitors, as well as their derivatization into small molecule/non-peptidic compounds. Nonetheless, our screen has provided two sets of scaffolds that may be further developed as potential inhibitors of anthrax toxin entry into cells. While we have chosen to focus on development of the CMG2-binding compound identified here, similar optimization of the PA-binding molecule is also possible. Indeed, a possible strategy for treating anthrax infections may be with a cocktail of derivatives of both sets of molecules, blocking the PA/CMG2 interaction via both the human receptor, and the bacterial protein.

## Methods

### Construction of the PA/CMG2 RTHS

The RTHS used in this study was constructed as previously detailed for other RTHS^28^. Briefly, CMG2_38–218_ was cloned into the first multiple cloning site of pTHCP14^21^ via the XhoI and KpnI restriction endonuclease sites, while PA_488–735_ was cloned into the second multiple cloning site of this plasmid via the SalI and SacI restriction endonuclease sites. Formation of a functional repressor upon induction of the P22- CMG2_38–218_ and 434- PA_488–735_ fusion proteins was assessed by drop spotting and ONPG assays as previously detailed^28^, with the data shown in Fig. 1).

### SICLOPPS library construction

A SICLOPPS library encoding CXXXXX (X = any amino acid) was constructed as previously detailed^24^. Briefly, The C-terminal intein from pARCBD^23^ was amplified by PCR using the C + 5 forward primer, SICLOPPS reverse primer, and GoTaq (Promega), resulting in the incorporation of a region encoding the CXXXXX random sequence via the forward primer. The PCR product was purified and used as the template for a subsequent PCR reaction using SICLOPPS zipper primer, and SICLOPPS reverse primer (annealing temperature 65 °C and extension time 1 minute 15 seconds). The resulting PCR product and pARCBD plasmid were restriction digested with BglI and HindIII restriction endonucleases. The digested vector was gel purified to isolate the 3376 bp fragment corresponding to the plasmid backbone, and ligated with the restriction digested PCR product (1:3 insert to vector ratio) overnight at 4 °C. Salts were removed from the ligation mixture by dialysis on a nitrocellulose filter (13 mm, 0.025 μm, Millipore), for transformation into electrocompetent cells.

### SICLOPPS screening

The library ligation mixture was transformed into electrocompetent PA/CMG2 RTHS *E. coli* cells using standard protocols. The transformation mixture was recovered at 37 °C for 1 hour, 2 µL was removed for calculation of transformation efficiency by plating 10-fold serial dilutions of the recovery mixture on LB-agar media containing 30 µg/mL chloramphenicol and counting the number of surviving colonies at the highest dilution. The remaining 998 µL of the recovery mixture was plated onto M9 media-agar plates supplemented with 50 µg/ml ampicillin, 25 µg/mL spectinomycin, 30 µg/mL chloramphenicol, 50 µg/ml kanamycin, 5.0 mM 3-AT, 50 µM IPTG and 6.5 µM arabinose and incubated for 48–72 hours at 37 °C until individual colonies were visible. The 480 surviving colonies were picked and grown overnight in LB supplemented with 30 µg/mL chloramphenicol and drop-spotted onto minimal media plates as above with and without 50 µM IPTG and with and without 6.5 µM arabinose to check for retention of phenotype and rank activity.

The SICLOPPS plasmids from the 27 strains that retained their ability to enable survival on +IPTG/+ arabinose plates were isolated and transformed back into the PA/CMG2 RTHS, as well as the p6/UEV RTHS^30^; both RTHS are identical, except for the interacting protein pair. The resulting recovery mixtures were used to drop spot onto the same minimal media plates as above. Sequences that were active in both RTHS were discarded as non-specific (e.g. targeting a component of the RTHS other than the PPI). The SICLOPPS plasmids from the 9 strains showing selective inhibition of PA/CMG2 were sequenced to reveal the identity of the peptide inhibitors.

### Peptide synthesis

Peptides were synthesised by Fmoc solid-phase peptide synthesis on a 0.1 mmol scale using Wang resin preloaded with the first amino acid residue. Subsequent steps were performed at room temperature in a sintered funnel with agitation from a stream of argon. The amino acid coupling solution contained an Fmoc-protected amino acid (3 eq.), HOBt (3 eq.) and DIC (3 eq.) and was agitated with the resin in DMF for 1 h. The resin was washed with DMF, DCM and Et_2_O (20 mL of each) and successful coupling was checked using the Kaiser test, and the coupling step repeated if necessary. Fmoc deprotection was carried out by agitating the resin with 20% piperidine in DMF for 20 mins. The resin was washed as before, and successful deprotection checked using the Kaiser test prior to moving on. Upon deprotection of the final residue, the peptide chain was cleaved from the resin with 2 mL of TFA/TIS/H_2_O (95:2.5:2.5) for 2.5 h. The mixture was filtered through a sinter funnel, and the filtrate concentrated in vacuo. Peptides were precipitated from the remaining solution with cold Et_2_O added dropwise until a white precipitate formed. The solid was isolated, dried and dissolved in a H_2_O:MeCN mixture (1:1) prior to purification by preparative reverse-phase HPLC.

All HPLC was performed on a Waters 1525 HPLC system using linear gradients of solvents A (0.1% TFA/H_2_O) and B (0.1% TFA/MeCN). Peptides were purified by preparative HPLC with a Waters XSelect CSH C18 column (5.0 µm particle size, 19 × 250 mm), using a gradient from 95:5 to 50:50 A:B over 25 mins at 17 mL/min flow rate. Analytical HPLC was performed using a Waters Atlantis T3 C18 column (5.0 µm particle size, 4.6 × 100 mm) with the following method: 0–10 min: 95:5; 20–30 min: 40:60; 30–35 min: 95:5 A:B; at 1 mL/min flow rate. Please see supplemental data for the analytical spectra of each peptide.

### Sandwich ELISA

Glutathione S-transferase (GST)-CMG2_38–218_ and His_6_-PA_488–735_ were expressed and purified as previously detailed^12^. His_6_-PA_488–735_ (1,000 ng) was incubated in Ni^2+^-coated 96-well plates (Pierce) for 1 hour. The wells were washed with 3 × 200 µL of PBS with 0.05% Tween-20. GST-tagged CMG2_38–218_ (1,000 ng), incubated with various concentrations of inhibitor and 1 mM MgCl_2_, was added to each well and incubated for 1 hour. The wells were washed as before. Anti-GST (1 in 1000, MA4–004, Neomarkers) was added and incubated for 1 hour, after which the wells were washed as before. Anti-mouse-HRP (1 in 6000, NA931, GE Healthcare) was added and incubated for 1 hour, and the wells washed as before. 100 μL of 3,3′,5,5′-tetramethylbenzide (TMB)-Ultra ELISA solution (Fisher) was added to each well and incubated for 20 minutes. The signal was quenched with 1 M H_2_SO_4_ and the plate analysed at 450 nm. The procedure for the CMG2_38-218_ and PA_596–735_ ELISA was as above, except for the use of truncated PA.

### Microscale thermophoresis

MST experiments were performed on a Monolith NT.115 system (NanoTemper Technologies) using 100% LED and 40% IR-laser power. Laser on and off times were set at 30 seconds and 5 seconds, respectively. His_6_-CMG2_38–218_ and His_6_-PA_488–735_ were overexpressed, purified and labelled with NT647 (NanoTemper Technologies) and used at a final concentration of 80 nM. The inhibitors were dissolved in MST-optimised buffer. Samples were filled into hydrophilic capillaries (NanoTemper Technologies) for measurement.

### FACS analysis

Fluorescein-5-maleimide labeled CLR(4-Cl-F)T, and a scramble control were synthesized by combining fluorescein-5-maleimide with CLR(4-Cl-F)T or (4-Cl-F)CRTL in DMF. The resulting labeled peptides (at the indicated concentrations) were added to BHK-21 cells and incubated for 30 minutes. Cells were washed 3 times with DMEM plus 1% BSA (w/v). Fluorescence was measured by flow cytometry (BD LSRFortessa), with an excitation laser of 488 nm, and an emission bandpass filter of 530/30 nm.

### Fluorescence microscopy

The above fluorescein-labeled inhibitors (at the indicated concentrations) were added to BHK-21 cells and incubated for 30 minutes. Cells were washed 3 times with DMEM plus 1% BSA (w/v). Fluorescent micrographs were recorded on a confocal microscope (Zeiss). Excitation 488 nm, emission, 490LP filter.

### Toxin Neutralization Assay

J774 cells were plated at 2 × 10^5^ cells/ml (100 µL/well) in 10%DMEM and allowed to adhere for at least 1 hour at 37 °C and 5% CO_2_. PA (1.5 mL at 0.1 mg/mL) was mixed with LF (1.5 mL at 0.1 mg/mL) to generate LT. J774 plates were removed from the incubator, centrifuged and medium was removed. Solutions of LT (50 µL) plus various concentrations of the inhibitor, or scrambled control (20 µL, in PBS), were added to the cells and incubated overnight at 37 °C with 5% CO_2_. The next day, cell supernatants were removed and cell number determined.

### Data Availability

Data underpinning this study are openly available from the University of Southampton repository at https://doi.org/10.5258/SOTON/D0054.

## Electronic supplementary material

Supplementary Information
