# Supplementary material for: Targeting Bacillus anthracis toxicity with a genetically selected inhibitor of the PA/CMG2 protein-protein interaction
Source: Sci Rep. 2017 Jun 8;7:3104. doi: 10.1038/s41598-017-03253-3 (PMC5465072; doi:10.1038/s41598-017-03253-3)

Supplemental Information

Targeting *Bacillus anthracis* toxicity with a genetically selected inhibitor of the PA/CMG2 protein-protein interaction.

Abigail L. Male,[1] Fedor Forafonov,[1] Francesco Cuda,[1] Gong Zhang,[2] Siqi Zheng,[3] Petra C. F. Oyston,[4] Peng R. Chen,[2,3] E. Diane Williamson,[4] Ali Tavassoli[1,5,]*

1. Chemistry, University of Southampton, Southampton, SO17 1BJ, United Kingdom.

2 Peking-Tsinghua Center for Life Sciences, Academy for Advanced Interdisciplinary Studies, Peking University, Beijing, China.

3. Beijing National Laboratory for Molecular Sciences, Synthetic and Functional Biomolecules Center, College of Chemistry and Molecular Engineering, Peking University, Beijing, China.

4. Defence Science and Technology Laboratory, Porton Down, Salisbury, UK

5. Institute for Life Sciences, University of Southampton, Southampton, United Kingdom.

*E-mail: a.tavassoli@soton.ac.uk

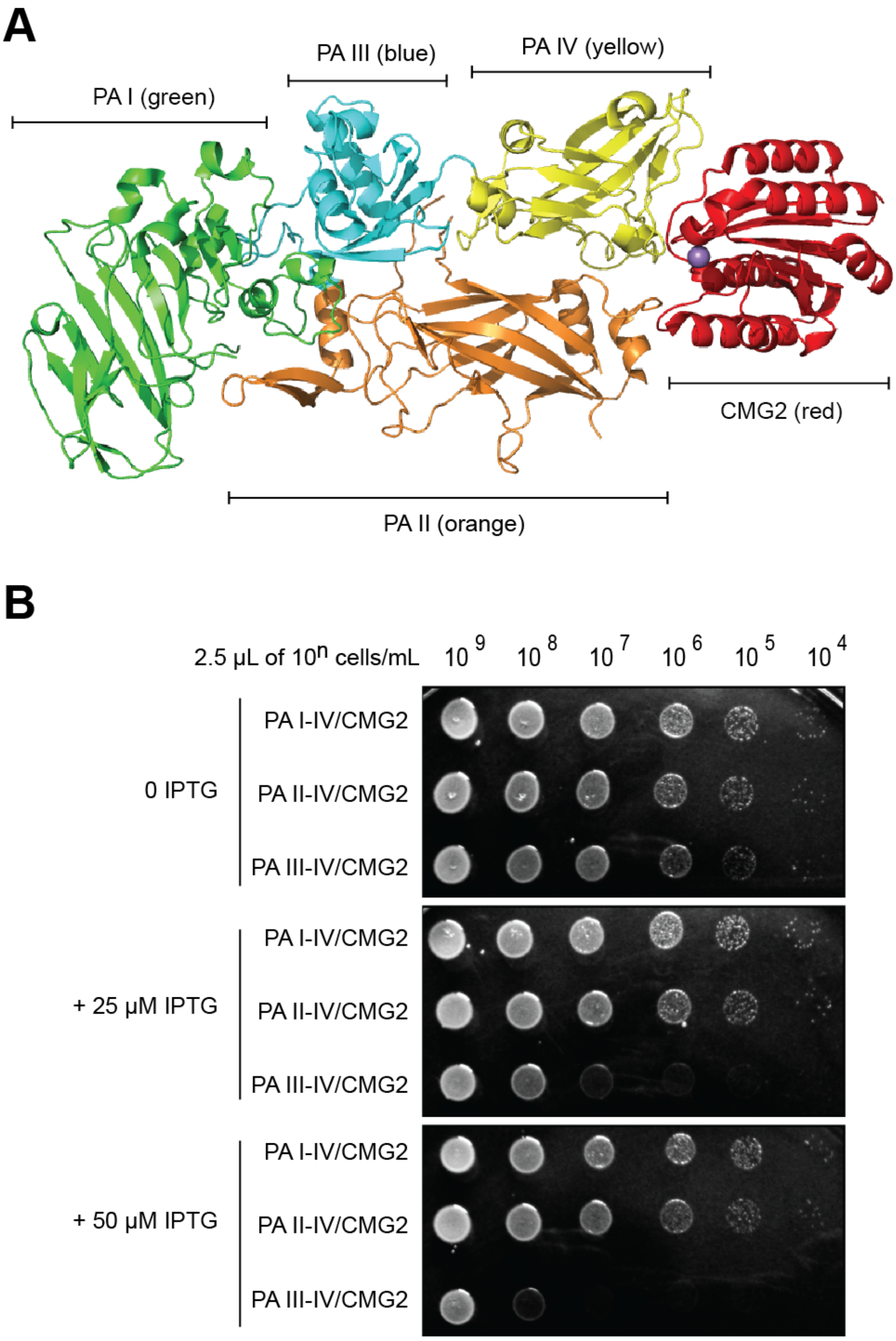

Supplemental Figure 1. A) The structure of PA bound to CMG2 from PDB 1T6B. The four domains of PA are highlighted in different colours for clarity. **B)** Drop-spotting data from the various RTHS built for this study. We assessed full length PA (I-IV, top row of each plate), PA II-IV (2nd row) and PA III-IV (bottom row). Only PA III-IV was observed to form a functional repressor with CMG2, as illustrated by the loss of growth of the PA III-IV/CMG2 RTHS (loss of 4 spots = 10000 fold) in response to 50 µM IPTG.

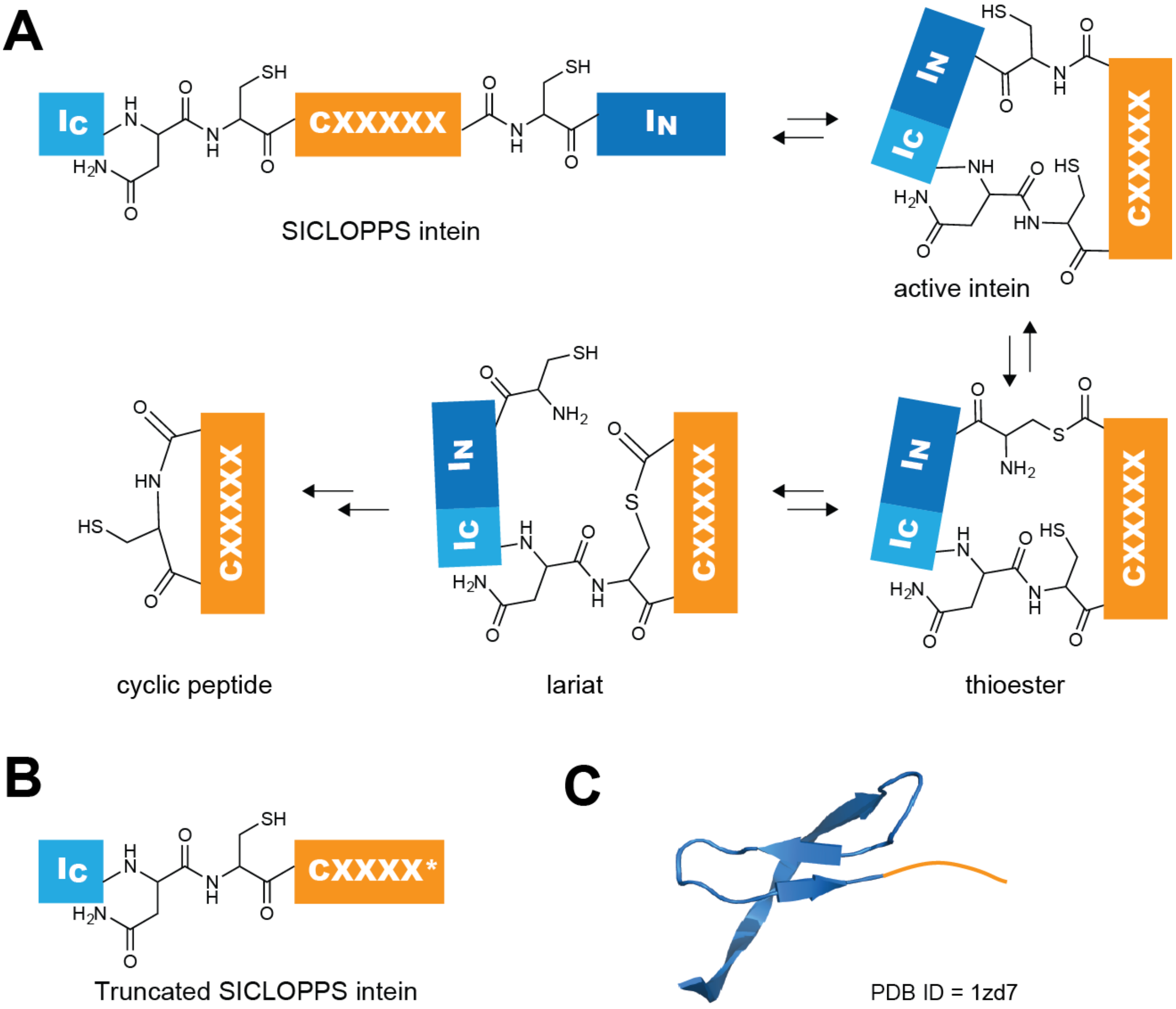

Supplemental Figure 2. A) The mechanism of SICLOPPS intein splicing. **B)** Cartoon representation of the truncated SICLOPPS inteins selected in this study. **C)** Graphical representation of the truncated SICLOPPS intein adapted from PDB ID 1ZD7.

Supplemental Table 1 Selected hits

Rank	Sequence
1	CMNHFPA*
1	CLRFT*
1	CLRPT*
2	CPLSLVA*
3	CPIF*
3	CITA*
3	CLIYLI
3	CHSMDL
3	CAHS*

*=STOP codon

Supplementary Experimental procedures and Spectra.
All peptides were synthesized and purified as detailed in the methods section.

CLRFT

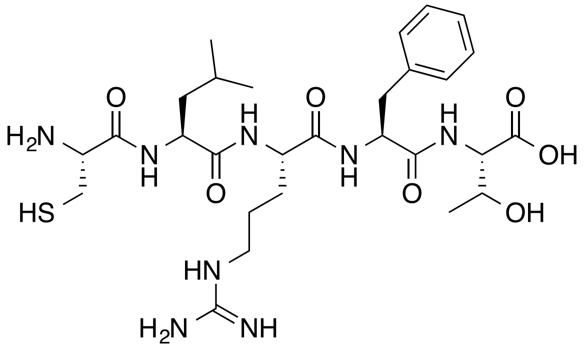

The peptide was synthesised using standard solid phase peptide synthesis techniques, with Fmoc-protected amino acids, yielding 54 mg (34%) of the product as a white solid. ^{1}H NMR (600 MHz, DMSO-d_6) δ ppm 12.62 (1 H, br. s., Thr-COOH) 8.51 (1 H, d, *J*=7.63 Hz, Leu-NH) 8.13 (1 H, d, *J*=8.24 Hz, Thr-NH) 8.12 (1 H, d, *J*=8.24 Hz, Cys-NH) 7.95 (1 H, d, *J*=7.93 Hz, Phe-NH) 7.51 (1 H, br. s., Arg-NH) 7.19 - 7.29 (4 H, m, Phe-ArH) 7.14 - 7.18 (1 H, m, Phe-ArH) 4.96 (1 H, br. s., Cys-SH or Thr-OH) 4.69 (1 H, td, *J*=8.54, 4.27 Hz, Phe-αH) 4.28 - 4.38 (1 H, m, Leu-αH) 4.20 - 4.27 (2 H, m, Thr-αH and Thr-βH) 4.15 - 4.19 (1 H, m, Arg-αH) 3.97 - 4.06 (1 H, m, Cys-αH) 3.35 (br. s., solvent- H_2O) 2.95 - 3.12 (4 H, m, Arg-βH, Arg-δH or Phe-βH) 2.76 - 2.88 (2 H, m, Arg-βH or Phe-βH) 1.62 (2 H, d, *J*=5.19 Hz, Cys-βH) 1.35 - 1.51 (5 H, m, Leu-βH, Arg-γH and Leu-γH) 1.00 - 1.10 (3 H, m, Thr-γH) 0.81 - 0.91 (6 H, m, Leu-δH); Analytical HPLC (220 nm) 18.1 min; IR (neat) 3270, 1631, 1525, 1188 cm^{-1}; MS (ESI+) *m/z* (%) 639.2 ((M + H)$^+$, 100), 320.5 ((M + 2H)$^{2+}$ 41.8); HRMS (ESI+) for $C_{28}H_{47}N_8O_7S$ (M + H)$^+$ calcd 639.3283, found 639.3270.

HPLC:

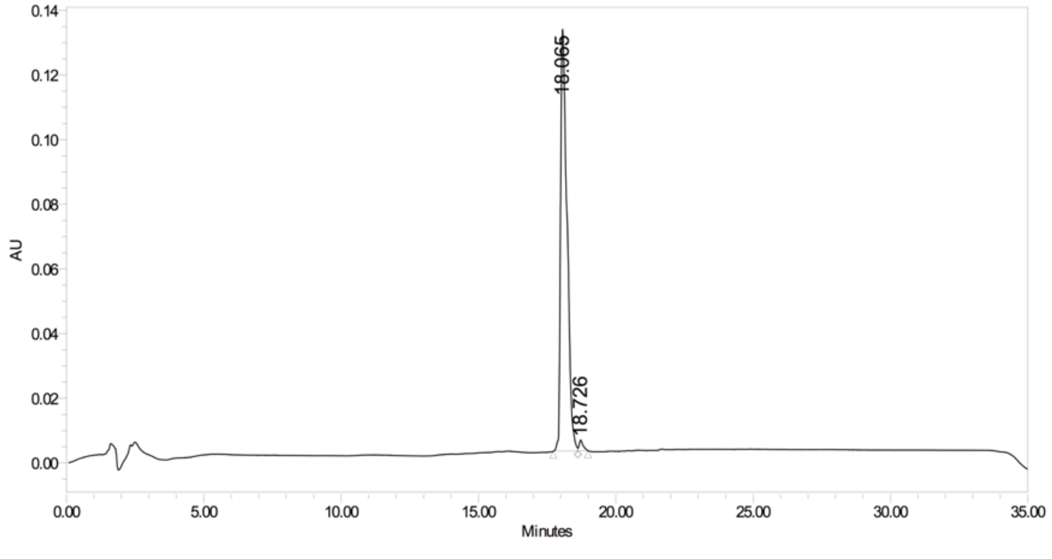

Mass Spectrum:

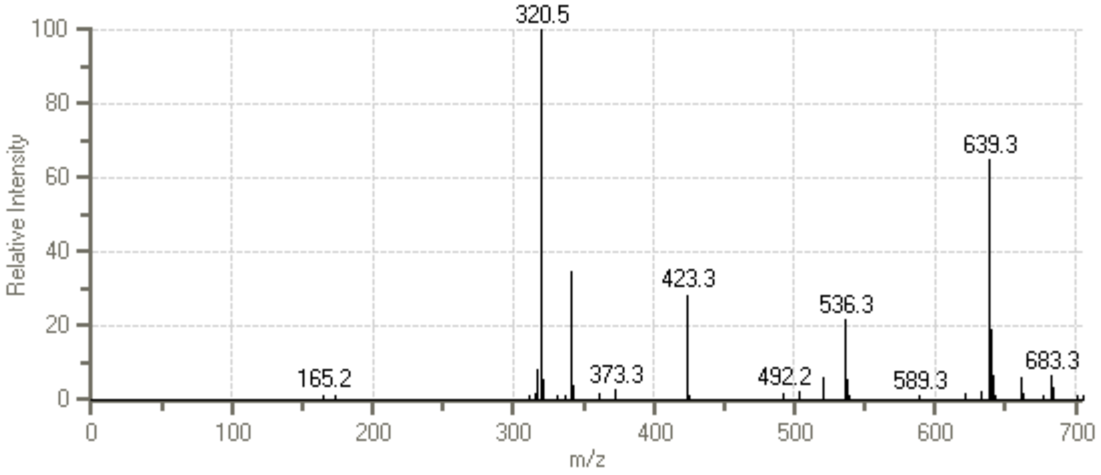

High resolution mass spectrum:

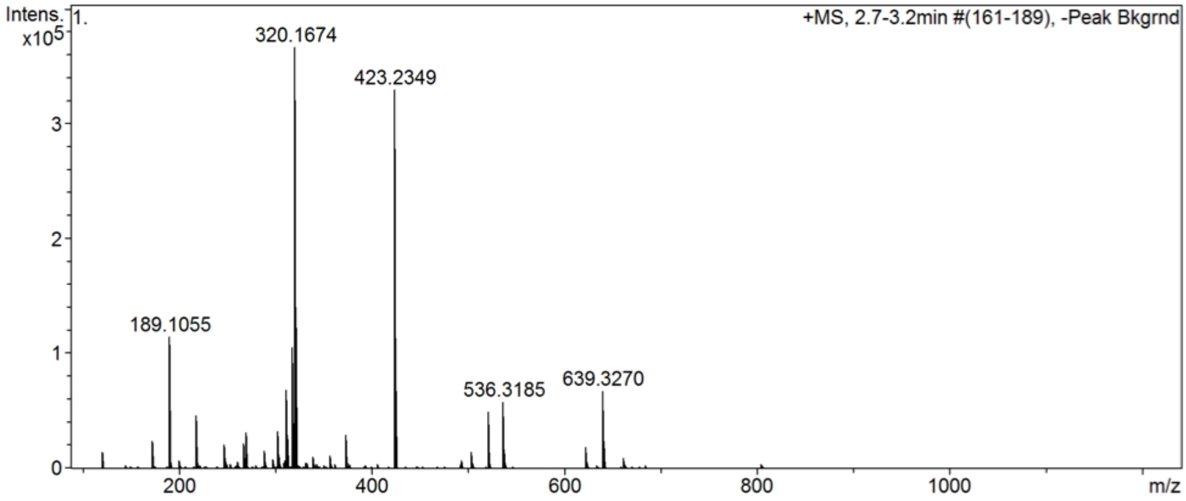

^{1}H NMR:

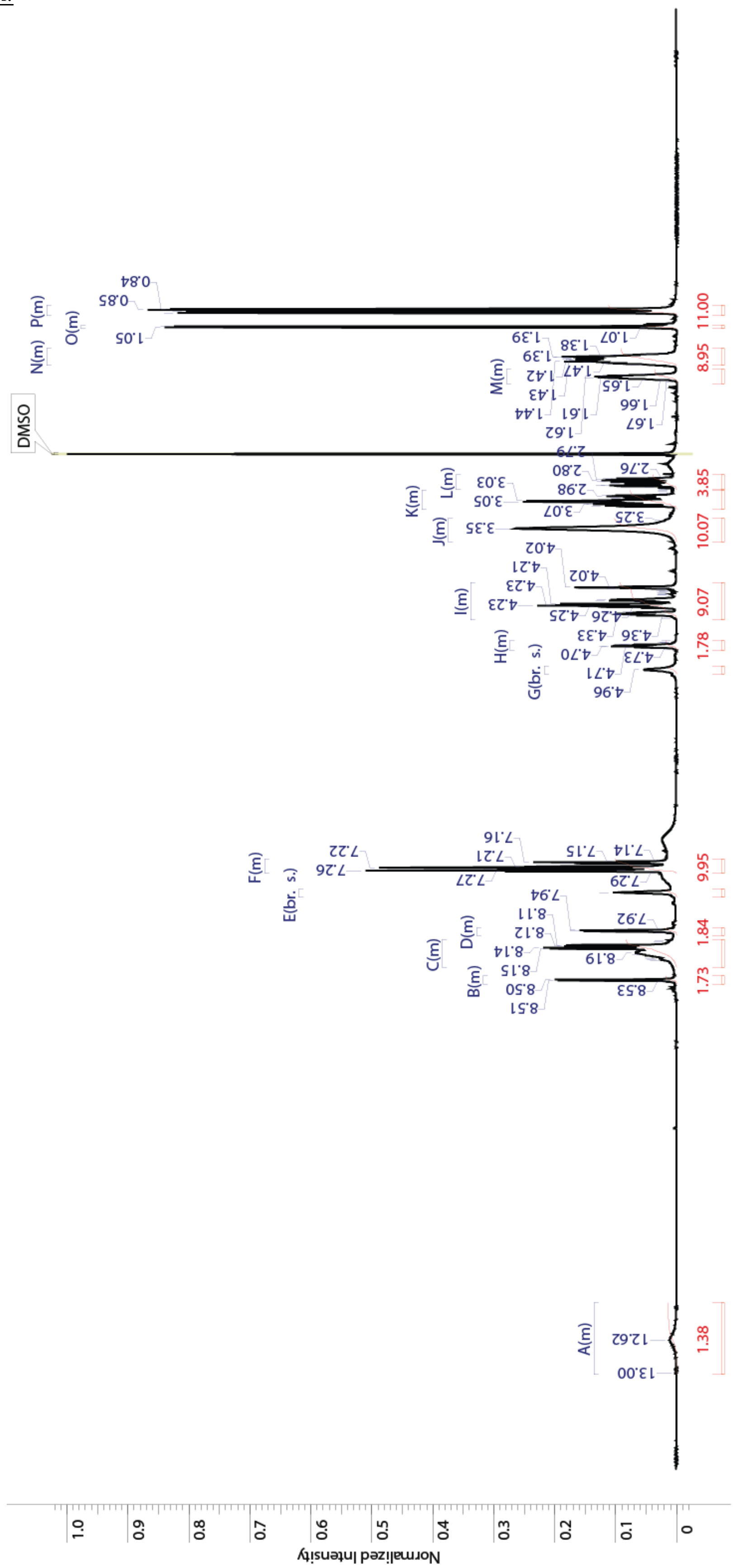
DMSO
A(m)
B(m)
C(m)
D(m)
E(br. s.)
F(m)
G(br. s.)
H(m)
I(m)
J(m)
K(m)
L(m)
M(m)
N(m)
O(m)
P(m)
13.00
12.62
8.53
8.51
8.50
8.19
8.15
8.14
8.12
8.11
7.94
7.92
7.29
7.27
7.26
7.22
7.21
7.16
7.15
7.14
4.96
4.73
4.71
4.70
4.36
4.33
4.26
4.25
4.23
4.23
4.21
4.02
4.02
3.35
3.25
3.07
3.05
3.03
2.98
2.80
2.79
2.76
1.67
1.66
1.65
1.62
1.61
1.47
1.44
1.43
1.42
1.39
1.39
1.38
1.07
1.05
0.85
0.84
1.38
1.73
1.84
9.95
1.78
9.07
10.07
3.85
8.95
11.00
Normalized Intensity
1.0
0.9
0.8
0.7
0.6
0.5
0.4
0.3
0.2
0.1
0

COSY:

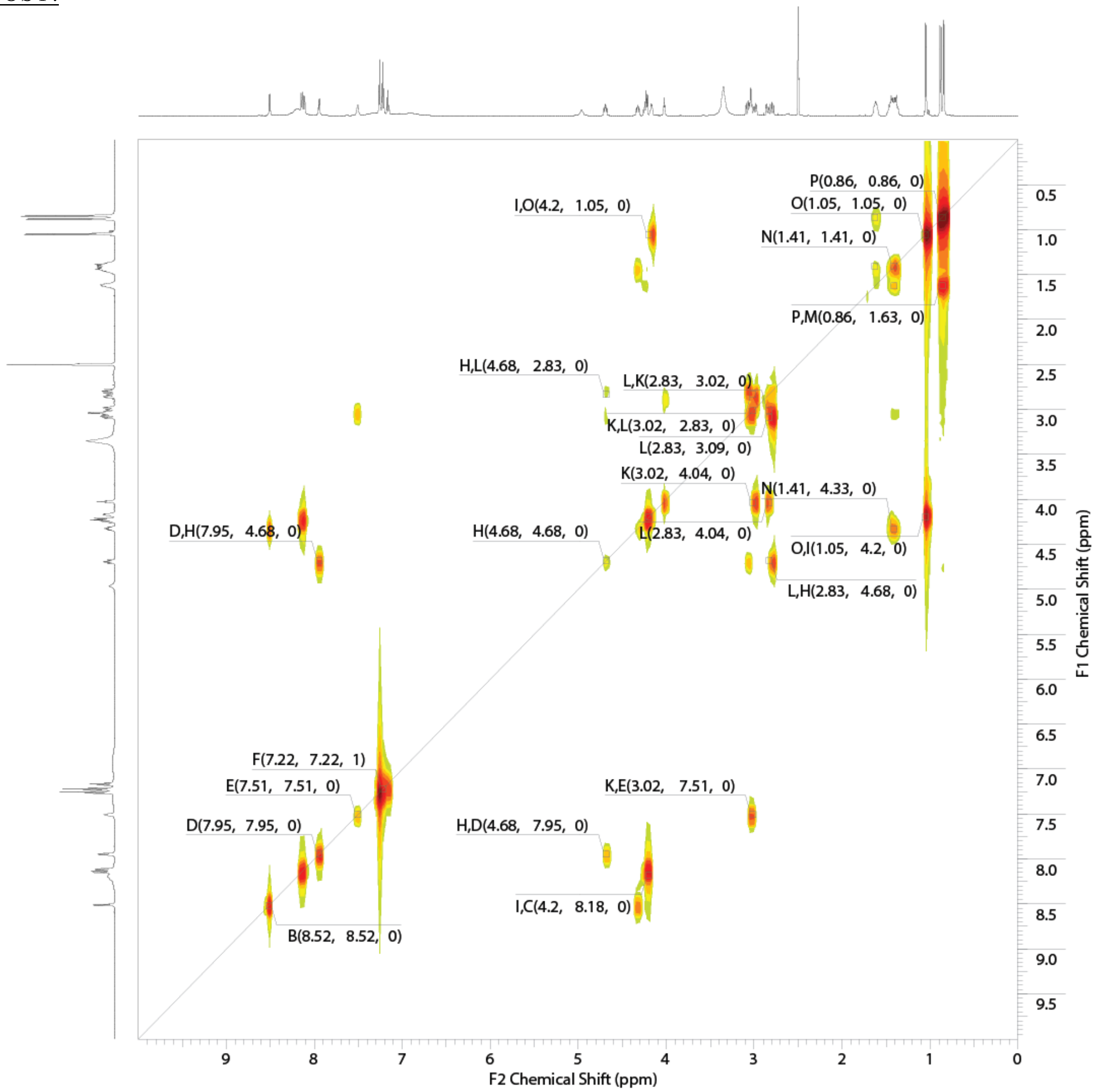
P(0.86, 0.86, 0)
O(1.05, 1.05, 0)
I,O(4.2, 1.05, 0)
N(1.41, 1.41, 0)
P,M(0.86, 1.63, 0)
H,L(4.68, 2.83, 0)
L,K(2.83, 3.02, 0)
K,L(3.02, 2.83, 0)
L(2.83, 3.09, 0)
K(3.02, 4.04, 0)
N(1.41, 4.33, 0)
D,H(7.95, 4.68, 0)
H(4.68, 4.68, 0)
L(2.83, 4.04, 0)
O,I(1.05, 4.2, 0)
L,H(2.83, 4.68, 0)
F(7.22, 7.22, 1)
E(7.51, 7.51, 0)
K,E(3.02, 7.51, 0)
D(7.95, 7.95, 0)
H,D(4.68, 7.95, 0)
I,C(4.2, 8.18, 0)
B(8.52, 8.52, 0)
F2 Chemical Shift (ppm)
F1 Chemical Shift (ppm)

TOCSY:

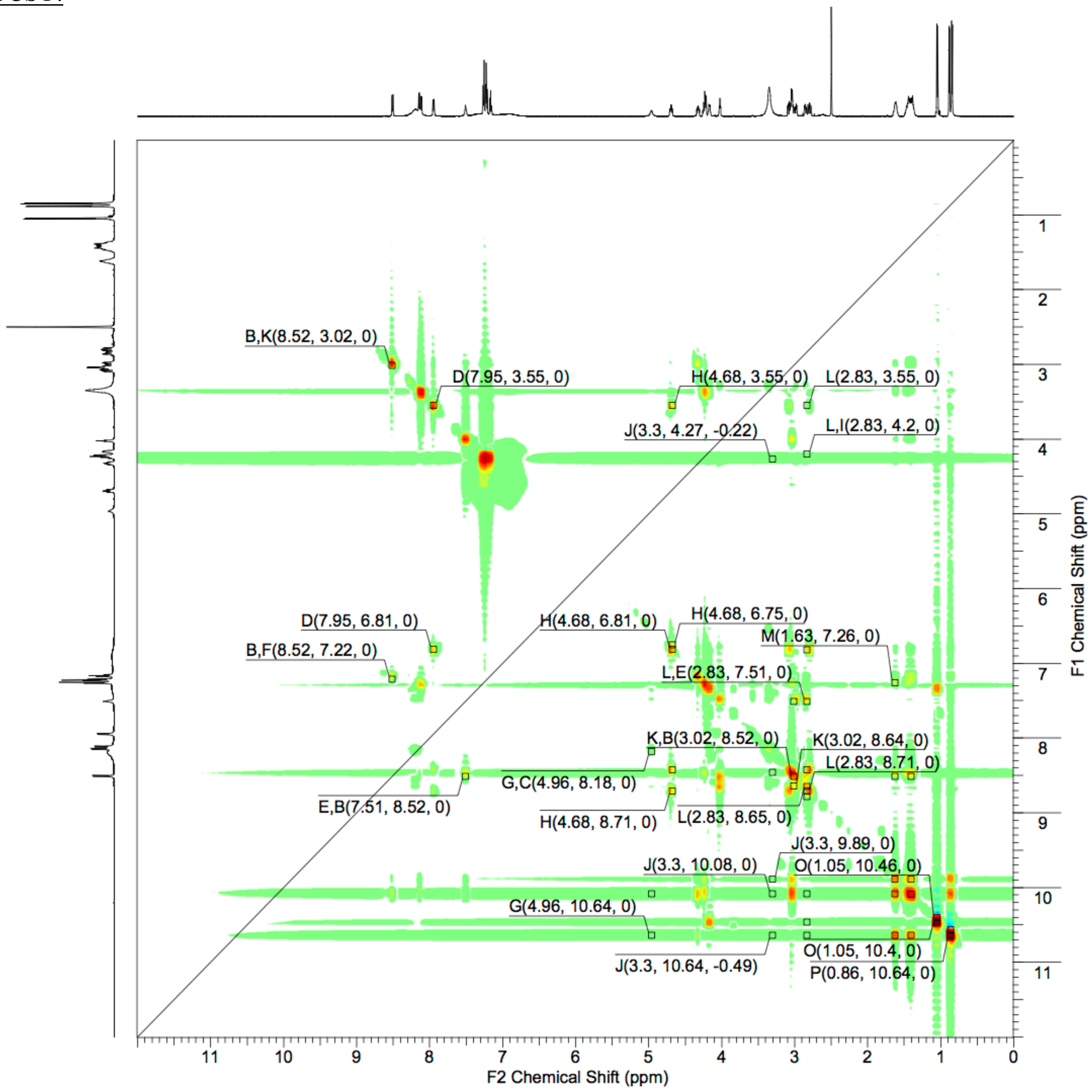
B,K(8.52, 3.02, 0)
D(7.95, 3.55, 0)
H(4.68, 3.55, 0)
L(2.83, 3.55, 0)
J(3.3, 4.27, -0.22)
L,I(2.83, 4.2, 0)
D(7.95, 6.81, 0)
H(4.68, 6.81, 0)
H(4.68, 6.75, 0)
M(1.63, 7.26, 0)
B,F(8.52, 7.22, 0)
L,E(2.83, 7.51, 0)
K,B(3.02, 8.52, 0)
K(3.02, 8.64, 0)
L(2.83, 8.71, 0)
G,C(4.96, 8.18, 0)
E,B(7.51, 8.52, 0)
H(4.68, 8.71, 0)
L(2.83, 8.65, 0)
J(3.3, 9.89, 0)
J(3.3, 10.08, 0)
O(1.05, 10.46, 0)
G(4.96, 10.64, 0)
O(1.05, 10.4, 0)
J(3.3, 10.64, -0.49)
P(0.86, 10.64, 0)
F1 Chemical Shift (ppm)
F2 Chemical Shift (ppm)

CLRPT

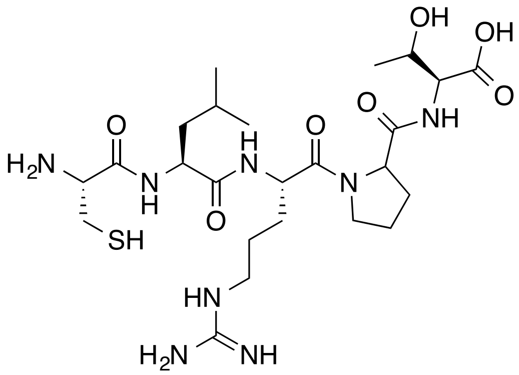

The peptide was synthesised using standard solid phase peptide synthesis techniques, with Fmoc-protected amino acids, yielding 21.0 mg (15%) of the product as a white solid. ^{1}H NMR (600 MHz, DMSO-d_6) δ ppm 12.43 - 12.71 (1 H, m, Thr-COOH) 8.52 (1 H, d, *J*=7.93 Hz, Leu-NH) 8.25 (1 H, d, *J*=7.63 Hz, Arg-NH) 8.20 (3 H br. s., Phe-NH) 7.86 (1 H, d, *J*=8.24 Hz, Pro-NH) 7.53 (1 H, t, *J*=5.49 Hz, Arg-$NH_{side-chain}$) 4.91 (1 H, br. s., Thr-OH) 4.52 (1 H, dd, *J*=8.39, 3.51 Hz, Pro-αH) 4.45 - 4.50 (1 H, m, Arg-αH) 4.36 (1 H, ddd, *J*=9.84, 7.86, 5.19 Hz, Leu-αH) 4.13 - 4.18 (2 H, m, Thr-αH, Thr-βH) 4.02 - 4.07 (1 H, m, Cys-αH) 3.61 - 3.68 (1 H, m, Pro-βH) 3.02 - 3.15 (3 H, m, Arg-δH) 2.99 (1 H, d, *J*=5.19 Hz, Cys-βH) 2.88 (1 H, d, *J*=14.04 Hz, Cys-βH) 2.00 - 2.12 (1 H, m, Pro-γH) 1.82 - 1.96 (3 H, m, Pro-γH and Pro-δH) 1.62 - 1.73 (2 H, m, Leu-βH) 1.49 - 1.58 (3 H, m, Arg-γH and Leu-γH) 1.42 - 1.49 (2 H, m, Arg-βH) 1.06 - 1.09 (3 H, m, Thr-γH) 0.89 (6 H, d, *J*=6.71 Hz, Leu-δH); Analytical HPLC (220 nm) 16.1 min; IR (neat) 3278, 1655, 1182 cm^{-1}; MS (ESI+) *m/z* (%) 589.2 ((M + H)$^{+}$), 295.2 ((M +2H)$^{2+}$); HRMS **(**ESI+) for $C_{24}H_{45}N_8O_7S$ (M + H)$^{+}$ calcd 589.3126, found 589.3125.

HPLC:

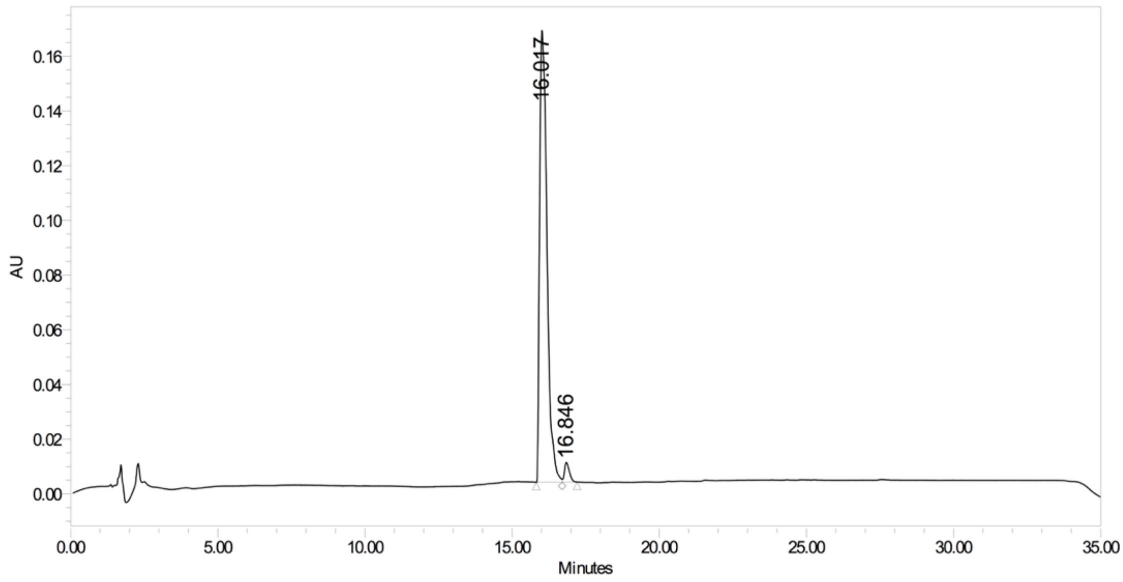

Mass Spectrum:

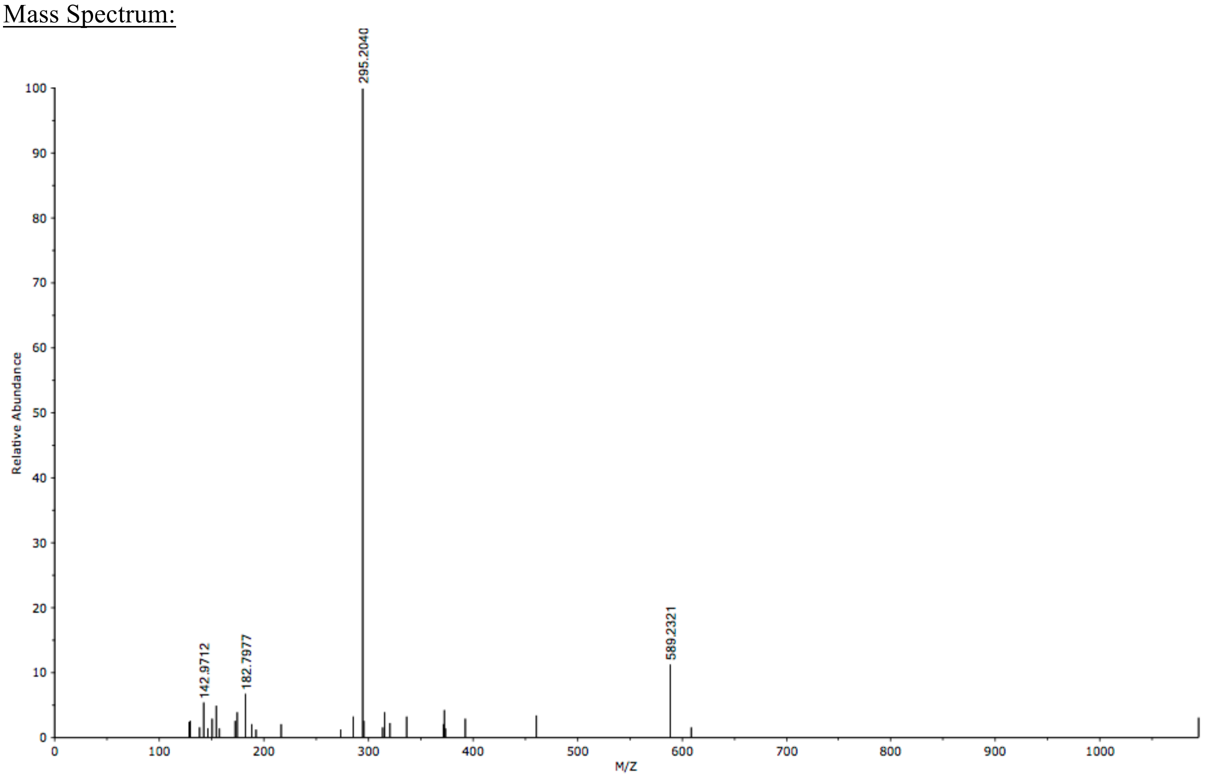

High resolution mass spectrum:

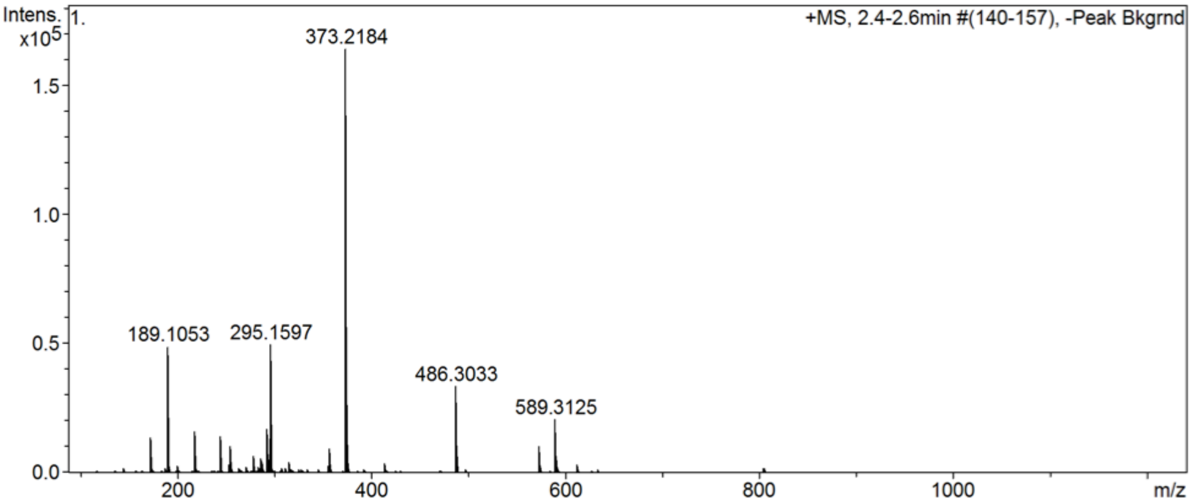

^{1}H NMR:

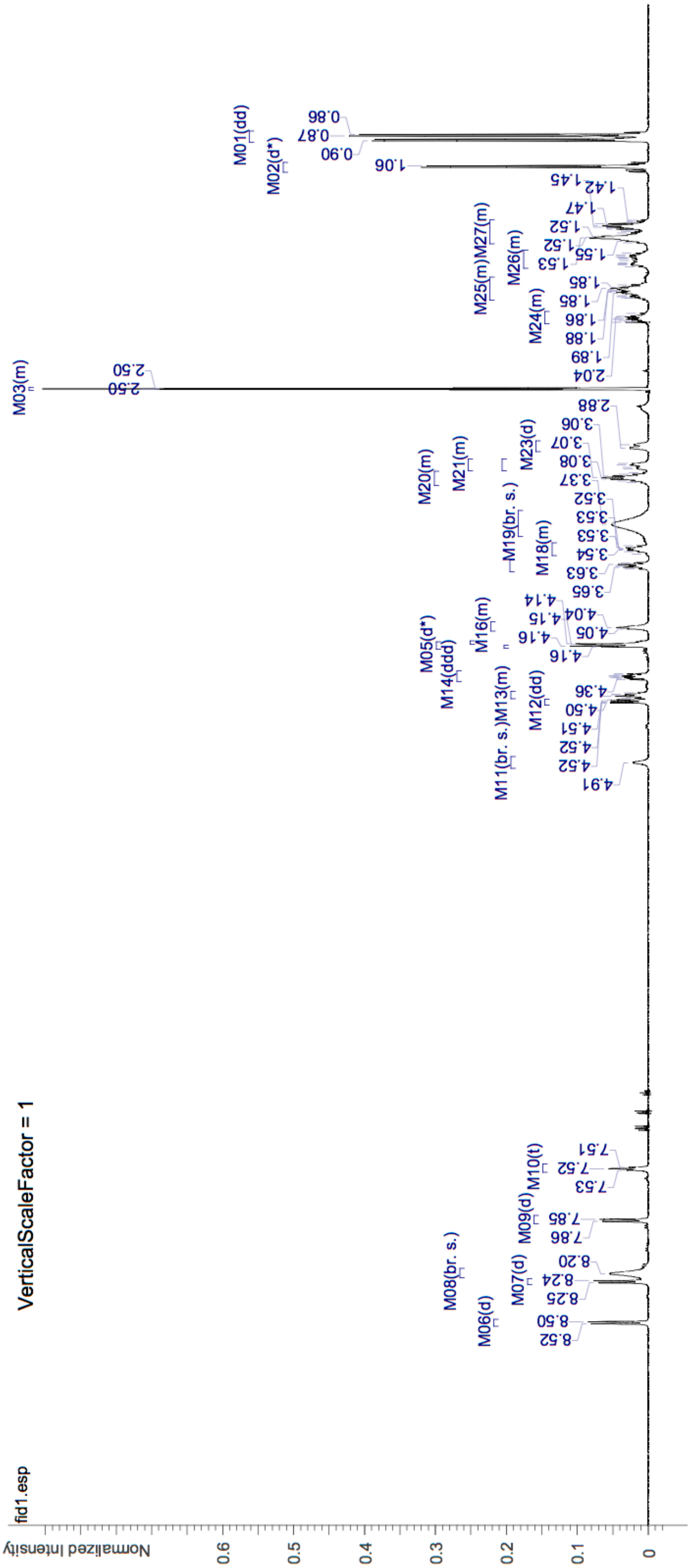

fid1.esp
VerticalScaleFactor = 1
Normalized Intensity
0
0.1
0.2
0.3
0.4
0.5
0.6

CMNHFPA

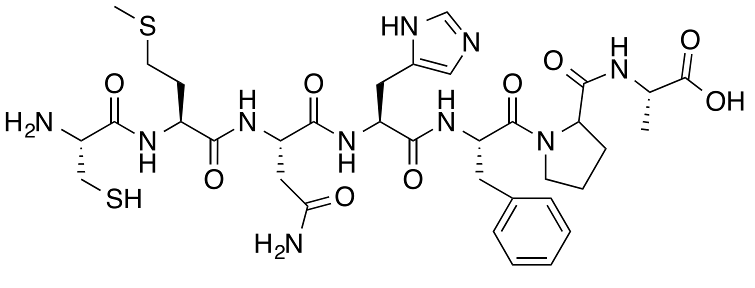

The peptide was synthesised using standard solid phase peptide synthesis techniques, with Fmoc-protected amino acids, yielding 56 mg (27%) of the product as a white solid. ^{1}H NMR (600MHz, DMSO) δ ppm 12.57 (1H, br. s., Ala-COOH) 8.93 (2H, br. s., His-$NH_{side-chain}$) 8.63 (1H, d, J = 8.5 Hz, Met-NH) 8.33 (2H, t, J = 7.9 Hz, Asn-NH and His-NH) 8.09 (1H, d, J = 7.3 Hz, Ala-NH) 8.03 (1H, d, J = 7.3 Hz, Phe-NH) 7.46 (1H, br. s., His-IndH) 7.42 (1H, m, Phe-ArH) 7.24 - 7.33 (4H, m, Phe-ArH) 7.16 - 7.25 (1H, m, His-IndH) 6.97 - 7.06 (1H, m, Cys-SH) 4.57 - 4.64 (1H, dd, His-αH) 4.46 - 4.56 (1H, m, Phe-αH) 4.39 - 4.45 (1H, m, J=8.5 Hz, Asn-αH) 4.37 (1H, d, J = 8.5 Hz, Met-αH) 4.10 - 4.25 (1H, dd, J=7.3 Hz, Ala-αH) 4.03 (1H, t, J=6, Cys-αH) 3.94 (1H, d, J = 6.1 Hz, Pro-αH) 3.53 - 3.64 (1H, m, Pro-βH) 3.14 - 3.50 (H, m, Pro-βH and Phe-βH) 3.03 (2H, d, J = 15.9 Hz, His-βH) 2.97 (2H, m, Asn-βH) 2.76 - 2.92 (3H, m, Cys-βH) 2.35 - 2.49 (4H, m, Met-γH and Pro-γH) 1.97 - 2.07 (3H, m, Met-δH) 1.81 - 1.95 (3H, m, Met-βH) 1.71 - 1.80 (2H, m, Pro-δH) 1.28 (3H, d, J = 7.3 Hz, Ala-βH); Analytical HPLC (220 nm) 17.6 min; IR (neat) 3279, 1631, 1188 cm^{-1}; MS (ESI+) *m/z* (%) 819.2 ((M + H)$^+$, 100.0), 410.4 ((M +2H)$^{2+}$ 68.0); HRMS (ESI+) for $C_{35}H_{50}N_{10}O_9S_2$ (M + H)$^+$ calcd 819.3276, found 819.3828.

HPLC:

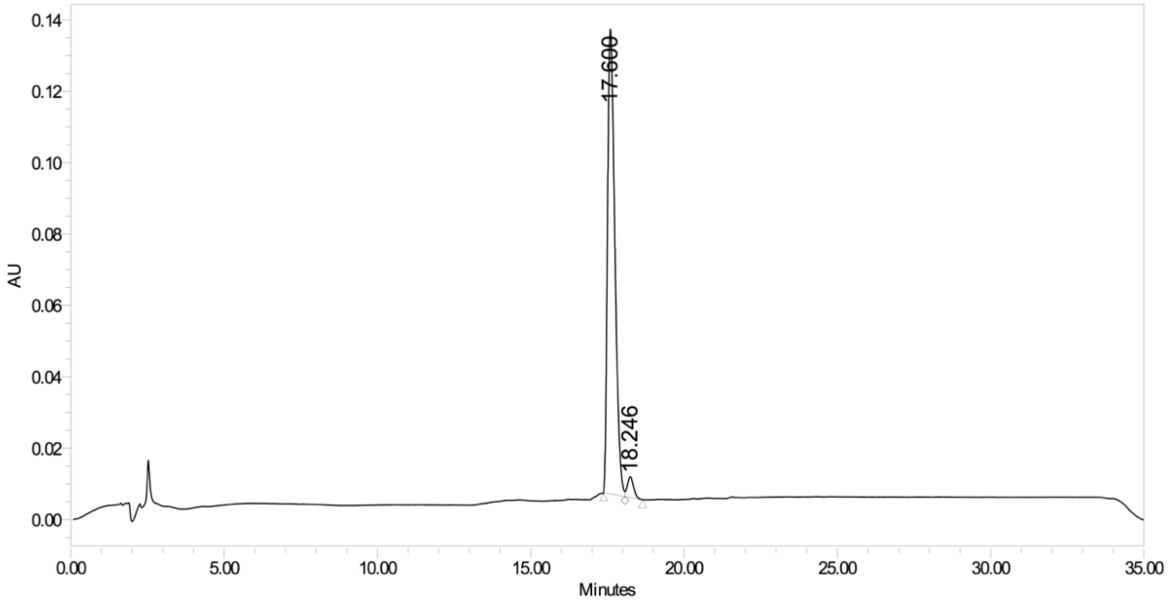

Mass Spectrum

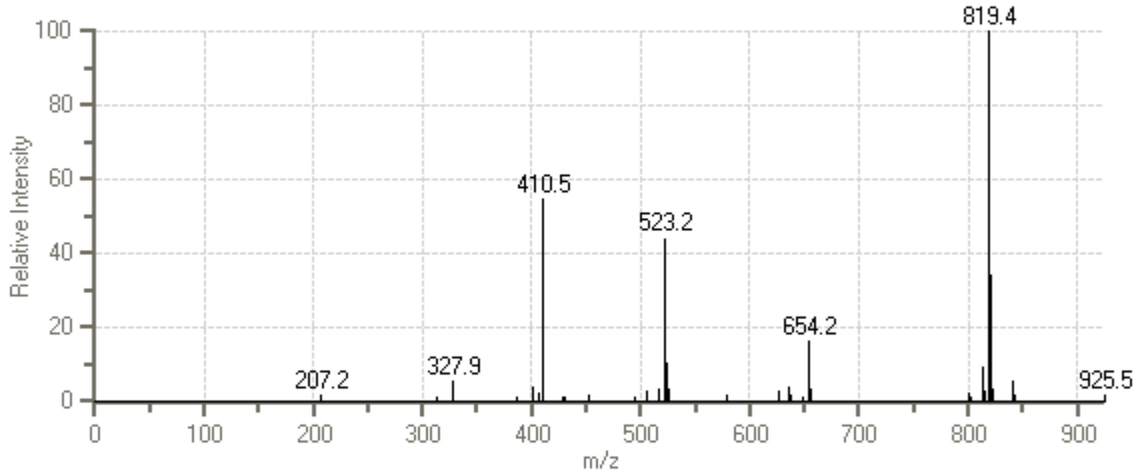

High resolution mass spectrum:

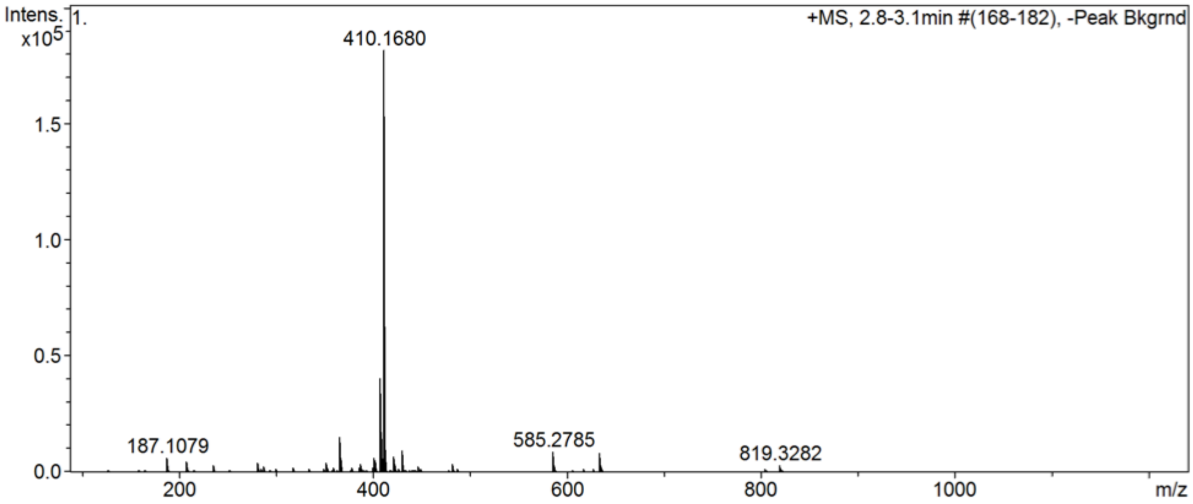

^{1}H NMR:

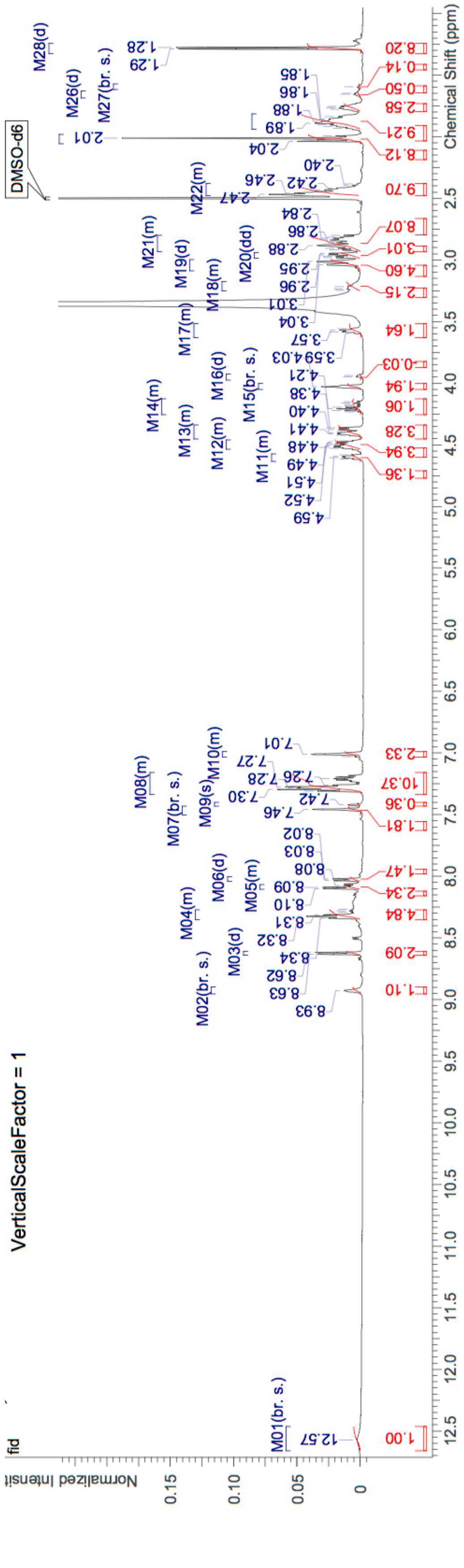
VerticalScaleFactor = 1
fid
DMSO-d6
Chemical Shift (ppm)
Normalized Intensi

FCRTL

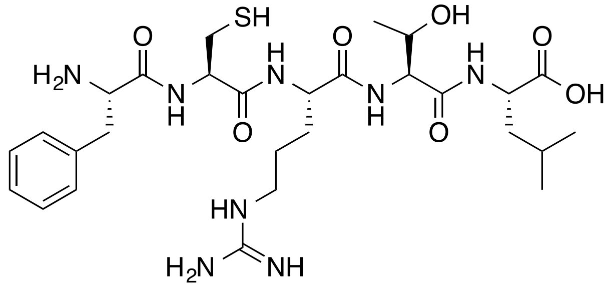

The peptide was synthesised using standard solid phase peptide synthesis techniques, with Fmoc-protected amino acids, yielding 56 mg (35%) of the product as a white solid. ^{1}H NMR (600 MHz, DMSO-d_6) δ ppm 8.78 (1 H, d, J=9.77 Hz, Cys-NH) 8.39 (1 H, d, J=7.32 Hz, Leu-NH) 8.09 - 8.17 (4 H, m, Phe-NH) 7.99 (1 H, d, J=7.32 Hz, Arg-NH) 7.82 (1 H, d, J=7.32 Hz, Thr-NH) 7.60 (1 H, br. s., Arg-NH$_{side-chain}$) 7.30 - 7.33 (2 H, m, Phe-ArH) 7.23 - 7.27 (3 H, m, Phe-ArH) 4.83 (1 H, br. s., Cys-SH) 4.50 - 4.54 (1 H, m, Cys-αH) 4.35 (1 H, q, J=7.32 Hz, Leu-αH) 4.19 - 4.30 (2 H, m, Thr-αH, Phe-αH) 4.14 (1 H, br. s., Thr-OH) 3.90 - 3.97 (2 H, m, Thr-βH, Arg-αH) 3.04 - 3.13 (4 H, m, Arg-δH, Phe- βH) 2.88 - 2.95 (1 H, m, Arg-γH) 2.67 - 2.82 (2 H, m, Arg-γH, Cys-βH) 2.41 - 2.48 (1 H, m, Cys-βH) 1.72 (1 H, br. s.) 1.61 - 1.68 (1 H, m, Leu-βH) 1.49 - 1.56 (6 H, m, Leu-γH, Leu-βH) 1.06 (4 H, d, J=7.32 Hz, Thr-γH) 0.88 (5 H, d, J=7.32 Hz, Leu-δH) 0.83 (4 H, d, J=4.88 Hz, Leu-δH); Analytical HPLC (220 nm) 17.4 min; IR (neat) 3280, 1636, 1134 cm^{-1}; MS (ESI+) m/z (%) 639.3 ((M + H)$^+$, 61.3), 320.5 ((M +2H)$^{2+}$ 100.0); HRMS (ESI+) for $C_{28}H_{46}N_8O_7S$ (M + H)$^+$ calcd 639.3283, found 639.3271.

HPLC:

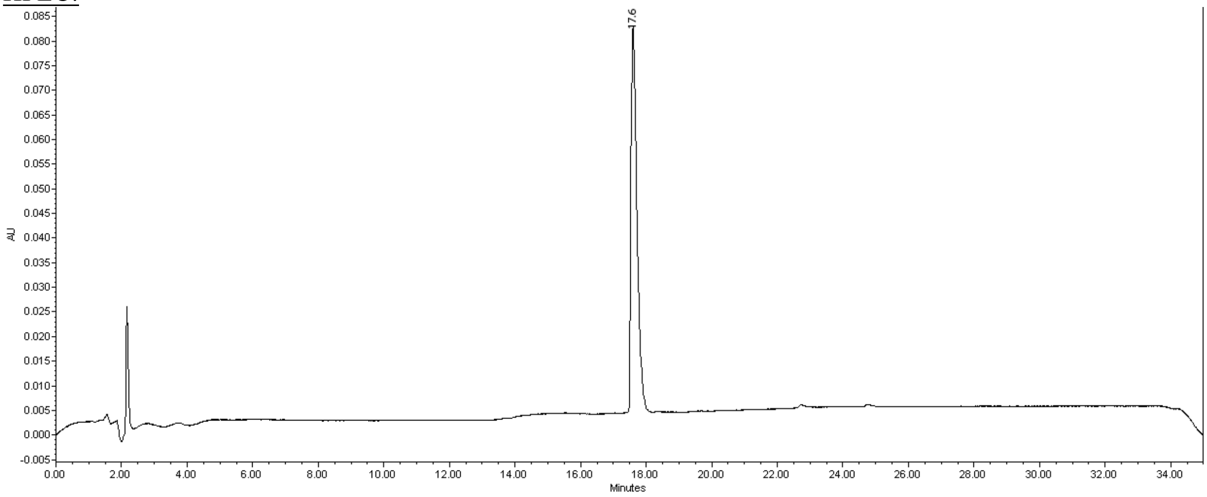

Mass Spectrum:

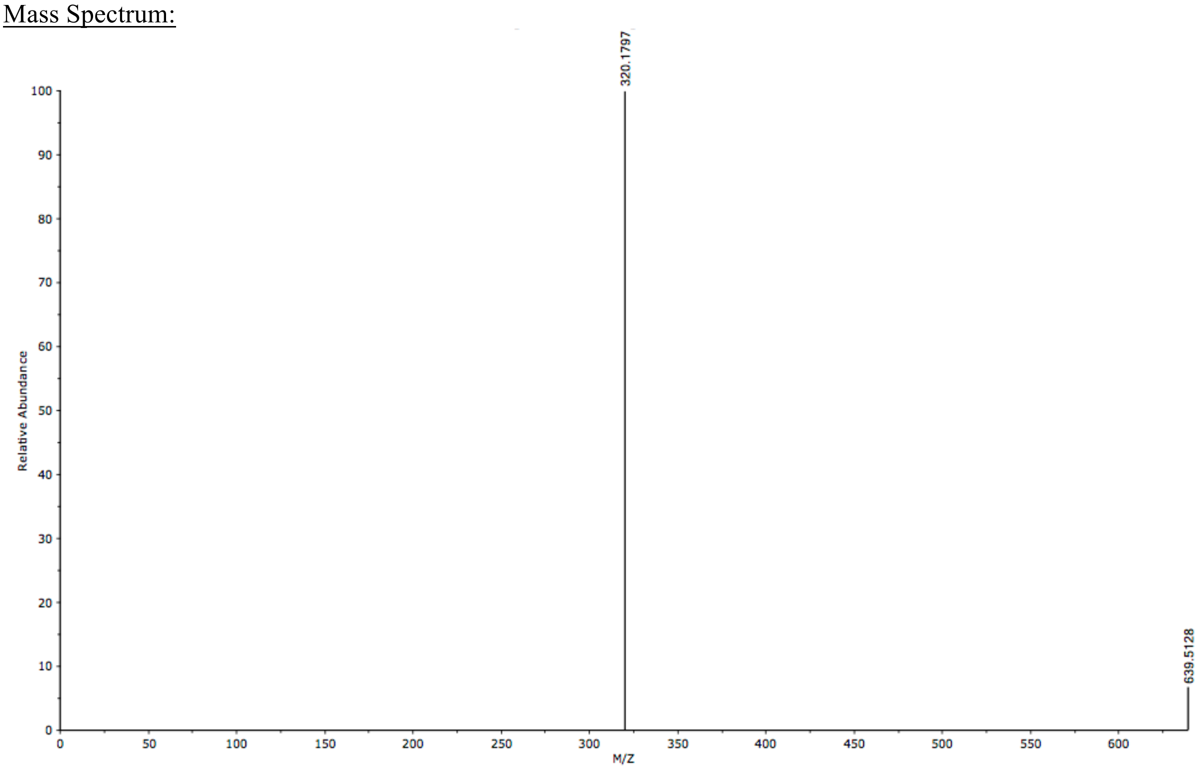

High resolution mass spectrum:

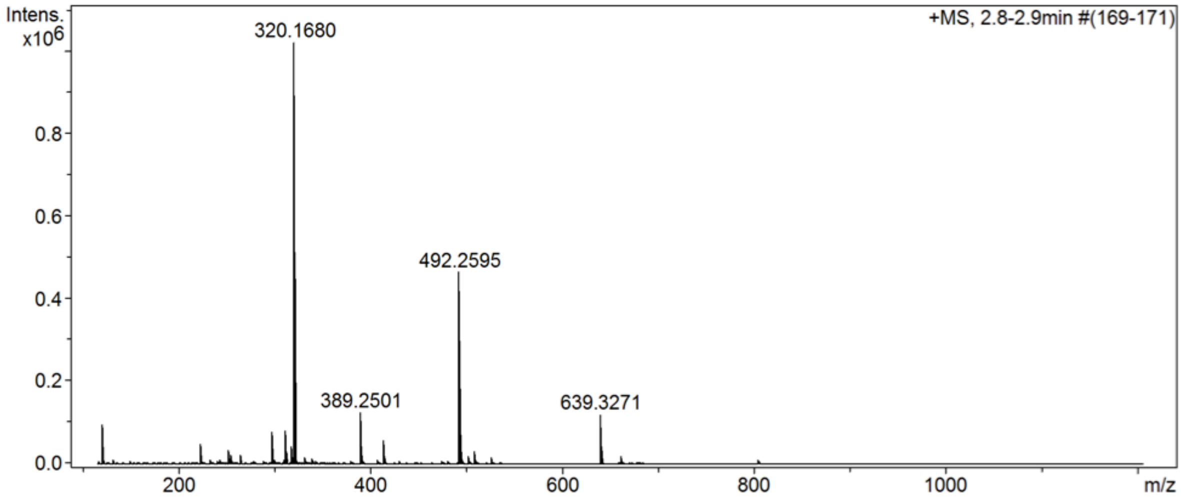

^{1}H NMR:

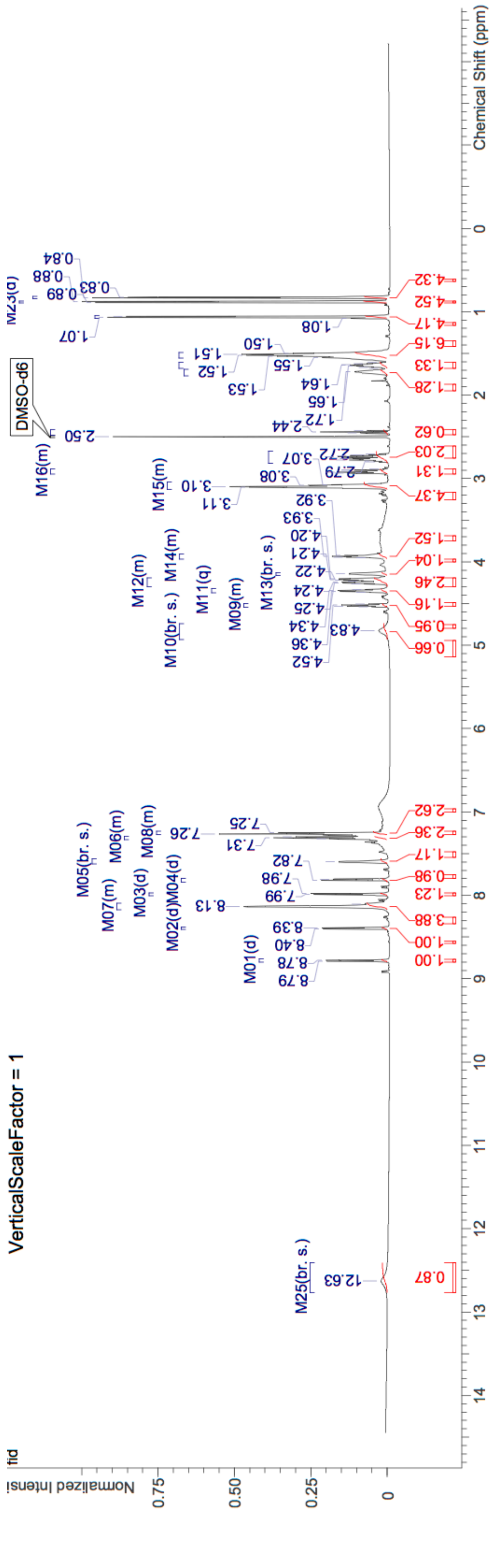
VerticalScaleFactor = 1
Chemical Shift (ppm)
Normalized Intensi
fid
DMSO-d6
2.50
M25(br. s.)
12.63
M01(d)
8.79
8.78
8.40
8.39
8.13
7.99
7.98
7.82
7.31
7.26
7.25
M02(d)M04(d)
M03(d)
M07(m)
M05(br. s.)
M06(m)
M08(m)
M09(m)
M10(br. s.)
M11(q)
M12(m)
M13(br. s.)
M14(m)
M15(m)
M16(m)
4.83
4.52
4.36
4.34
4.25
4.24
4.22
4.21
4.20
3.93
3.92
3.11
3.10
3.08
3.07
2.79
2.72
2.44
1.72
1.65
1.64
1.55
1.53
1.52
1.51
1.50
1.08
1.07
0.89
0.88
0.84
0.83
0.87
1.00
1.00
3.88
1.23
0.98
1.17
2.36
2.62
0.66
0.95
1.16
2.46
1.04
1.52
4.37
1.31
2.03
0.62
1.28
1.33
6.15
4.17
4.52
4.32

HPCNAMF

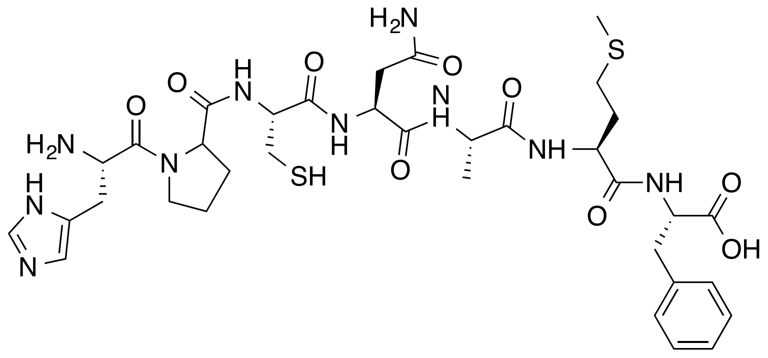

The peptide was synthesised using standard solid phase peptide synthesis techniques, with Fmoc-protected amino acids, yielding 45 mg (22%) of the product as a white solid. ^{1}H NMR (600 MHz, DMSO-d_6) δ ppm 8.92 (1 H, br. s., His-NH$_{\text{side-chain}}$) 8.54 (1 H, d, *J*=4.39 Hz, Pro-NH) 8.23 (2 H, t, *J*=7.32 Hz, His-NH) 8.14 (1 H, d, *J*=7.32 Hz, Cys-NH) 8.04 (1 H, d, *J*=7.32 Hz, Asn-NH) 7.95 - 8.02 (2 H, m, Met-NH and Phe-NH) 7.93 (1 H, d, *J*=7.32 Hz, Ala-NH) 7.45 (2 H, d, *J*=10.25 Hz, His-ArH) 7.23 - 7.28 (3 H, m, Phe-ArH, Asn-NH$_{2\text{-side-chain}}$) 7.16 - 7.22 (4 H, m, Phe-ArH) 6.98 (1 H, br. s. His-ArH) 4.51 - 4.55 (1 H, m, Cys-SH) 4.48 (1 H, d, *J*=7.32 Hz, His-αH) 4.37 - 4.45 (4 H, m, Pro-αH, Cys-αH, Asn-αH) 4.26 - 4.33 (1 H, m, Met-αH and Phe-αH) 4.21 (1 H, dd, *J*=7.32, 2.93 Hz, Ala-αH) 3.65 - 3.74 (2 H, m, Pro-δH) 3.31 - 3.58 (19 H, m, Pro-δH) 3.17 - 3.23 (2 H, m) 3.02 - 3.16 (4 H, m, Cys-βH, Asn-βH) 2.86 - 2.93 (1 H, m, Asn-βH) 2.77 - 2.86 (2 H, m, Cys-βH) 2.70 - 2.77 (1 H, m) 2.52 - 2.60 (1 H, m, His-βH) 2.46 (2 H, dd, *J*=16.11, 7.32 Hz, Phe-βH) 2.34 - 2.42 (3 H, m) 2.13 - 2.25 (3 H, m, Pro-βH, Met-βH) 2.01 (3 H, s, Met-δH) 1.96 (2 H, s) 1.83 - 1.90 (4 H, m, Pro-γH) 1.74 - 1.81 (1 H, m, Phe-βH) 1.67 - 1.73 (1 H, m) 1.55 - 1.64 (1 H, m, Met-βH) 1.13 - 1.20 (5 H, m, Ala-βH); Analytical HPLC (220 nm) 17.8 min; IR (neat) 3261, 1622, 1132 cm^{-1}; MS (ESI+) *m/z* (%) 819.4 ((M + H)$^{+}$, 100.0); HRMS (ESI+) for $C_{35}H_{50}N_{10}O_9S_2$ (M + H)$^{+}$ calcd 819.3276, found 819.3282.

HPLC:

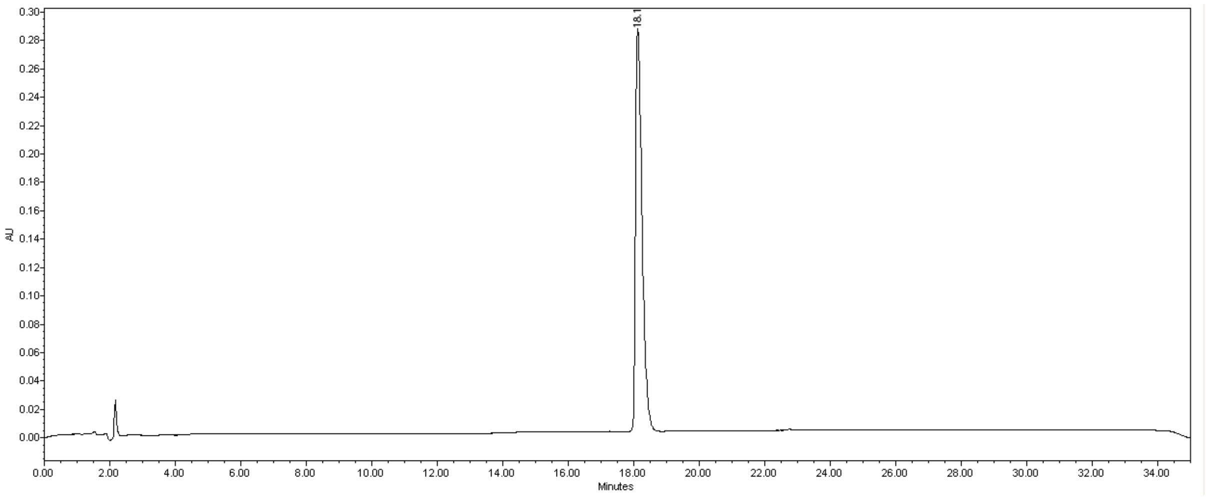

Mass Spectrum:

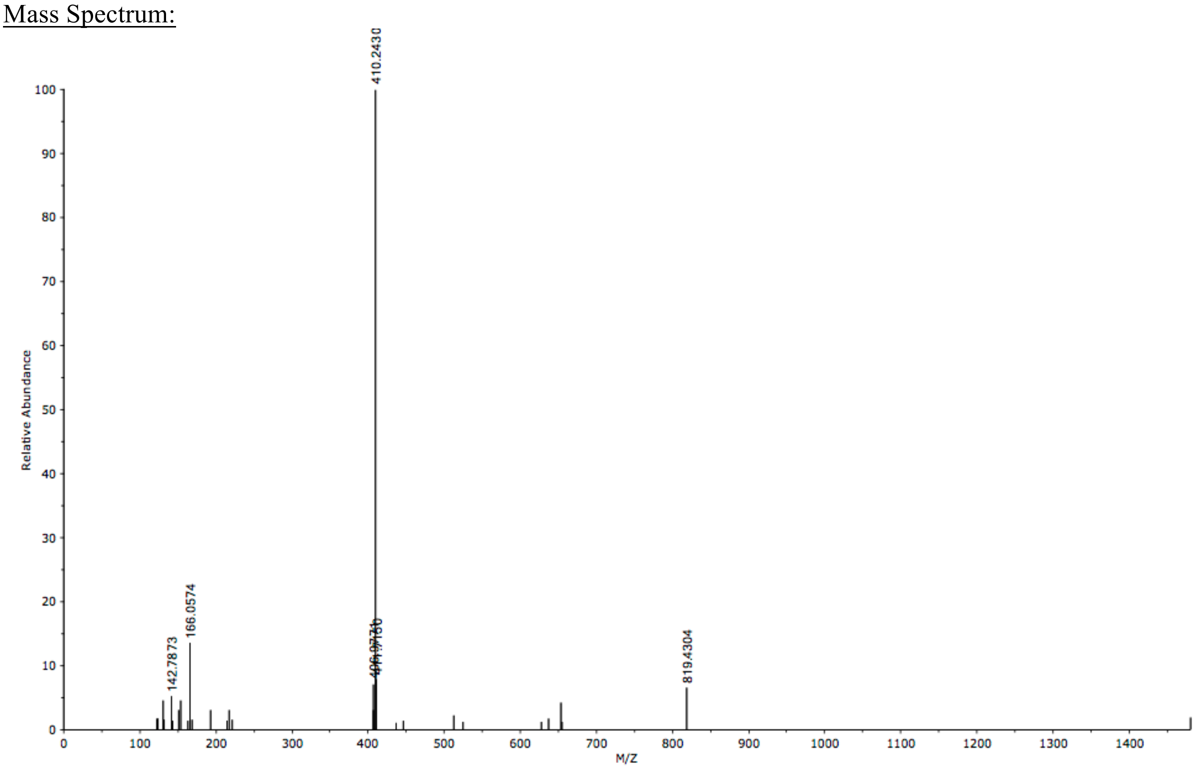

High resolution mass spectrum:

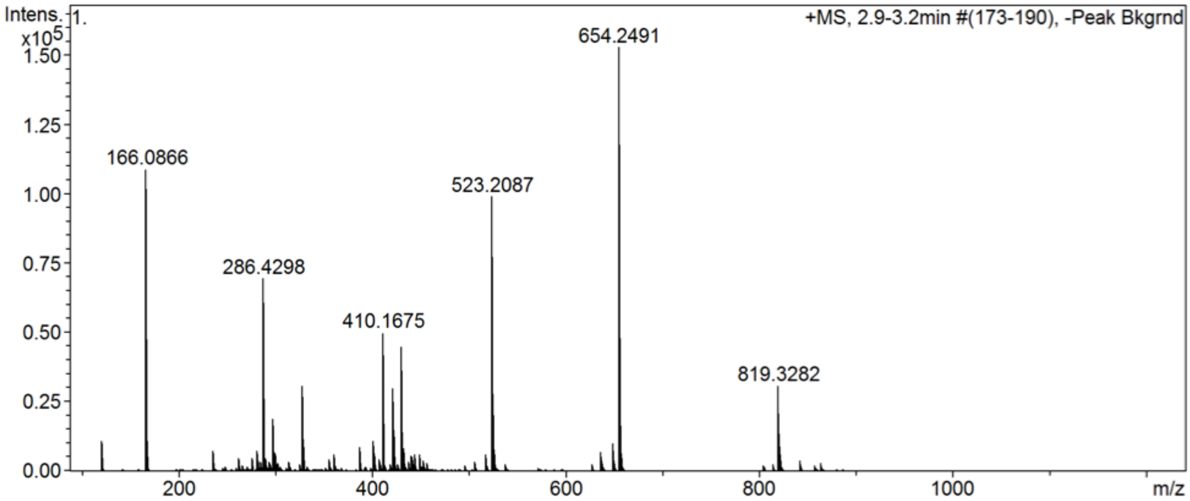

^{1}H NMR:

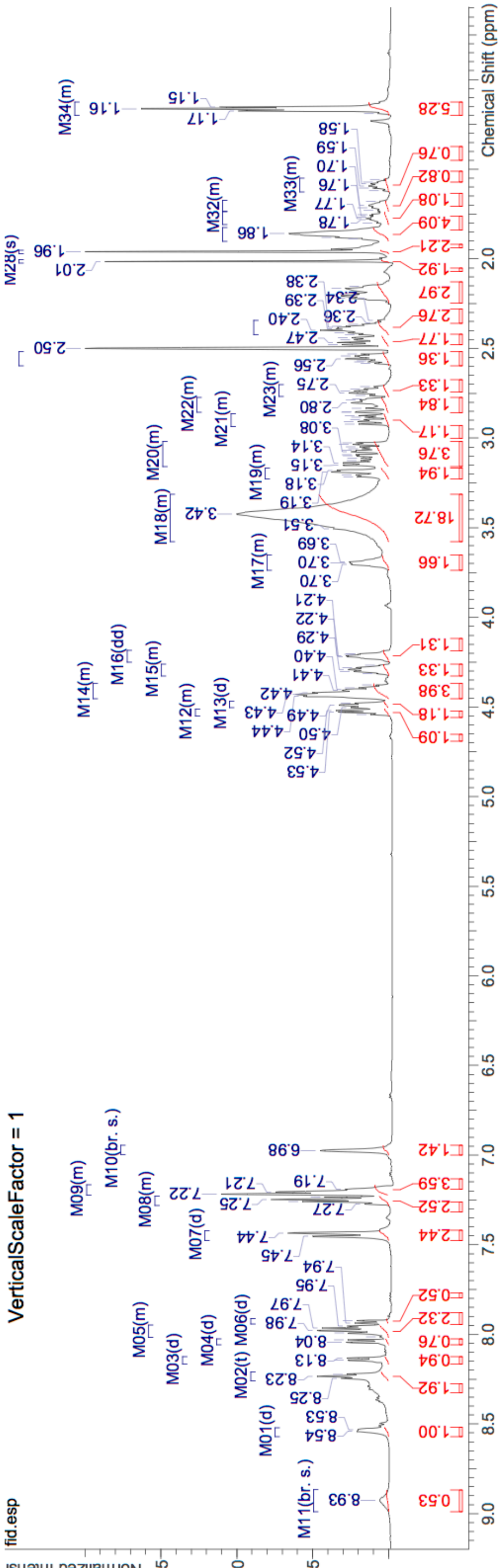
fid.esp
VerticalScaleFactor = 1
Normalized Intensi
Chemical Shift (ppm)

CMNHAPA

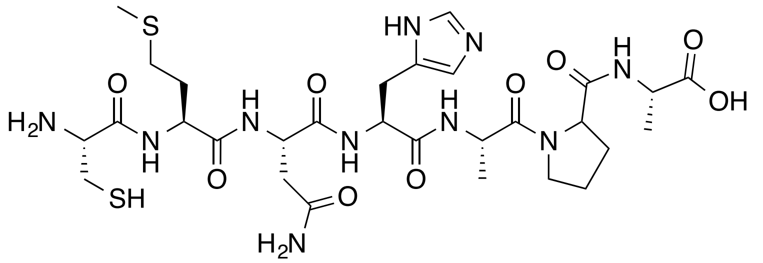

The peptide was synthesised using standard solid phase peptide synthesis techniques, with Fmoc-protected amino acids, yielding 56 mg (30%) of the product as a white solid. ^{1}H NMR (600 MHz, DMSO-d_6) δ ppm 9.45 (1 H, s, His-NH$_{side\text{-}chain}$) 9.14 (1 H, d, *J*=7.81 Hz, Asn-NH) 8.82 (2 H, d, *J*=7.81 Hz, His-NH) 8.71 - 8.78 (3 H, m, Ala-NH$_2$) 8.59 (1 H, d, *J*=7.81 Hz, Ala-NH$_1$) 8.55 (1 H, d, *J*=7.81 Hz, Met-NH) 7.94 (1 H, br. s., Asn-NH$_{side\text{-}chain}$) 7.87 (1 H, s, His-γH) 7.48 (1 H, br. s. Asn-NH$_{side\text{-}chain}$) 4.81 - 5.04 (8 H, m, Ala$_1$-α, Met-αH, Asn-αH, His-αH) 4.66 (2 H, t, *J*=7.81 Hz, Ala$_2$-αH) 4.53 (2 H, br. s., Cys-αH) 4.05 - 4.12 (1 H, m, Pro-αH) 3.99 - 4.04 (1 H, m, Pro-αH) 3.52 - 3.59 (1 H, m, Met-βH) 3.42 - 3.49 (3 H, m, Cys-βH, Met-βH) 2.88 - 3.02 (8 H, m, Pro-γH, Met-γH, His-βH) 2.50 - 2.52 (5 H, m, Asn-βH) 2.32 - 2.46 (6 H, m, Pro-βH, Asn-βH) 2.22 - 2.30 (2 H, m, Pro-γH) 1.68 - 1.77 (9 H, m, Met-δH, Ala$_1$-βH, Ala$_2$-βH); Analytical HPLC (220 nm) 15.9 min; IR (neat) 3273, 1652, 1133 cm^{-1}; MS (ESI+) *m/z* (%) 743.3 ((M + H)$^+$, 77.1), 372.5 ((M + 2H)$^+$, 100.0)); HRMS (ESI+) for $C_{26}H_{46}N_{10}O_9S_2$ (M + H)$^+$ calcd 743.2963, found 743.2953.

HPLC:

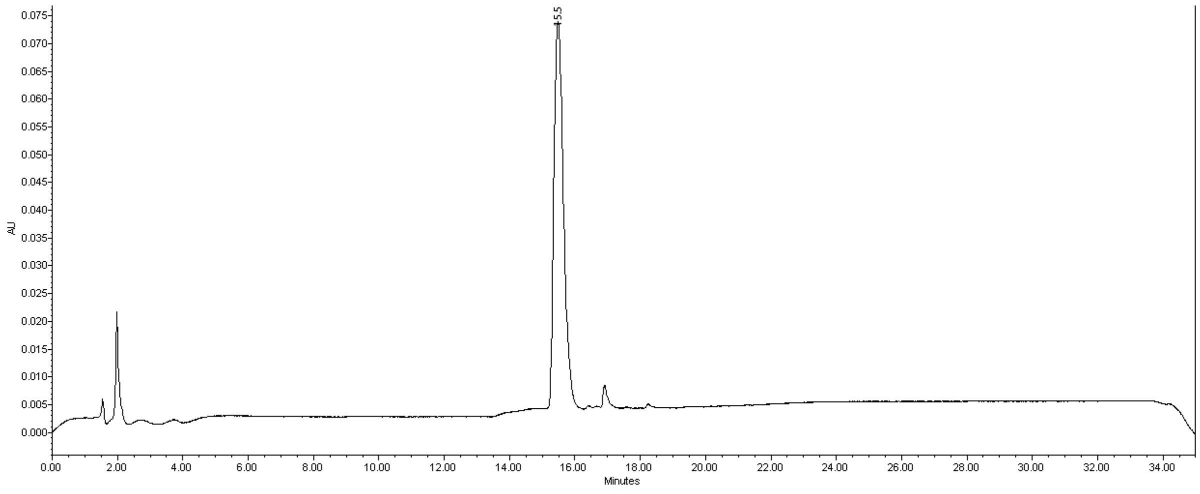

Mass Spectrum:

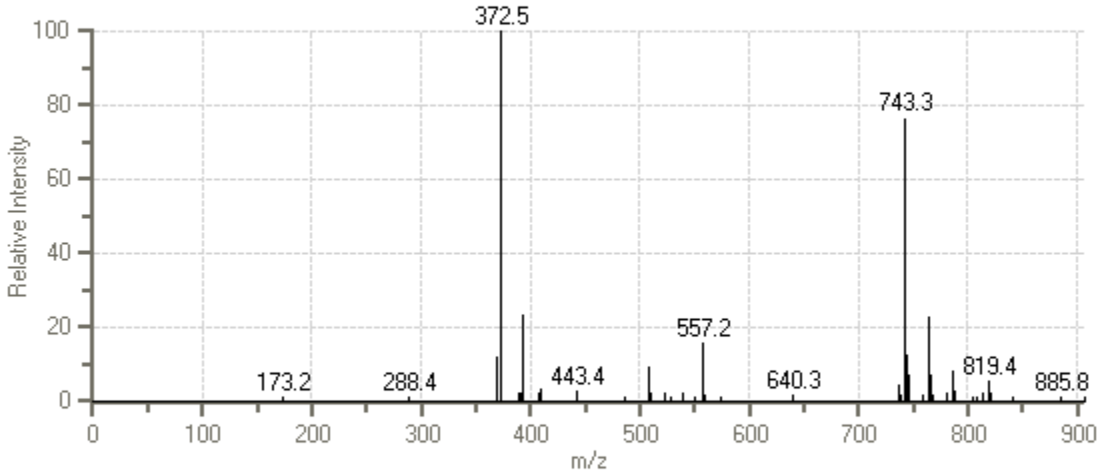

High resolution mass spectrum:

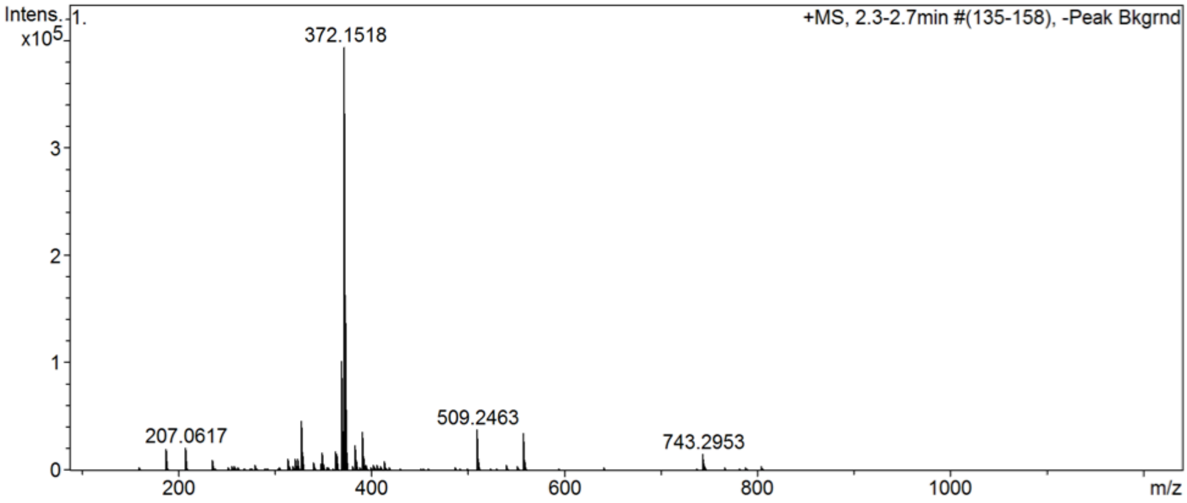

^{1}H NMR:

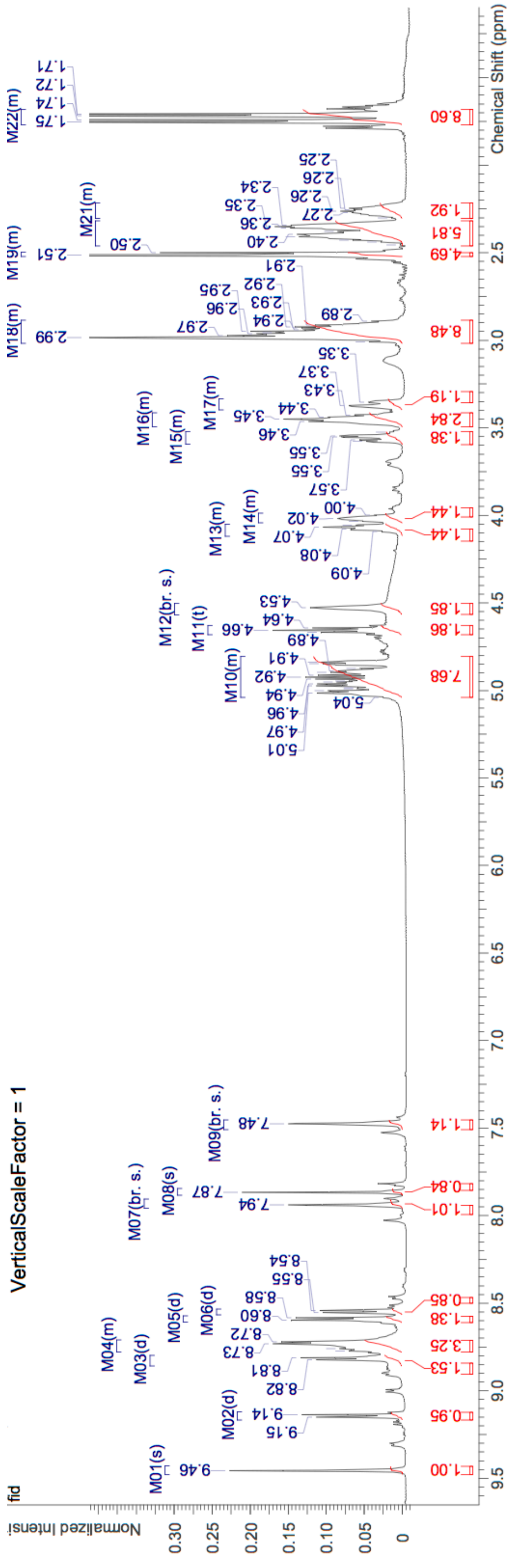
VerticalScaleFactor = 1
Chemical Shift (ppm)
Normalized Intensi

CLR(D-F)T

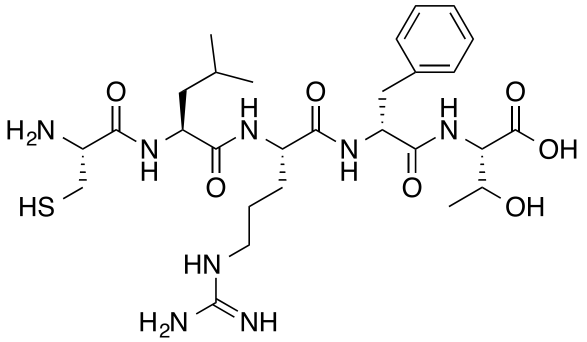

The peptide was synthesised using standard solid phase peptide synthesis techniques, with Fmoc-protected amino acids, yielding 52 mg (33%) of the product as a white solid. ^{1}H NMR (600 MHz, DMSO-d_6) δ ppm 8.49 (1 H, d, *J*=7.81 Hz, Leu-NH) 8.29 (1 H, d, *J*=9.77 Hz, Thr-NH) 8.15 (3 H, d, *J*=7.81 Hz, Phe-NH) 8.05 - 8.09 (1 H, m, Arg-NH) 7.40 (1 H, t, *J*=5.86 Hz, Arg-$NH_{side-chain}$) 7.25 - 7.30 (2 H, m, Phe-ArH) 7.19 - 7.25 (2 H, m, Phe-ArH) 7.13 - 7.19 (1 H, m, Phe-ArH) 4.96 (1 H, br. s., Cys-SH) 4.72 (1 H, td, *J*=9.77, 3.91 Hz, Thr-αH) 4.24 - 4.34 (1 H, m, Leu-αH) 4.11 - 4.23 (2 H, m, Arg-αH, Phe-αH) 4.02 (1 H, br. s., Cys-αH) 3.35 (7 H, br. s. Phe-βH) 3.13 (2 H, d, *J*=9.77 Hz, Thr-βH) 2.83 - 3.01 (3 H, m, Cys-βH, Thr-βH) 2.66 - 2.76 (2 H, m, Arg-δH) 1.59 - 1.70 (1 H, m, Leu-βH) 1.32 - 1.50 (3 H, m, Leu- βH, Leu-γH, Arg-βH) 1.22 - 1.30 (1 H, m- Arg-γH) 1.08 - 1.18 (1 H, m, Arg-γH) 1.00 (3 H, d, *J*=5.86 Hz, Thr-γH) 0.82 - 0.91 (6 H, m, Leu-δH); Analytical HPLC (220 nm) 17.7 min; IR (neat) 3278, 1643, 1133 cm^{-1}; MS (ESI+) *m/z* (%) 639.3 ((M + H)$^+$, 65.8), 320.5 ((M + 2H)$^+$, 100.0)); HRMS (ESI+) for $C_{28}H_{46}N_8O_7S$ (M + H)$^+$ calcd 639.3283, found 639.3281.

HPLC:

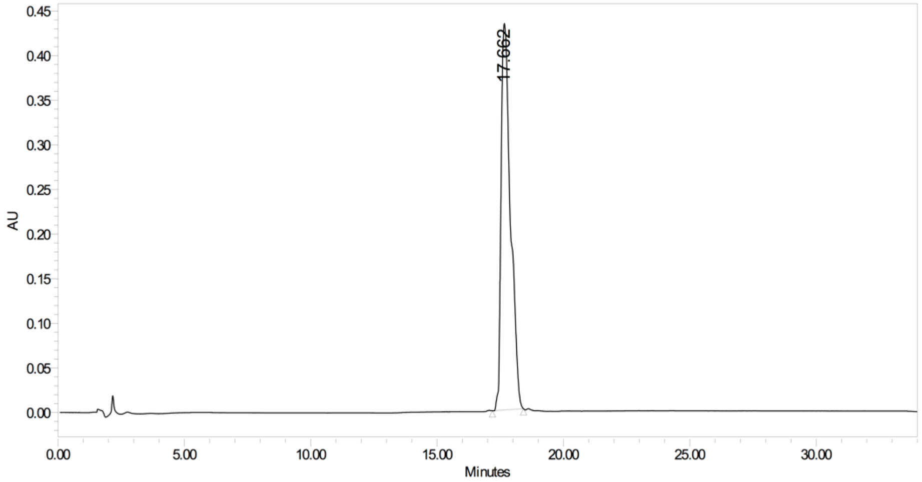

Mass Spectrum:

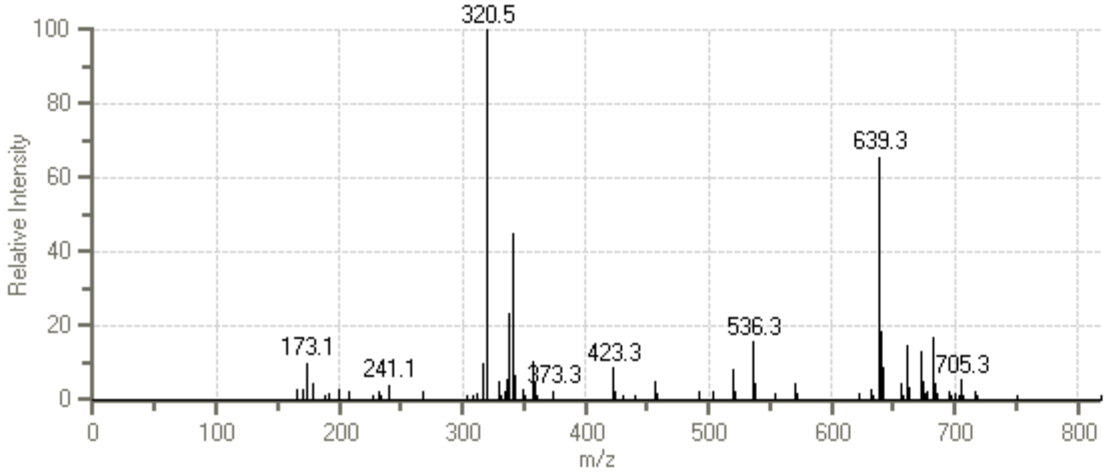

High resolution mass spectrum:

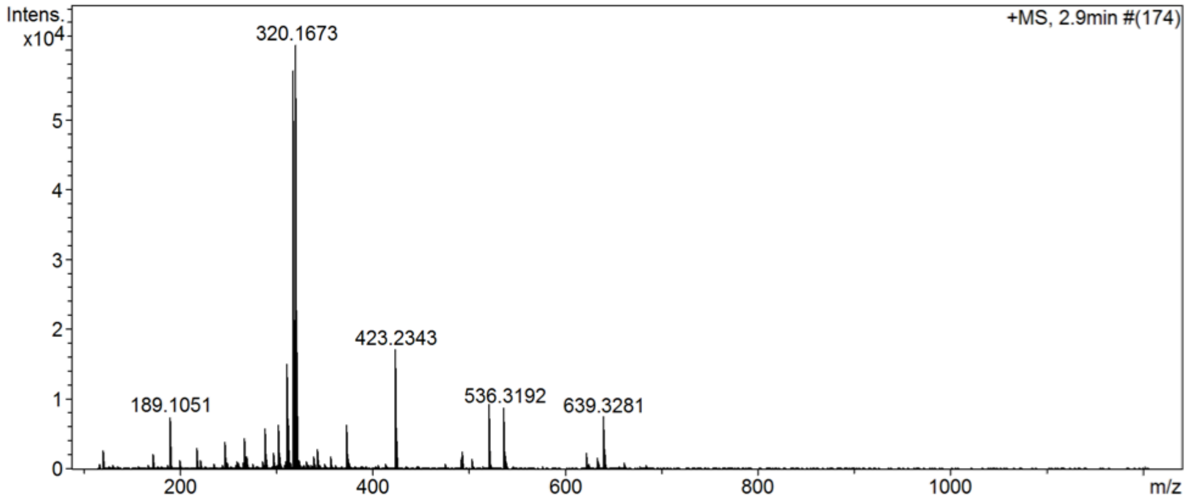

^{1}H NMR:

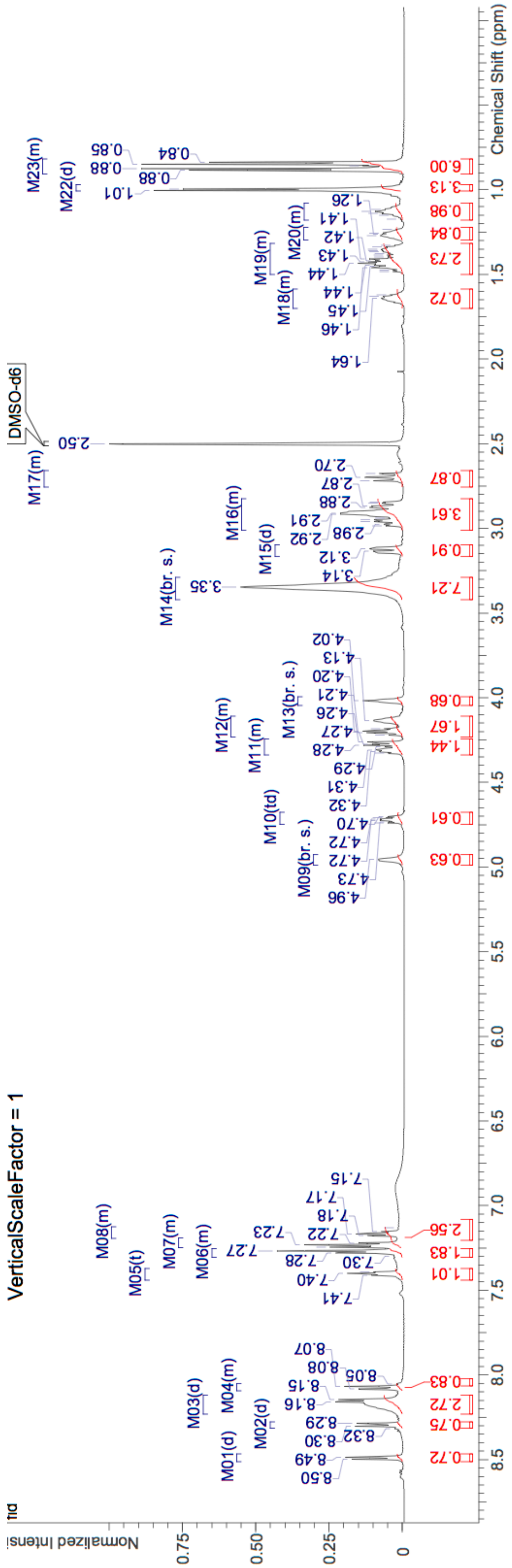
VerticalScaleFactor = 1
DMSO-d6
Chemical Shift (ppm)
Normalized Intensity

CLR(hPhe)T

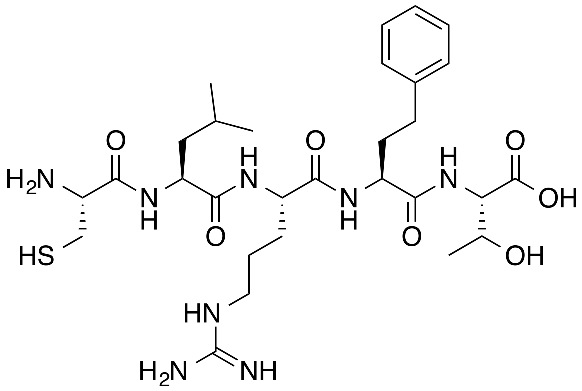

The peptide was synthesised using standard solid phase peptide synthesis techniques, with Fmoc-protected amino acids, yielding 63 mg (37%) of the product as a white solid. ^{1}H NMR (600 MHz, DMSO-d_6) δ ppm 8.54 (1 H, d, J=7.81 Hz, Leu-NH) 8.30 (1 H, d, J=7.81 Hz, Phe-NH) 8.11 (2 H, d, J=7.81 Hz, Arg-NH) 7.82 - 7.89 (1 H, m, Thr-NH) 7.58 (1 H, br. s., Arg-$NH_{side\text{-}chain}$) 7.22 – 7.30 (2 H, m, Phe-ArH) 7.11 – 7.22 (3 H, m, Phe-ArH) 4.87 – 5.06 (1 H, m, Cys-SH, Thr-OH) 4.29 – 4.46 (3 H, m, Leu-αH, Arg-αH) 4.11 – 4.24 (2 H, m, Thr-αH) 4.00 – 4.05 (1 H, m, Cys-αH) 3.25 – 3.40 (9 H, m, Thr-βH) 3.09 (2 H, d, J=5.86 Hz, Arg-δH) 2.98 – 3.03 (1 H, m, Cys-βH) 2.81 – 2.90 (1 H, m, Cys-βH) 2.52 - 2.69 (3 H, m Arg-βH) 1.90 - 2.01 (1 H, m, Arg-γH) 1.77 - 1.87 (1 H, m, Arg-γH) 1.60 - 1.74 (2 H, m, Leu-βH,) 1.44 - 1.57 (6 H, m, Leu-γH, Leu-βH) 1.05 (3 H, d, J=5.86 Hz, Thr-γH) 0.84 - 0.88 (6 H, m, Leu-δH); Analytical HPLC (220 nm) 18.2 min; IR (neat) 3272, 1631, 1134 cm^{-1}; MS (ESI+) *m/z* (%) 653.4 ((M + H)$^+$, 70.8), 327.5 ((M + 2H)$^+$, 100.0)); HRMS (ESI+) for $C_{29}H_{48}N_8O_7S$ (M + H)$^+$ calcd 653.3439, found 653.3430.

HPLC:

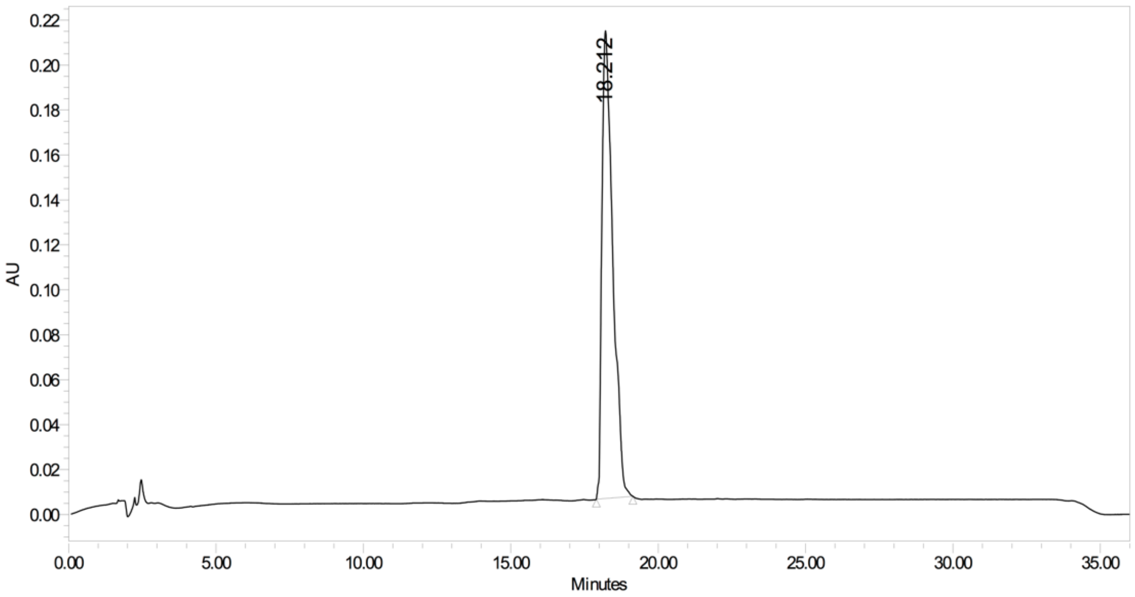

Mass Spectrum:

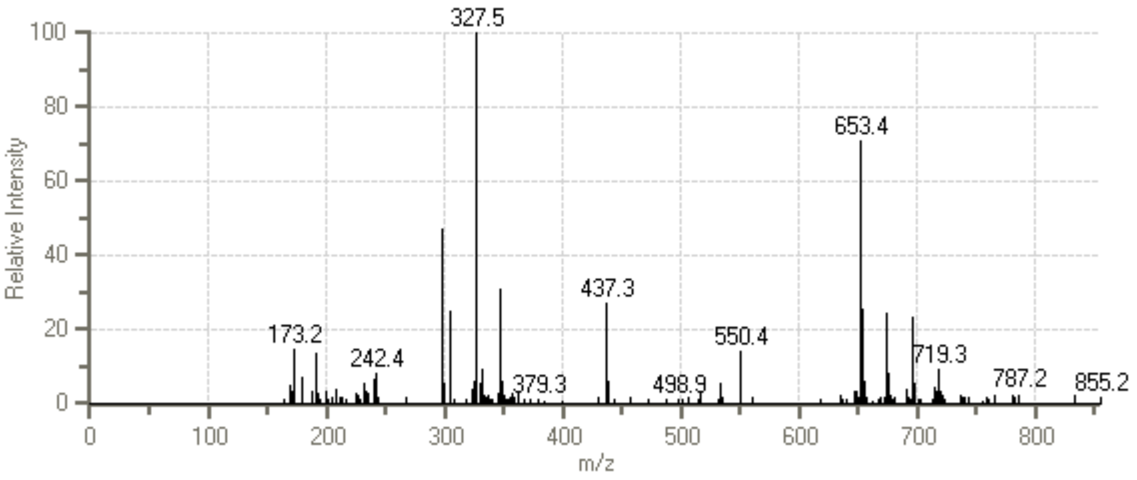

High resolution mass spectrum:

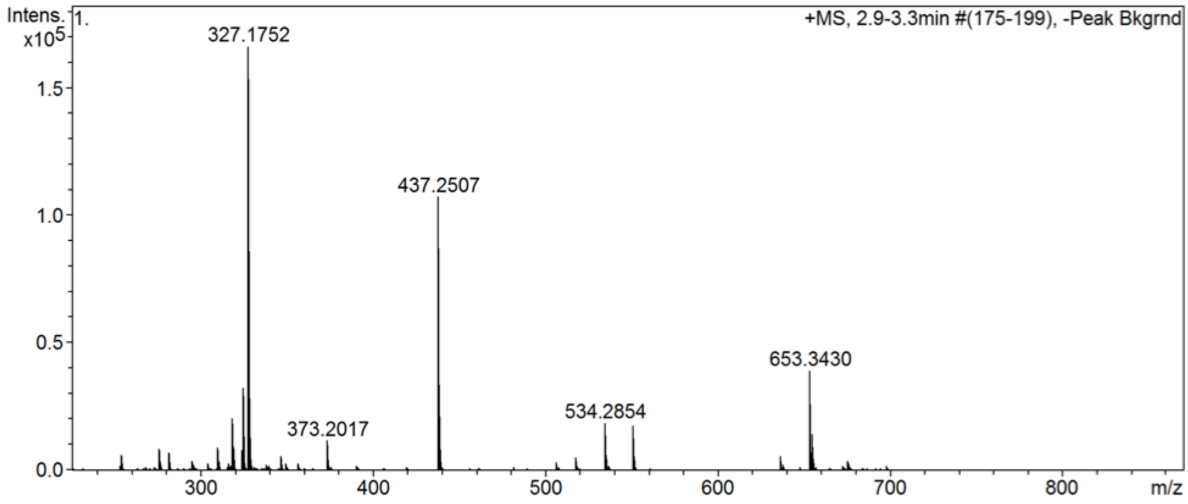

^{1}H NMR:

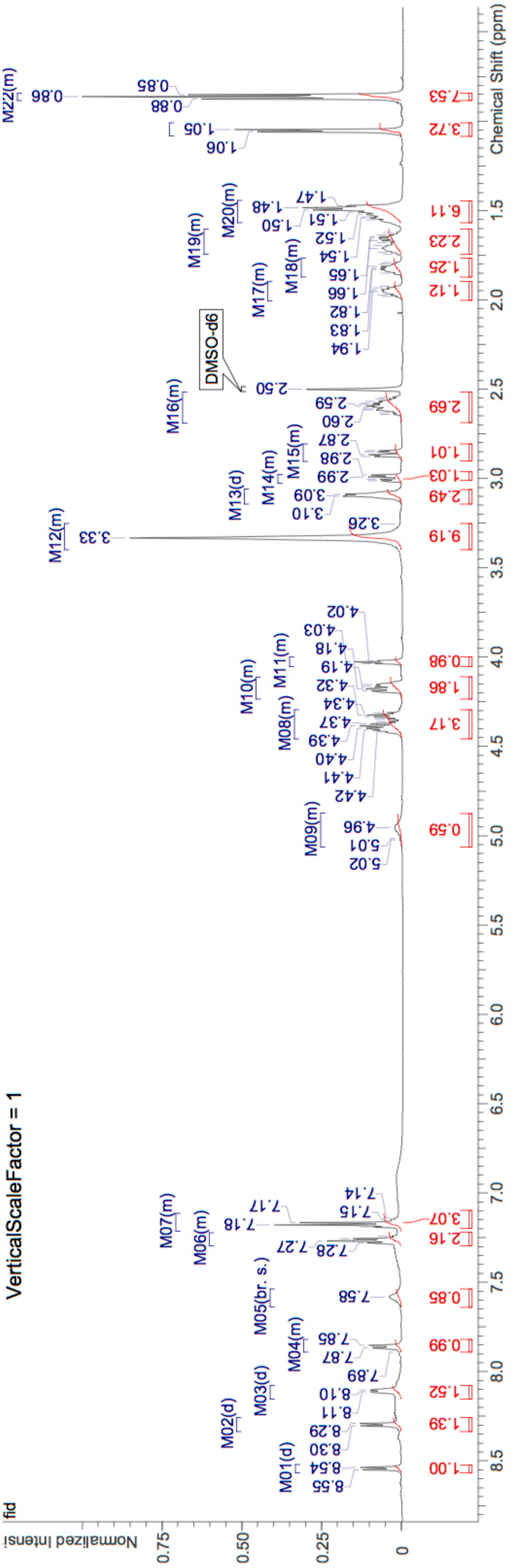
VerticalScaleFactor = 1
DMSO-d6
Chemical Shift (ppm)
Normalized Intensi
fid

CLR(Phg)T

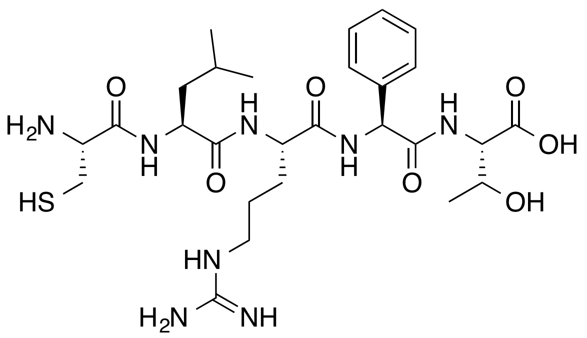

The peptide was synthesised using standard solid phase peptide synthesis techniques, with Fmoc-protected amino acids, yielding 55 mg (35%) of the product as a white solid. ^{1}H NMR (600 MHz, DMSO-d_6) δ ppm 8.54 (1 H, d, *J*=7.81 Hz, Leu-NH) 8.34 (2 H, br. s., Thr-NH, Phe-NH) 8.29 (1 H, d, *J*=7.81 Hz, Arg-NH) 7.55 - 7.74 (1 H, m, Arg-$NH_{side\text{-}chain}$) 7.44 (2 H, d, *J*=7.81 Hz, Phe-ArH) 7.25 - 7.34 (3 H, m, Phe-ArH) 5.66 (1 H, d, *J*=7.81 Hz, Cys-SH) 4.84 - 5.08 (1 H, m Thr-αH) 4.32 - 4.45 (2 H, m, Leu-αH, Arg-αH) 4.21 (1 H, d, *J*=5.86 Hz, Phe-αH) 4.09 - 4.17 (1 H, m, Cys-αH) 3.99 - 4.06 (1 H, m Thr-βH) 3.33 (8 H, br. s., Phe-βH, Phe-γH) 3.04 - 3.16 (2 H, m, Arg-δH) 2.96 - 3.03 (1 H, m, Cys-βH) 2.81 - 2.91 (1 H, m, Cys-βH) 1.72 (1 H, br. s., Arg-βH) 1.60 - 1.69 (1 H, m, Leu-βH) 1.42 - 1.58 (5 H, m, Leu-γH, Leu-βH, Arg-γH, Arg-βH) 1.05 (3 H, d, *J*=5.86 Hz, Thr-γH) 0.85 - 0.90 (6 H, m, Leu-δH); Analytical HPLC (220 nm) 17.4 min; IR (neat) 3270, 1630, 1137 cm^{-1}; MS (ESI+) *m/z* (%) 625.4 ($(M + H)^+$, 74.4), 313.5 ($(M + 2H)^+$, 100.0)); HRMS (ESI+) for $C_{27}H_{44}N_8O_7S$ $(M + H)^+$ calcd 625.3126, found 625.3133.

HPLC:

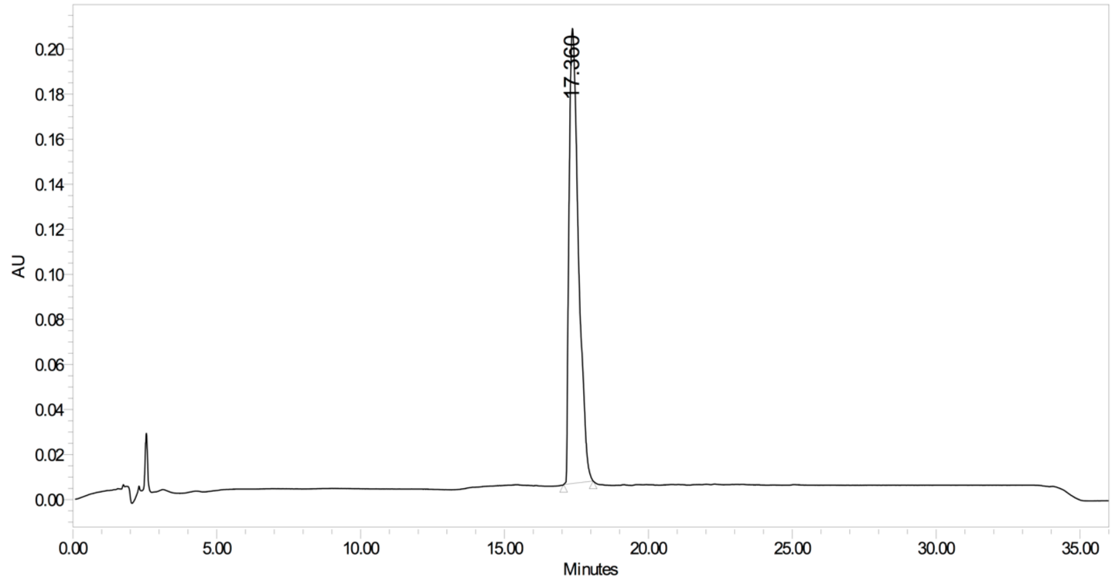

Mass Spectrum:

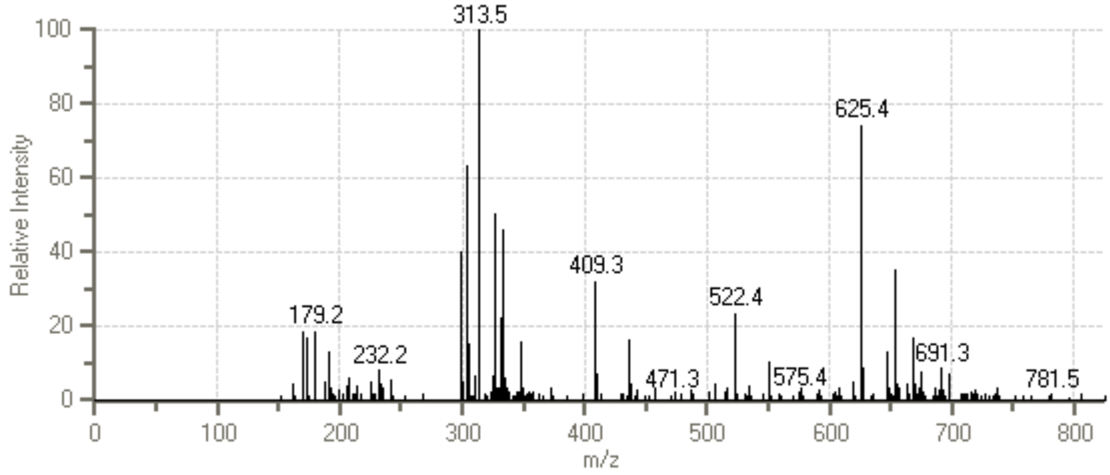

High resolution mass spectrum:

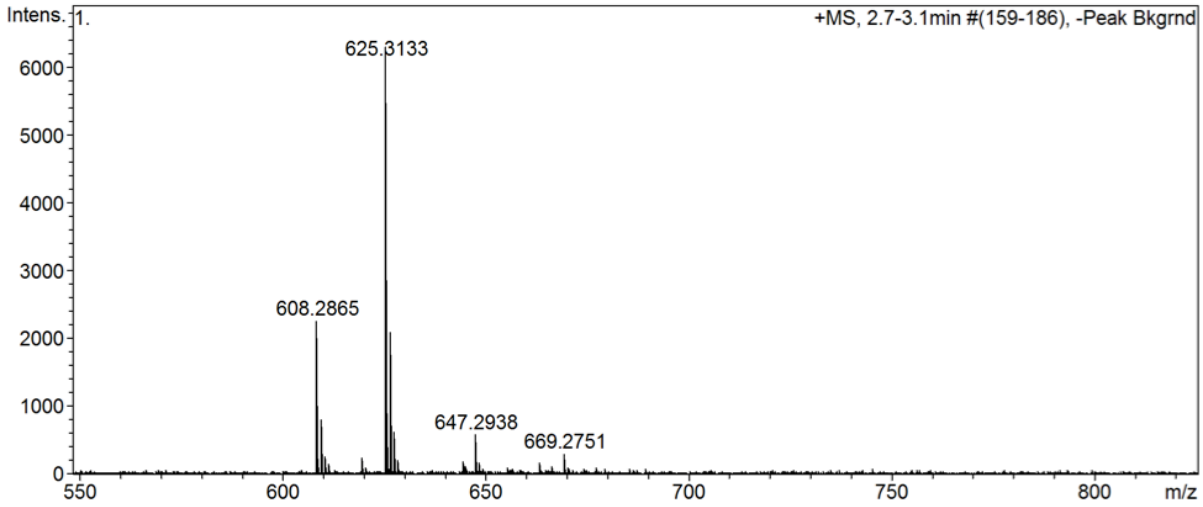

<u>^{1}H NMR:</u>

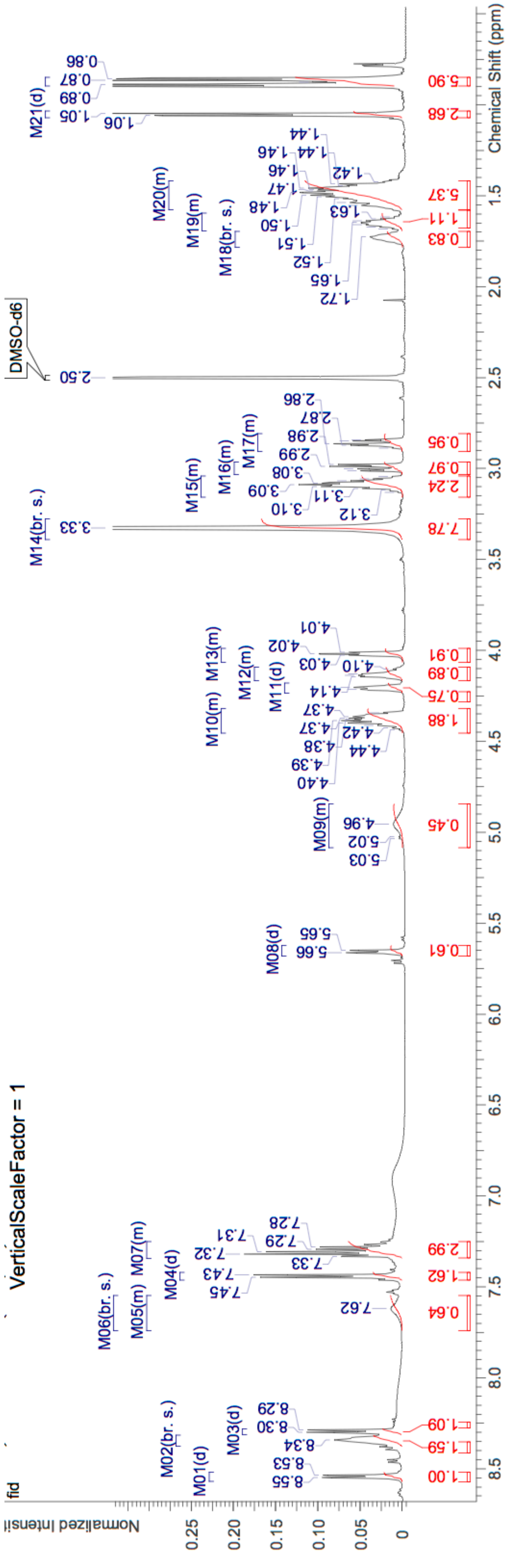
fid
VerticalScaleFactor = 1
Normalized Intensi
Chemical Shift (ppm)
DMSO-d6
2.50
M01(d) M02(br. s.) M03(d)
8.55 8.53 8.34 8.30 8.29
M04(d) M05(m) M06(br. s.) M07(m)
7.62 7.45 7.43 7.33 7.32 7.31 7.29 7.28
M08(d)
5.66 5.65
M09(m)
5.03 5.02 4.96
M10(m) M11(d) M12(m) M13(m)
4.44 4.42 4.40 4.39 4.38 4.37 4.37 4.14 4.10 4.03 4.02 4.01
M14(br. s.)
3.33
M15(m) M16(m) M17(m)
3.12 3.11 3.10 3.09 3.08 2.99 2.98 2.87 2.86
M18(br. s.) M19(m) M20(m)
1.72 1.65 1.63 1.52 1.51 1.50 1.48 1.47 1.46 1.46 1.44 1.44 1.42
M21(d)
1.06 1.05 0.89 0.87 0.86
1.00 1.59 1.09 0.64 1.62 2.99 0.61 0.45 1.88 0.75 0.89 0.91 7.78 2.24 0.97 0.95 0.83 1.11 5.37 2.68 5.90

CLR(4-Bz-F)T

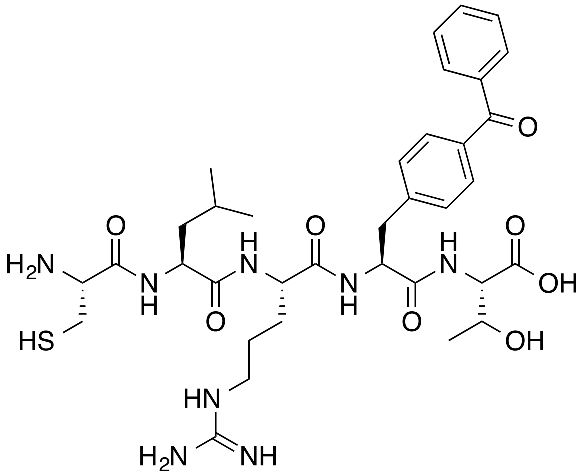

The peptide was synthesised using standard solid phase peptide synthesis techniques, with Fmoc-protected amino acids, yielding 59 mg (32%) of the product as a white solid. ^{1}H NMR (600 MHz, DMSO-d_6) δ ppm 12.64 (1 H, br. s., Thr-COOH) 8.50 (1 H, d, *J*=7.32 Hz, Leu-NH) 8.23 (2 H, d, *J*=7.32 Hz, Thr-NH, Arg-NH) 8.03 (1 H, d, *J*=9.77 Hz, Phe-NH) 7.67 - 7.72 (2 H, m, Phe-ArH) 7.63 (2 H, d, *J*=7.32 Hz, Phe-ArH) 7.56 (2 H, t, *J*=7.32 Hz, Phe-ArH) 7.47 - 7.52 (1 H, m, Arg-$NH_{side\text{-}chain}$) 7.45 (2 H, d, *J*=7.32 Hz, Phe-ArH) 4.89 - 5.11 (1 H, m., Cys-SH) 4.76 - 4.81 (1 H, m, Phe-αH) 4.30 - 4.35 (1 H, m, Leu-αH) 4.21 - 4.30 (2 H, m, Thr-αH, Arg-αH) 4.18 (1 H, d, *J*=4.88 Hz, Cys-αH) 4.02 (1 H, br. s.,Thr-OH) 3.18 (1 H, d, *J*=9.77 Hz, Phe-βH) 3.02 - 3.08 (3 H, m, Thr-βH, Arg-δH, Cys-βH) 2.89 - 3.01 (2 H, m, Arg-δH, Phe-βH) 2.79 - 2.88 (1 H, m, Arg-βH) 2.54 - 2.65 (1 H, m, Arg-βH) 1.55 - 1.69 (2 H, m, Leu-βH) 1.34 - 1.52 (6 H, m, Leu-γH, Arg-γH) 1.04 - 1.08 (4 H, m, Thr-γH) 0.77 - 0.81 (5 H, m, Leu-δH); Analytical HPLC (220 nm) 18.9 min; IR (neat) 3277, 1639, 1135 cm^{-1}; MS (ESI+) *m/z* (%) 745.4 ((M + H)$^+$, 45.5), 372.5 ((M + 2H)$^+$, 54.3); HRMS (ESI+) for $C_{35}H_{50}N_8O_8S$ (M + 2H)$^+$ calcd 372.1809, found 372.1818.

HPLC:

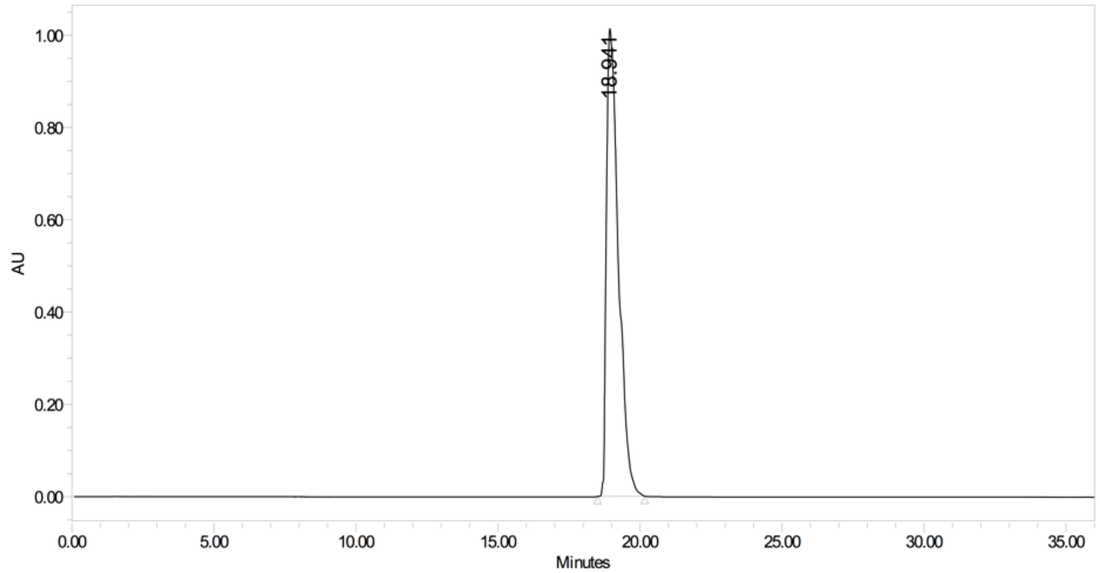

Mass Spectrum:

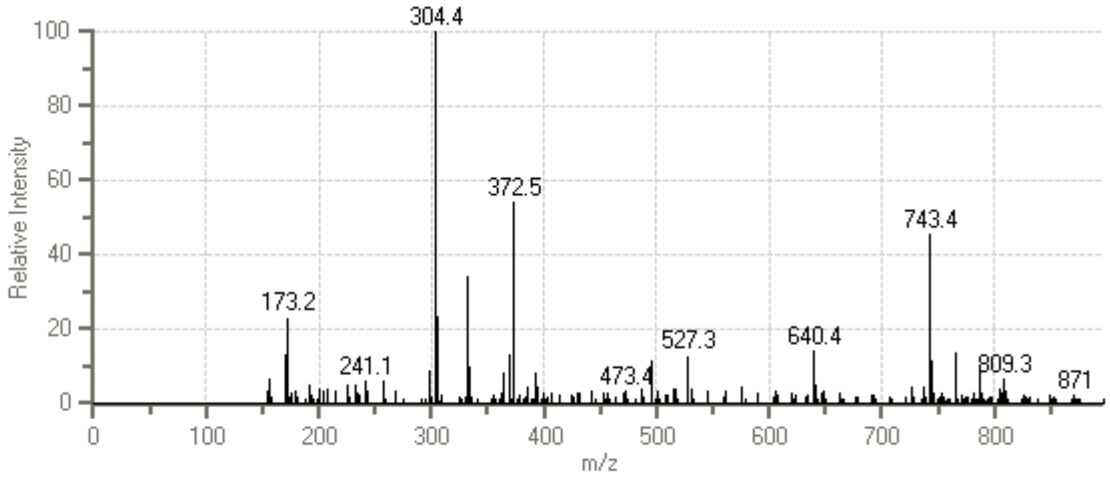

High resolution mass spectrum:

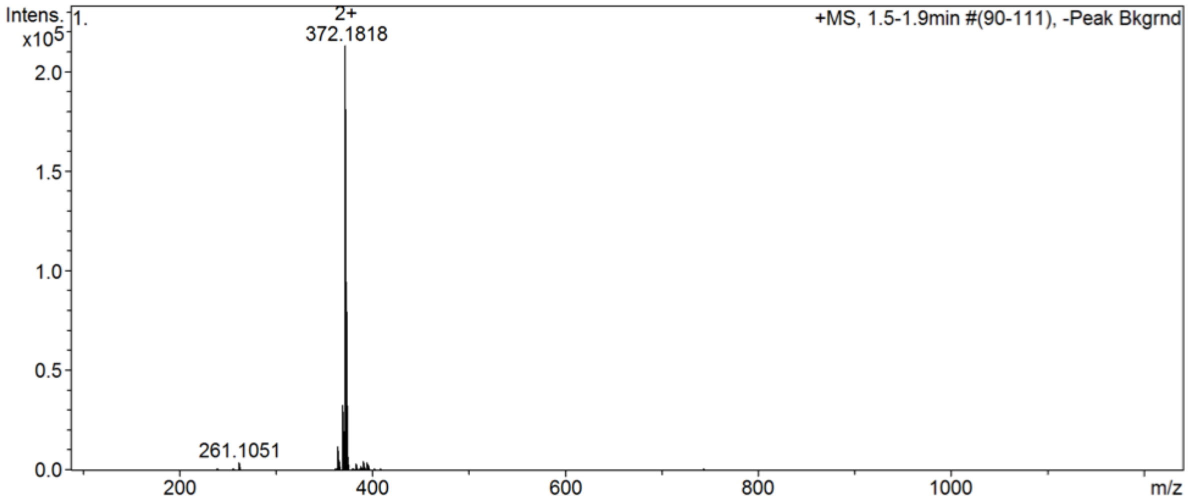

^{1}H NMR:

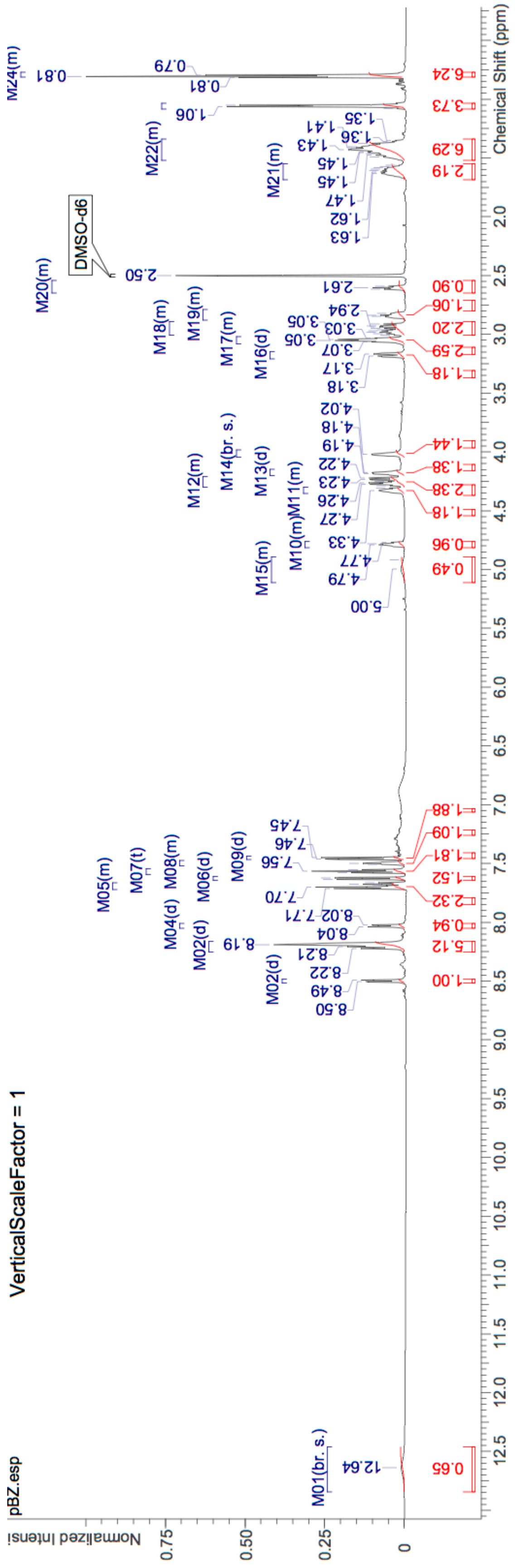
pBZ.esp
VerticalScaleFactor = 1
DMSO-d6
Chemical Shift (ppm)
Normalized Intensi
M01(br. s.)
12.64
M24(m)
M22(m)
M21(m)
M20(m)
2.50
0.81
0.79
1.06

CLRYT

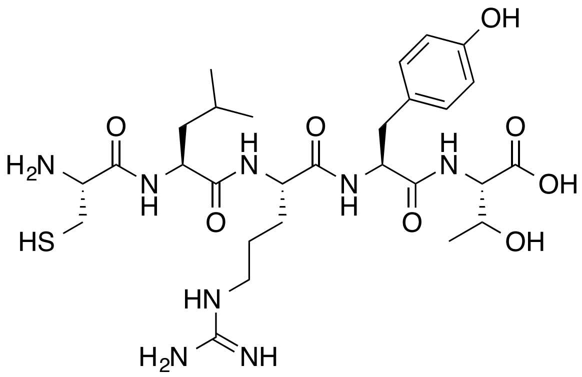

The peptide was synthesised using standard solid phase peptide synthesis techniques, with Fmoc-protected amino acids, yielding 63 mg (37%) of the product as a white solid. ^{1}H NMR (600 MHz, DMSO-d_6) δ ppm 9.16 (1 H, br. s., Tyr-OH) 8.52 (1 H, d, *J*=7.81 Hz, Leu-NH) 8.17 (1 H, d, *J*=7.81 Hz, Thr-NH) 8.04 (1 H, d, *J*=7.81 Hz, Tyr-NH) 7.86 (1 H, d, *J*=9.77 Hz, Arg-NH) 7.55 (1 H, br. s., Arg-$NH_{side-chain}$) 7.01 - 7.06 (2 H, m, Tyr-ArH) 6.61 (2 H, d, *J*=7.81 Hz, Tyr-ArH) 4.94 (1 H, br. s., Cys-SH) 4.55 - 4.64 (1 H, m, Arg-αH) 4.31 - 4.38 (1 H, m, Leu-αH) 4.24 - 4.29 (1 H, m, Tyr-αH) 4.19 - 4.23 (1 H, m, Thr-OH) 4.16 (1 H, d, *J*=5.86 Hz, Thr-αH) 4.01 - 4.05 (1 H, m, Cys-αH) 3.28 - 3.50 (8 H, m, Solvent-H_2O) 3.02 - 3.10 (3 H, m, Thr-βH, Tyr-βH) 2.91 - 3.01 (2 H, m, Cys-βH) 2.86 (1 H, dd, *J*=13.67, 3.91 Hz, Arg-βH) 2.70 (1 H, dd, *J*=13.67, 7.81 Hz, Arg-βH) 1.57 - 1.69 (2 H, m, Leu-βH) 1.37 - 1.52 (7 H, m, Leu-γH, Arg-γH, Thr-βH) 1.04 (3 H, d, *J*=7.81 Hz, Thr-γH) 0.83 - 0.91 (4 H, m, Leu-δH); Analytical HPLC (220 nm) 18.9 min; IR (neat) 3280, 1638, 1134 cm^{-1}; MS (ESI+) *m/z* (%) 655.3 ($(M + H)^+$, 68.1), 328.4 ($(M + 2H)^+$, 100.0)); HRMS (ESI+) for $C_{28}H_{45}N_9O_9S$ $(M + H)^+$ calcd 655.3232, found 655.3225.

HPLC:

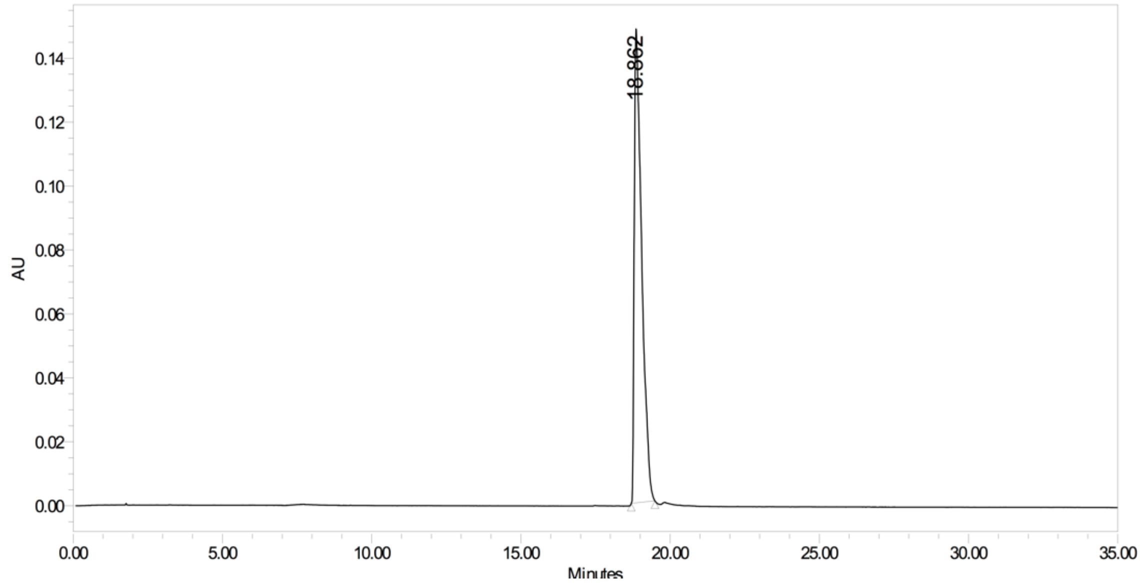

Mass Spectrum:

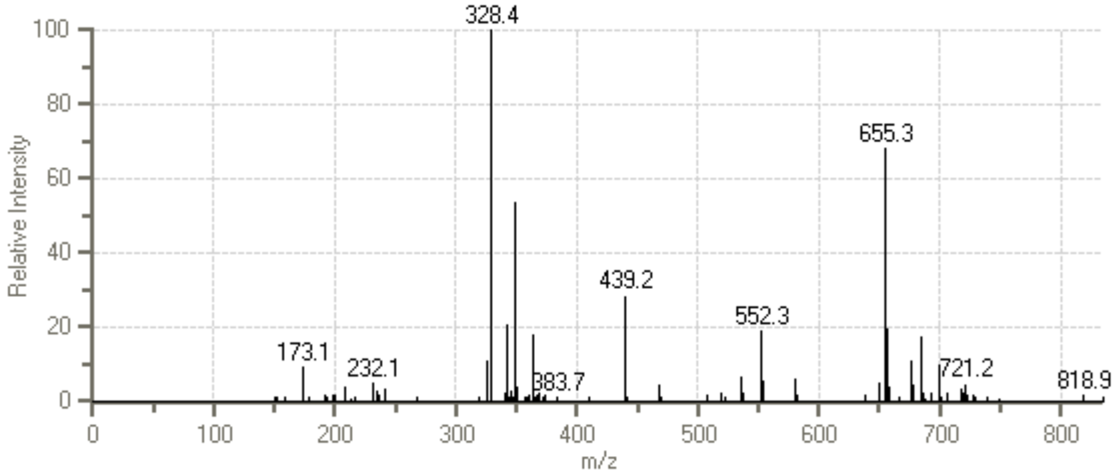

High resolution mass spectrum:

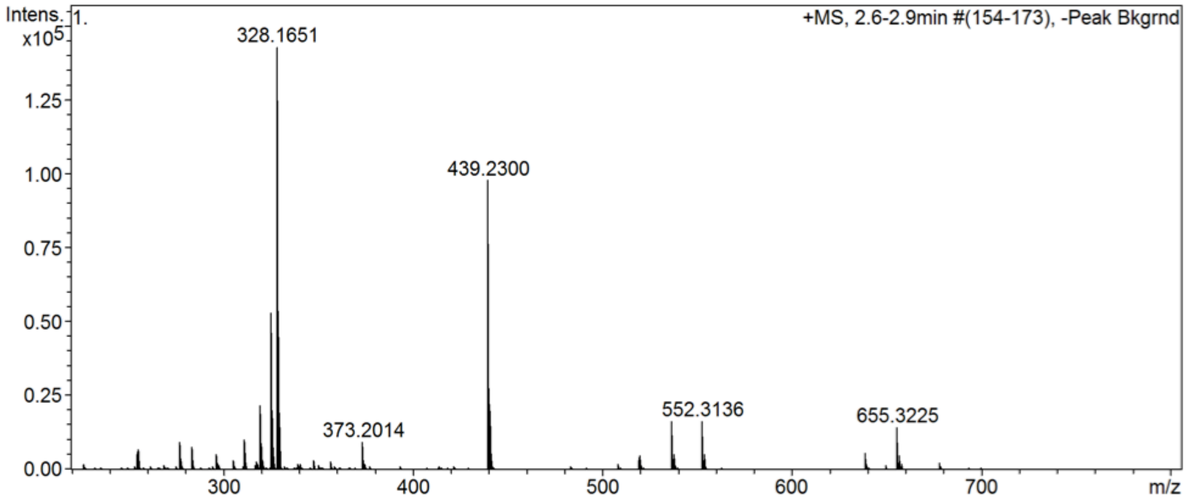

^1H NMR:

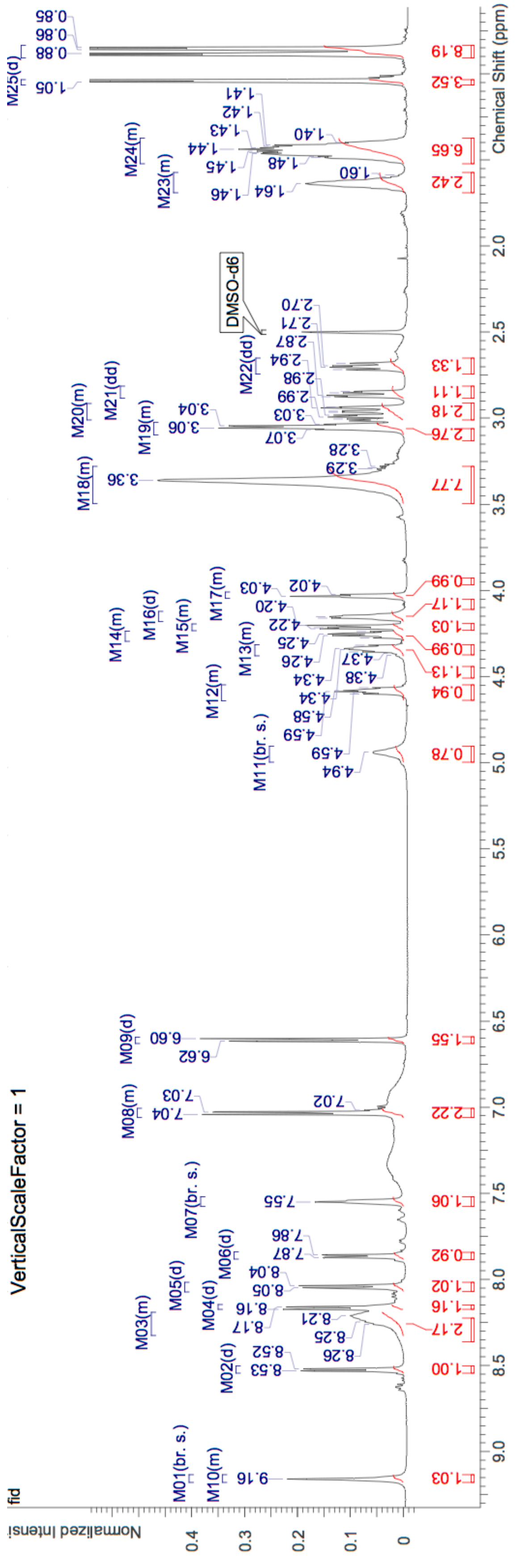
VerticalScaleFactor = 1
Chemical Shift (ppm)
Normalized Intensi
fid
DMSO-d6

CLR(4-NO_2F)T

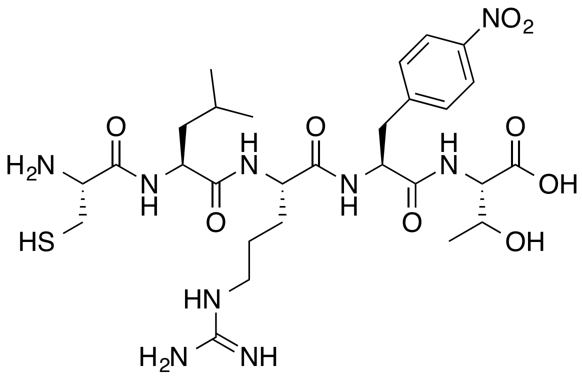

The peptide was synthesised using standard solid phase peptide synthesis techniques, with Fmoc-protected amino acids, yielding 65 mg (40%) of the product as a white solid. ^{1}H NMR (600 MHz, DMSO-d_6) δ ppm 8.51 (1H, d, *J*=7.81, Leu-NH) 8.26 (d, *J*=7.81 Hz, 1 H, Thr-NH) 8.15 (d, *J*=7.81 Hz, 1 H, Phe-NH) 8.10 (d, *J*=7.81 Hz, 2 H, Phe-ArH) 8.03 -8.07 (m, 1 H, Arg-NH) 7.56 (d, *J*=7.81 Hz, 2 H, Phe-ArH) 5.03 (br. s., 1 H, Cys-SH) 4.75 - 4.85 (m, 1 H, Arg-αH) 4.32 (t, *J*=11.72 Hz, 1 H, Leu- αH) 4.15 - 4.26 (m, 3 H, Phe-αH, Thr-α, Thr-βH) 4.03 (br. s., 1 H, Cys-αH) 3.21 (d, *J*=13.67, 3.91 Hz, 2 H, Arg-βH) 3.02 - 3.08 (m, 2 H, Phe-βH) 2.93 (dd, *J*=13.67, 9.77 Hz, 1 H, Thr-βH) 1.60 (d, *J*=5.86 Hz, 3 H, Arg-δH, Leu-βH) 1.37 - 1.51 (m, 5 H, Leu-γH, Cys-βH, Leu-βH) 1.29 - 1.36 (m, 2 H, Arg-γH) 1.05 (d, *J*=5.86 Hz, 3 H, Thr-δH) 0.82-0.85 (d, *J*=7.81 Hz, 6 H, Leu-δH); Analytical HPLC (220 nm) 19.8 min; IR (neat) 3275, 1637, 1135 cm^{-1}; MS (ESI+) *m/z* (%) 684.3 ((M + H)$^+$, 100.0); HRMS (ESI+) for $C_{28}H_{45}N_9O_9S$ (M + H)$^+$ calcd 684.3134, found 684.3133.

HPLC:

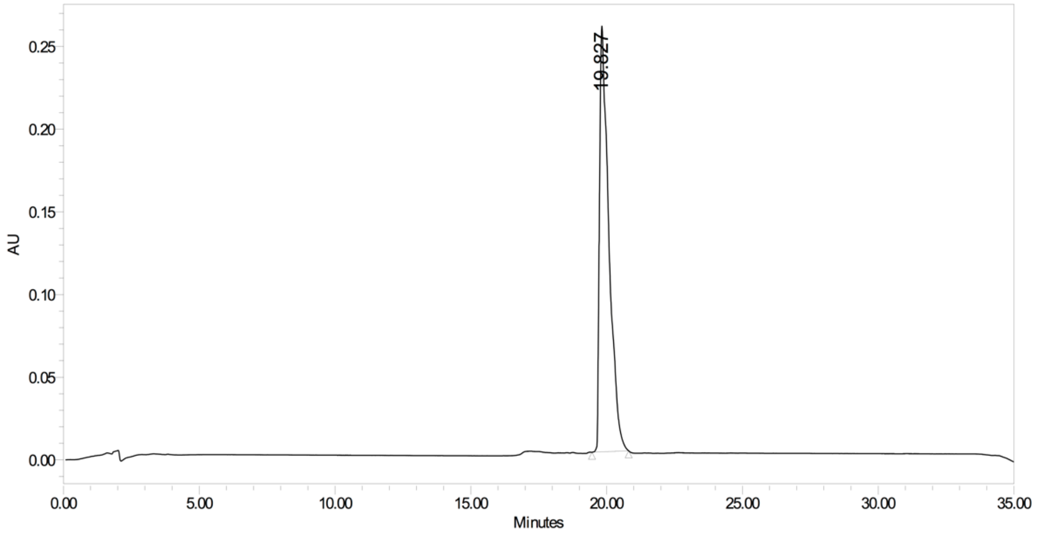

Mass Spectrum:

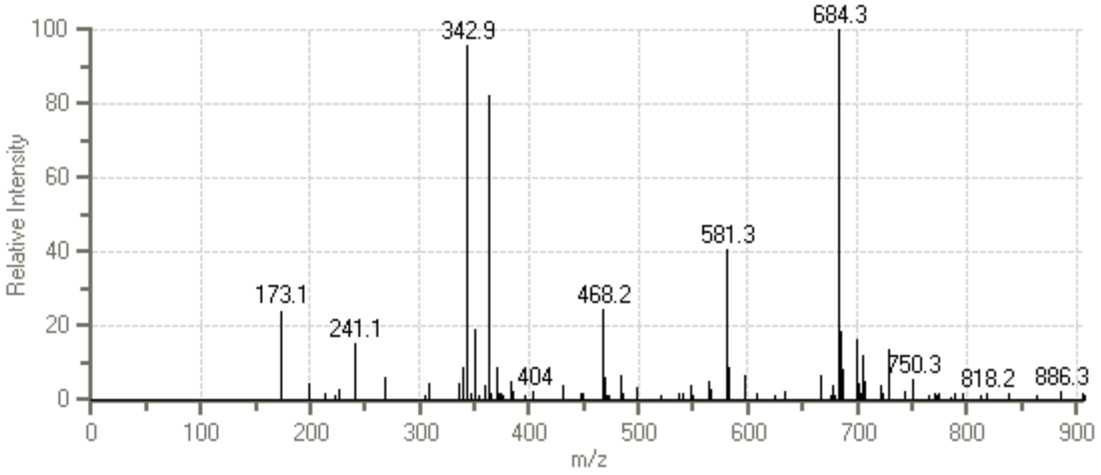

High resolution mass spectrum:

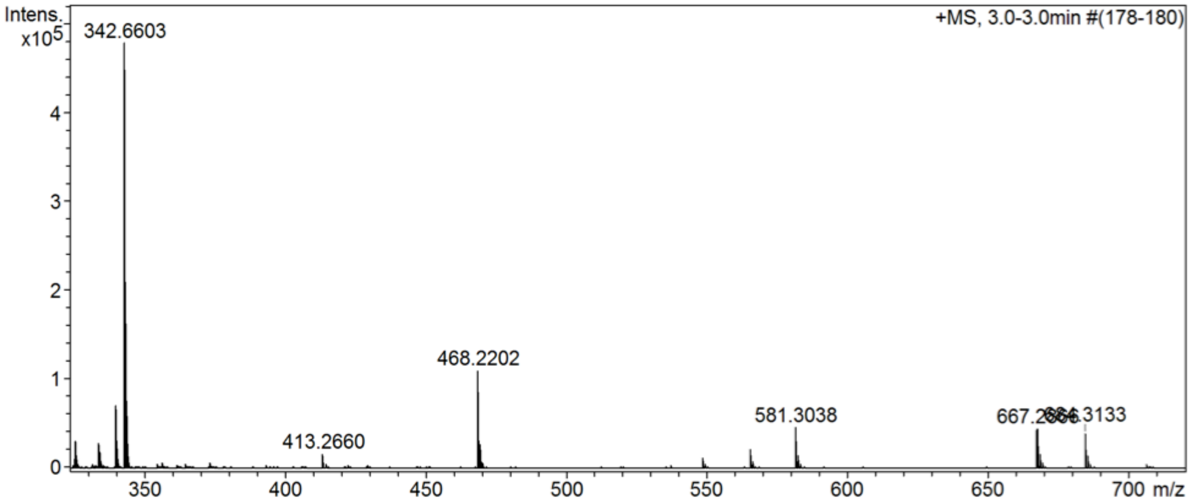

^{1}H NMR:

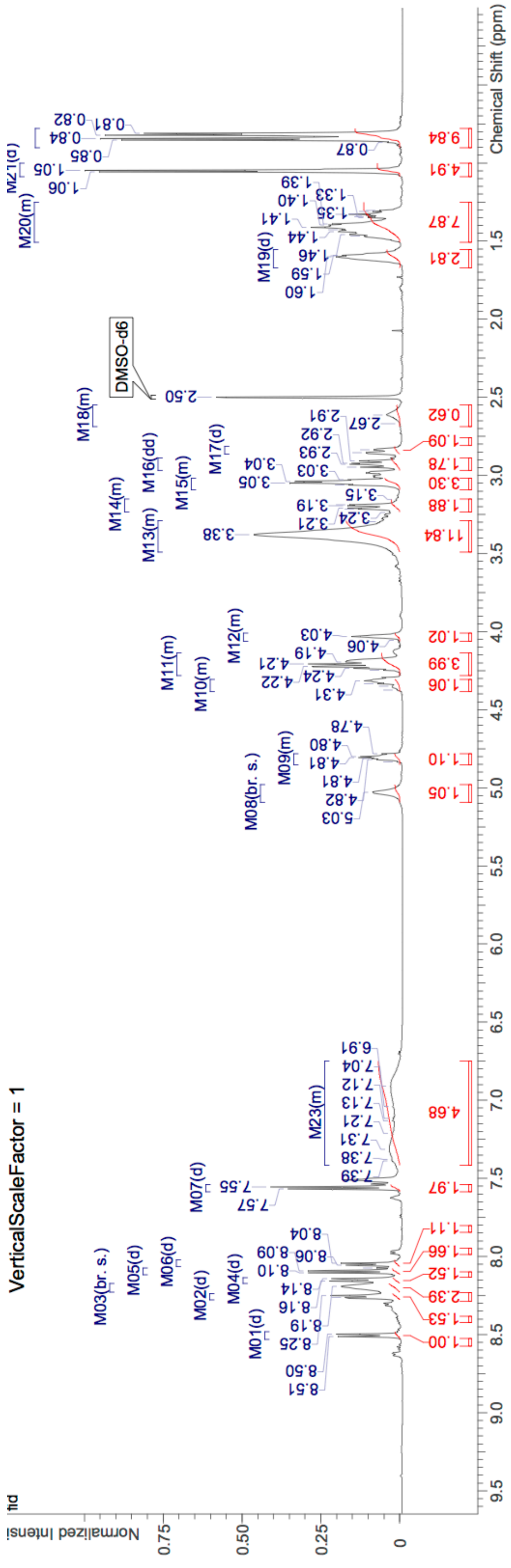

VerticalScaleFactor = 1
Normalized Intensity
Chemical Shift (ppm)
DMSO-d6

CLR(4-CN-F)T

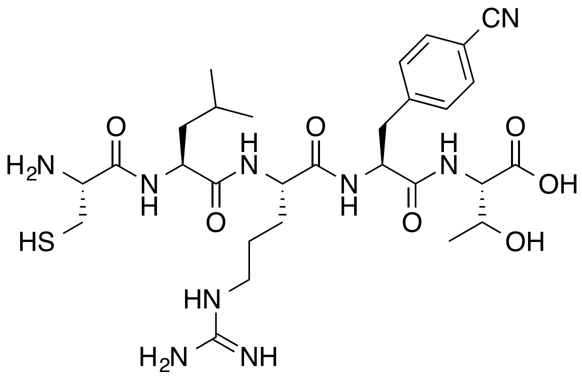

The peptide was synthesised using standard solid phase peptide synthesis techniques, with Fmoc-protected amino acids, yielding 60 mg (36%) of the product as a white solid. ^{1}H NMR (600 MHz, DMSO-d_6) δ ppm 12.65 (1 H, br. s., Thr-COOH) 8.51 (1 H, d, *J*=7.32 Hz, Leu-NH) 8.22 (1 H, d, *J*=9.77 Hz, Thr-NH) 8.19 (3 H, br. s., Arg-NH) 8.15 (1 H, d, *J*=7.32 Hz, Phe-NH) 8.02 (1 H, d, *J*=9.77 Hz, Phe-ArH) 7.68 (1 H, d, *J*=9.77 Hz, Phe-ArH) 7.48 (3 H, d, *J*=7.32 Hz, Phe-ArH, Arg-$NH_{side-chain}$) 5.02 (1 H, br. s., Cys-SH) 4.73 - 4.80 (1 H, m, Phe-αH) 4.30 - 4.37 (1 H, m, Leu-αH) 4.16 - 4.26 (3 H, m, Thr-αH, Thr-βH, Arg-αH) 3.99 - 4.09 (1 H, m, Cys-αH) 3.12 - 3.19 (1 H, m, Phe-βH) 2.97 - 3.08 (3 H, m, Cys-βH, Arg-δH) 2.82 - 2.91 (2 H, m, Arg-βH, Phe-βH) 2.58 - 2.66 (1 H, m, Arg-βH) 1.55 - 1.68 (2 H, m, Leu-βH) 1.31 - 1.50 (6 H, m, Leu-γH, Arg-γH) 1.05 (3 H, d, *J*=7.32 Hz, Thr-γH) 0.83 - 0.91 (6 H, m, Leu-δH); Analytical HPLC (220 nm) 17.6 min; IR (neat) 3278, 1642, 1134 cm^{-1}; MS (ESI+) *m/z* (%) 664.1 ($(M + H)^+$, 76.8), 333.0 ($(M + 2H)^+$, 100.0)); HRMS **(**ESI+) for $C_{29}H_{45}N_9O_7S$ $(M + 2H)^+$ calcd 332.6654, found 332.6660.

HPLC:

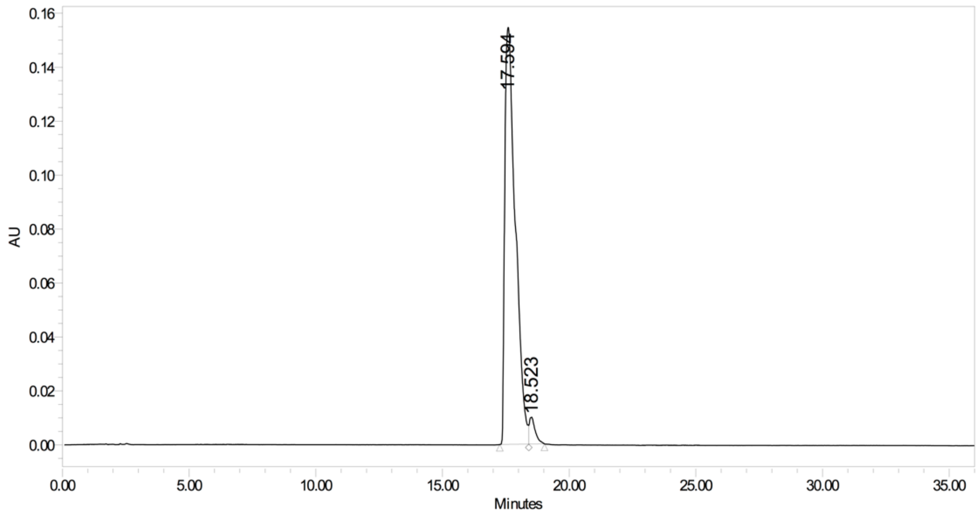

Mass Spectrum:

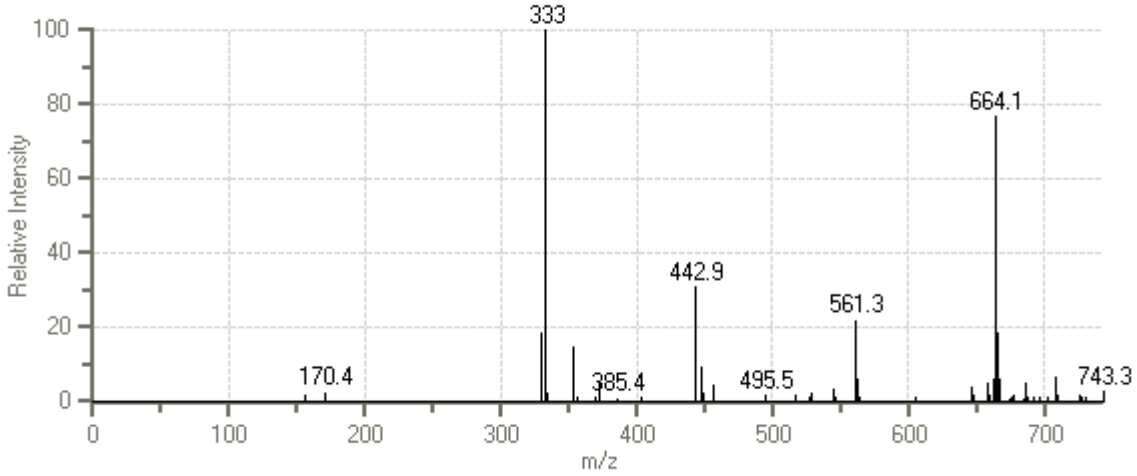

High resolution mass spectrum:

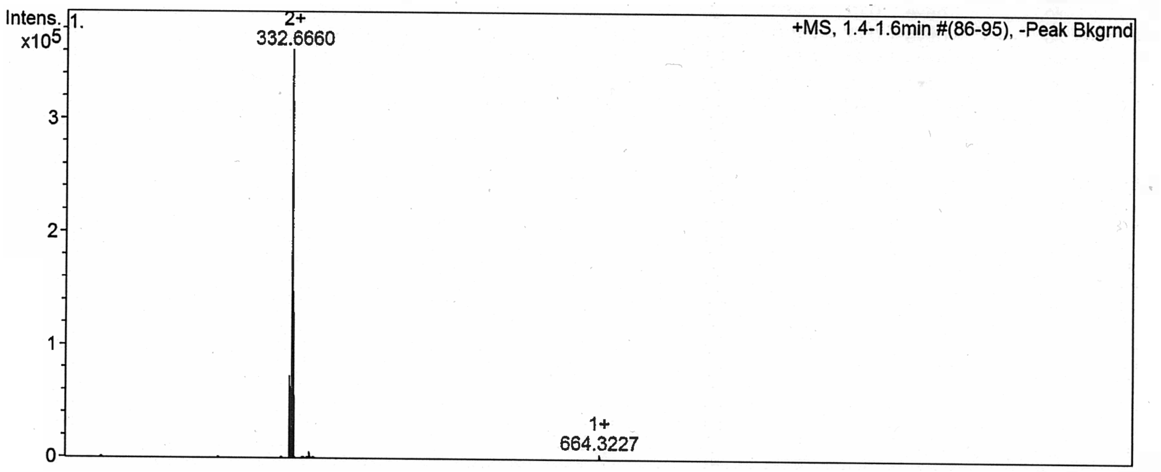

^{1}H NMR:

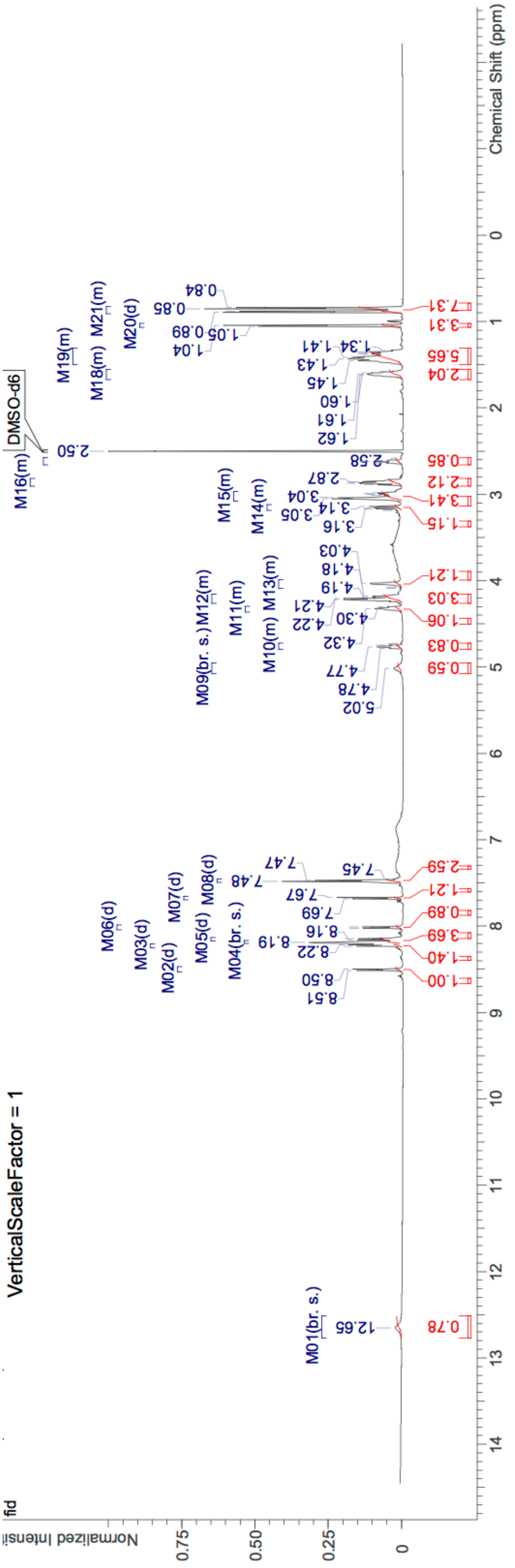

VerticalScaleFactor = 1
DMSO-d6
Chemical Shift (ppm)
Normalized Intensity

CLR(3,5-Br$_2$-Y)T

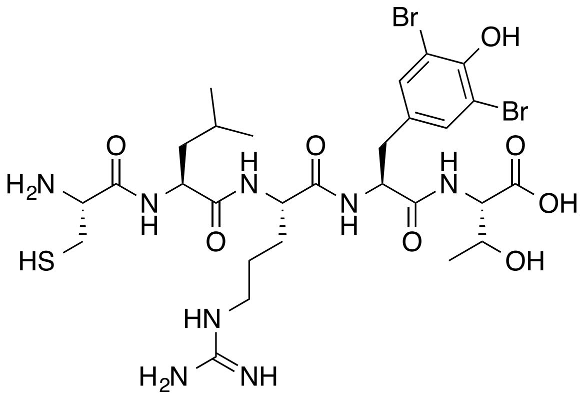

The peptide was synthesised using standard solid phase peptide synthesis techniques, with Fmoc-protected amino acids, yielding 27 mg (53%) of the product as a white solid. ^{1}H NMR (600 MHz, DMSO-d_6) δ ppm 12.64 (1 H, br. s., Thr-COOH) 9.66 (1 H, br. s. Tyr-OH) 8.50 (1 H, d, *J*=7.32 Hz, Leu-NH) 8.27 (1 H, d, *J*=7.32 Hz, Thr-NH) 8.19 (3 H, d, *J*=7.32 Hz, Tyr-NH) 7.84 (1 H, d, *J*=7.32 Hz, Arg-NH) 7.48 (2 H, s, Tyr-ArH) 5.02 (1 H, br. s., Cys-SH) 4.64 (1 H, br. s. Arg-αH) 4.31 - 4.38 (1 H, m, Leu-αH) 4.15 - 4.25 (3 H, m, Thr-αH, Tyr-αH) 4.02 (1 H, br. s., Cys-αH) 3.06 (3 H, d, *J*=7.32 Hz, Arg-βH, Cys-βH) 2.98 (2 H, d, *J*=12.21 Hz, Arg-βH, Arg-γH) 2.85 (1 H, d, *J*=12.21 Hz, Arg-δH) 2.66 (1 H, dd, *J*=14.65, 9.77 Hz, Arg- δH) 1.58 - 1.70 (2 H, m, Leu-βH, Thr-βH) 1.40 - 1.54 (7 H, m, Leu-βH, Leu-γH, Arg-γH) 1.04 (3 H, d, *J*=7.32 Hz, Thr-γH) 0.81 - 0.90 (6 H, m, Leu-δH); Analytical HPLC (220 nm) 19.8 min; IR (neat) 3271, 1643, 1134 cm^{-1}; MS (ESI+) *m/z* (%) 813.1 ((M + H)$^+$, 100.0), 407.1 ((M + 2H)$^+$, 78.1); HRMS (ESI+) for $C_{28}H_{44}Br_2N_8O_8S$ (M + H)$^+$ calcd 811.1452, found 811.1443.

HPLC:

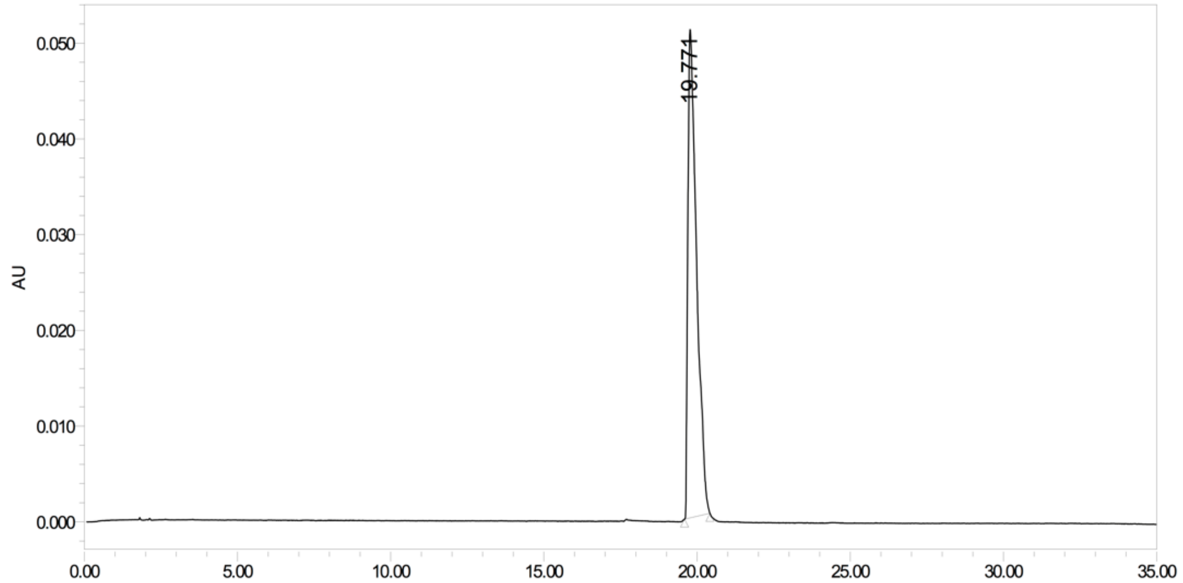

Mass Spectrum:

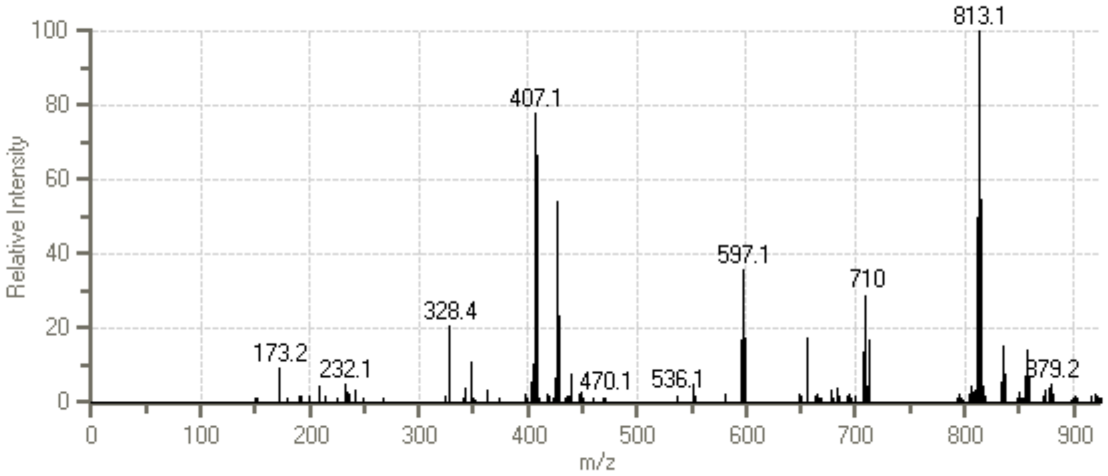

High resolution mass spectrum:

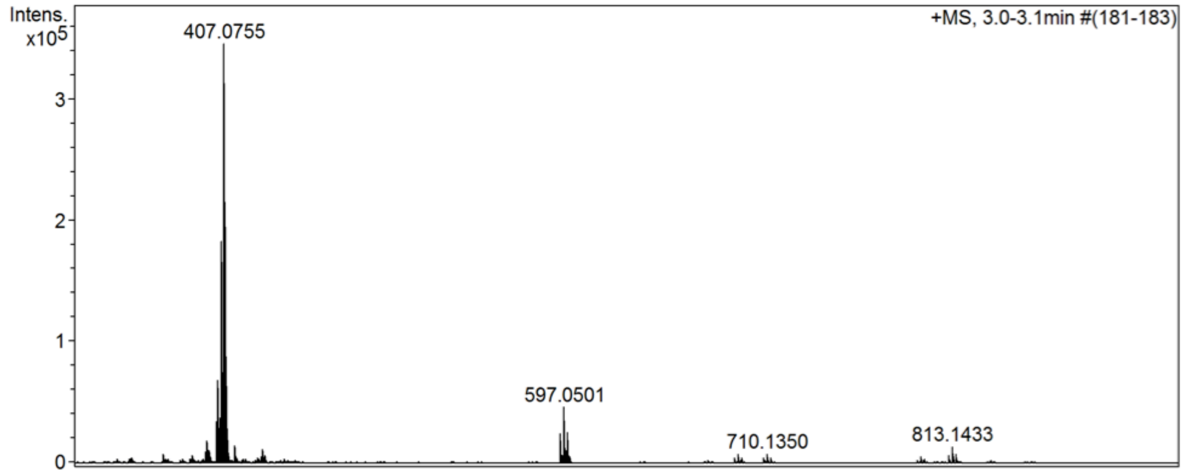

^{1}H NMR:

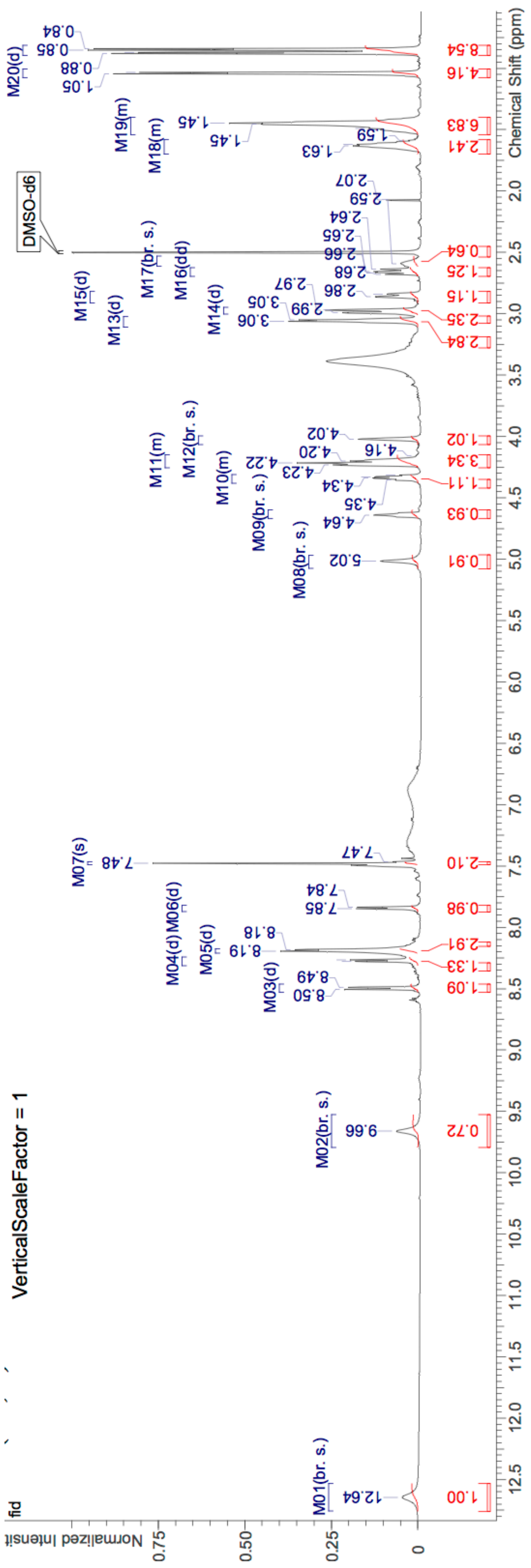
fid
VerticalScaleFactor = 1
DMSO-d6
Normalized Intensit
Chemical Shift (ppm)

CLR(4-Cl-F)T

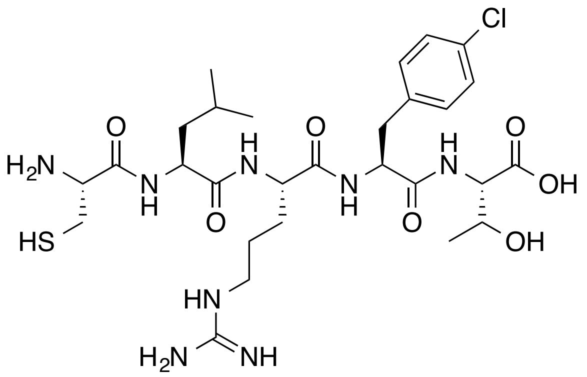

The peptide was synthesised using standard solid phase peptide synthesis techniques, with Fmoc-protected amino acids, yielding 19 mg (11%) of the product as a white solid. ^{1}H NMR (600 MHz, DMSO-d_6) δ ppm 8.51 (1 H, d, *J*=7.93 Hz, Leu-NH) 8.15 - 8.19 (2 H, m, Arg-NH and Thr-NH) 7.97 (1 H, d, *J*=7.93 Hz, Phe-NH) 7.50 - 7.58 (1 H, m, Arg-$NH_{side\text{-}chain}$) 7.23 - 7.31 (5 H, m, Phe-ArH) 4.99 (1 H, br. s., Thr-OH or Ser-OH) 4.68 (1 H, td, *J*=8.54, 4.27 Hz, Phe-αH) 4.31 - 4.36 (1 H, m, Leu-αH) 4.24 (1 H, q, *J*=7.43 Hz, Arg-αH) 4.18 - 4.22 (1 H, m) 4.17 (1 H, br. s., Thr-αH) 4.03 (1 H, t, *J*=5.04 Hz, Cys-αH) 3.33 (12 H, br. s., Solvent-H_2O) 3.03 - 3.08 (4 H, m, Phe-βH, Arg-δH) 2.99 (1 H, dd, *J*=14.34, 5.80 Hz, Cys-βH) 2.85 (1 H, dd, *J*=14.34, 4.58 Hz, Cys-βH) 2.79 (1 H, dd, *J*=14.04, 9.16 Hz, Phe-βH) 1.59 - 1.66 (3 H, m, Arg-βH and Leu-βH) 1.35 - 1.51 (7 H, m, Leu-γH and Arg-γH) 1.04 (4 H, d, *J*=6.41 Hz, Thr-βH and Thr-γH) 0.89 (3 H, d, *J*=6.71 Hz, Leu-γH) 0.85 (4 H, d, *J*=6.41 Hz, Leu-δH); Analytical HPLC (220 nm) 18.6 min; IR (neat) 3271, 1629, 1133 cm^{-1}; MS (ESI+) *m/z* (%) 673.3 ($(M + H)^+$, 99.9), 337.2 ($(M + 2H)^+$, 100.0)); HRMS (ESI+) for $C_{28}H_{45}ClN_8O_7S$ $(M + H)^+$ calcd 673.2887, found 673.2888.

HPLC:

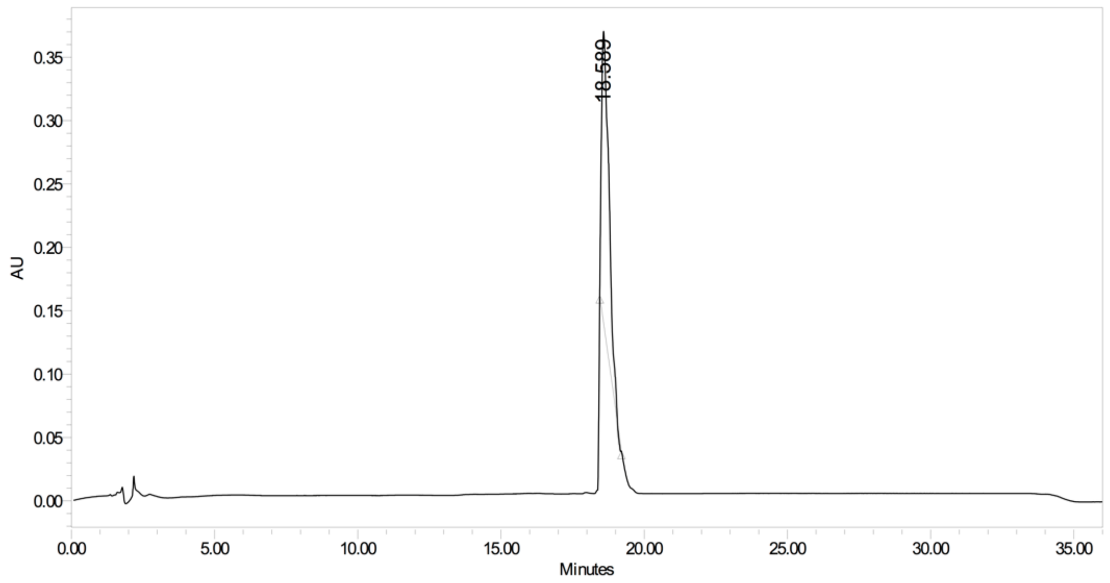

Mass Spectrum:

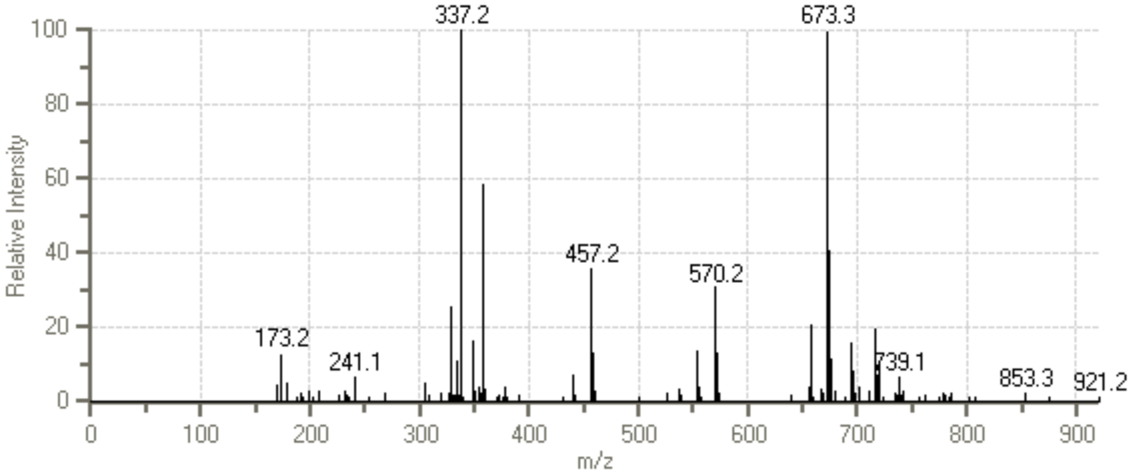

High resolution mass spectrum:

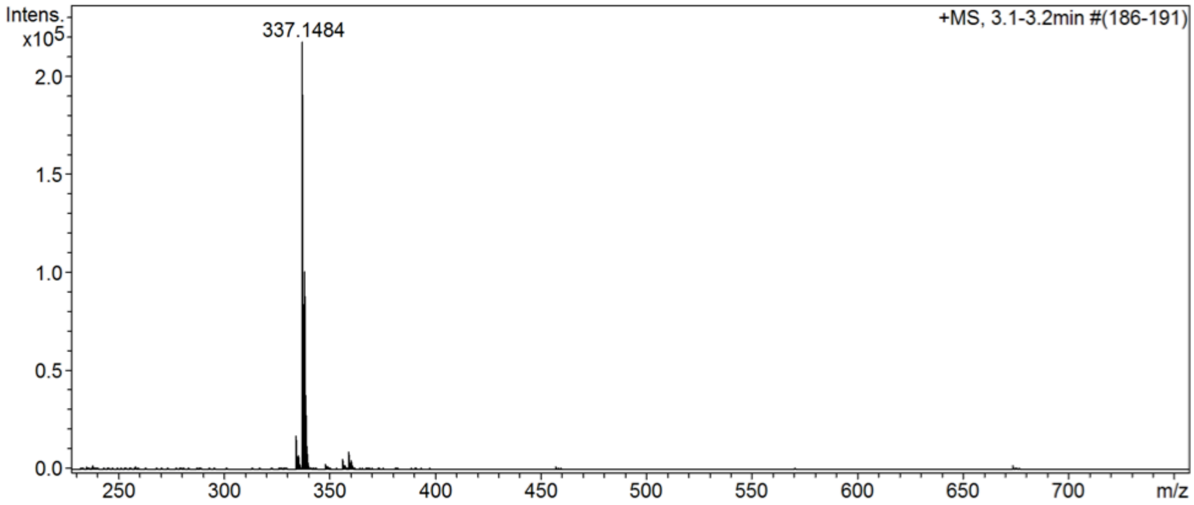

^{1}H NMR:

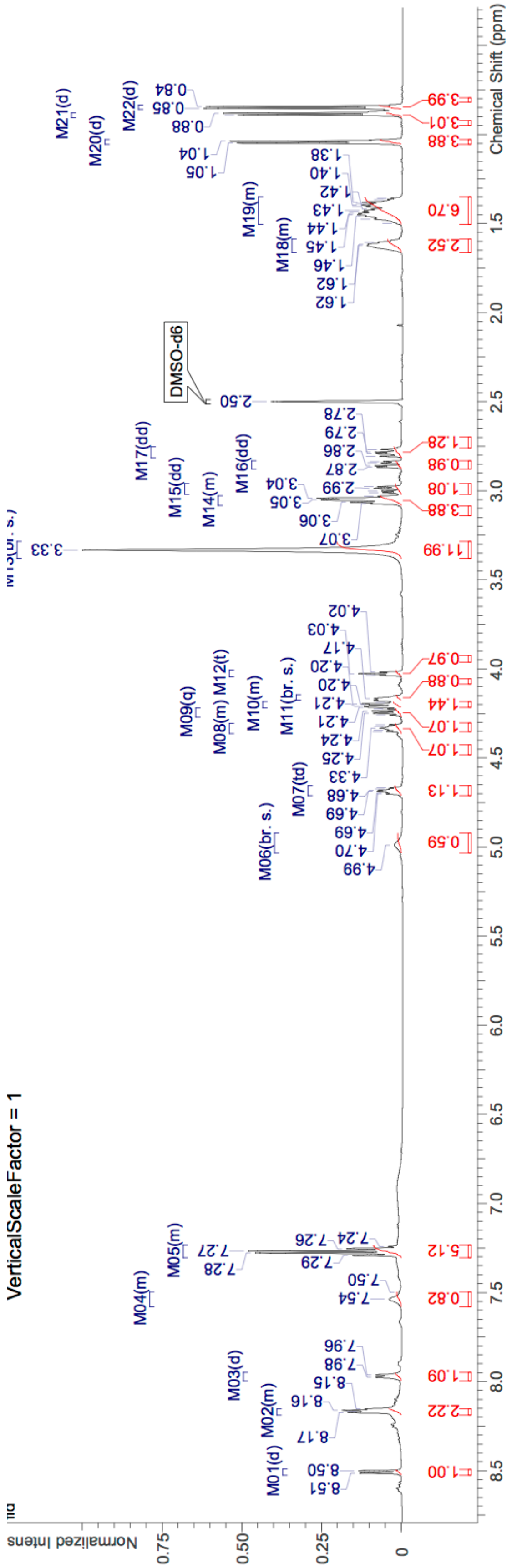
VerticalScaleFactor = 1
DMSO-d6
Normalized Intens
Chemical Shift (ppm)

CLRF(4-F)T

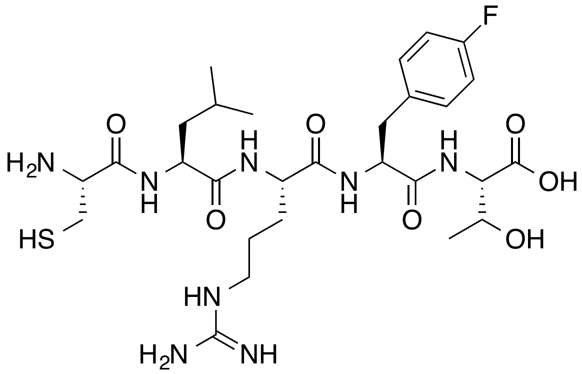

The peptide was synthesised using standard solid phase peptide synthesis techniques, with Fmoc-protected amino acids, yielding 48 mg (29%) of the product as a white solid. ^{1}H NMR (600 MHz, DMSO-d_6) δ ppm 12.63 (1 H, br. s., Thr-COOH) 8.51 (1 H, d, *J*=7.32 Hz, Leu-NH) 8.19 (2 H, br. s., Arg-NH) 8.15 (1 H, d, *J*=7.32 Hz, Thr-NH) 7.92 - 7.98 (1 H, m, Phe-NH) 7.50 (1 H, br. s., Arg-$NH_{side-chain}$) 7.25 - 7.32 (2 H, m, Phe-ArH) 7.02 (2 H, t, *J*=7.32 Hz, Phe-ArH) 4.99 (1 H, br. s., Cys-SH) 4.66 - 4.71 (1 H, m, Phe-αH) 4.30 - 4.35 (1 H, m, Leu-αH) 4.14 - 4.27 (3 H, m, Thr-αH, Arg-αH) 4.03 (1 H, br. s., Cys-αH) 3.36 (6 H, br. s., Solvent-H_2O) 2.96 - 3.10 (5 H, m, Phe-βH, Arg-δH, Thr-βH) 2.85 (1 H, d, *J*=12.21 Hz, Arg-βH) 2.78 (1 H, dd, *J*=14.65, 9.77 Hz, Arg-βH) 1.62 (2 H, d, *J*=4.88 Hz, Leu-βH, Thr-βH) 1.33 - 1.51 (6 H, m, Leu-βH, Leu-γH, Arg-γH) 1.05 (4 H, d, *J*=4.88 Hz, Thr-γH) 0.83 - 0.89 (6H, m, Leu-δH); Analytical HPLC (220 nm) 18.1 min; IR (neat) 3272, 1632, 1188 cm^{-1}; MS (ESI+) *m/z* (%) 657.3 ($(M + H)^+$, 51.3), 304.4 ($(M + 2H)^+$, 100.0)); HRMS (ESI+) for $C_{28}H_{45}FN_8O_7S$ $(M + H)^+$ calcd 657.3199, found 657.3171.

HPLC:

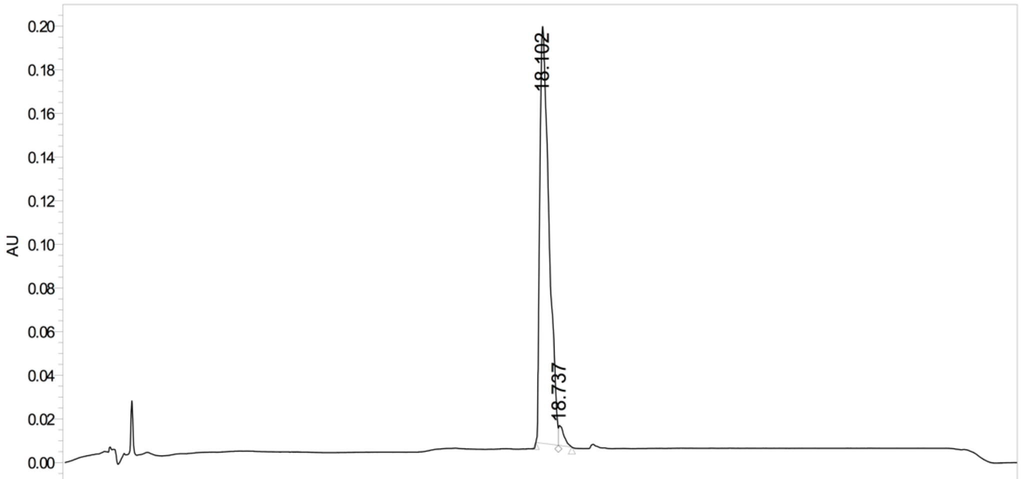

Mass Spectrum:

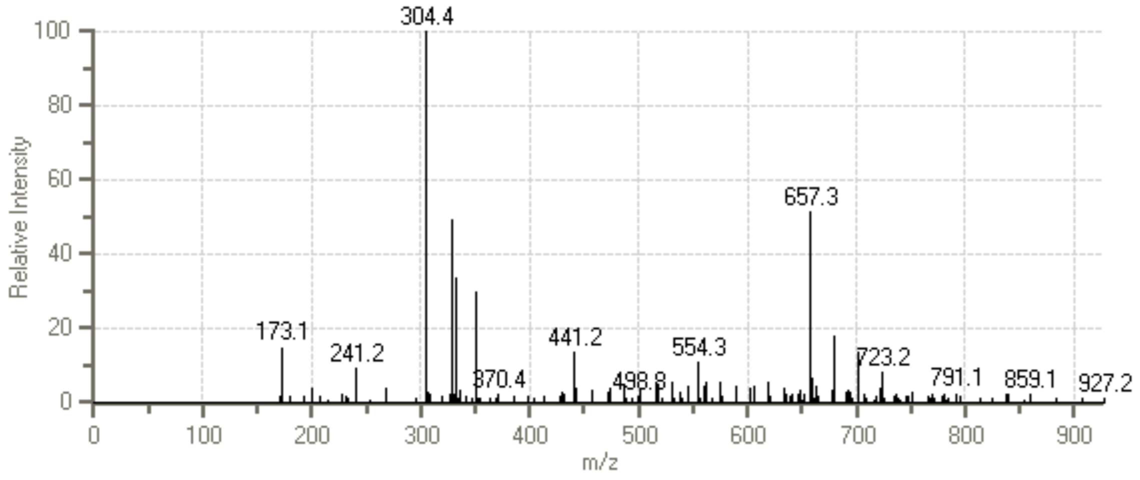

High resolution mass spectrum:

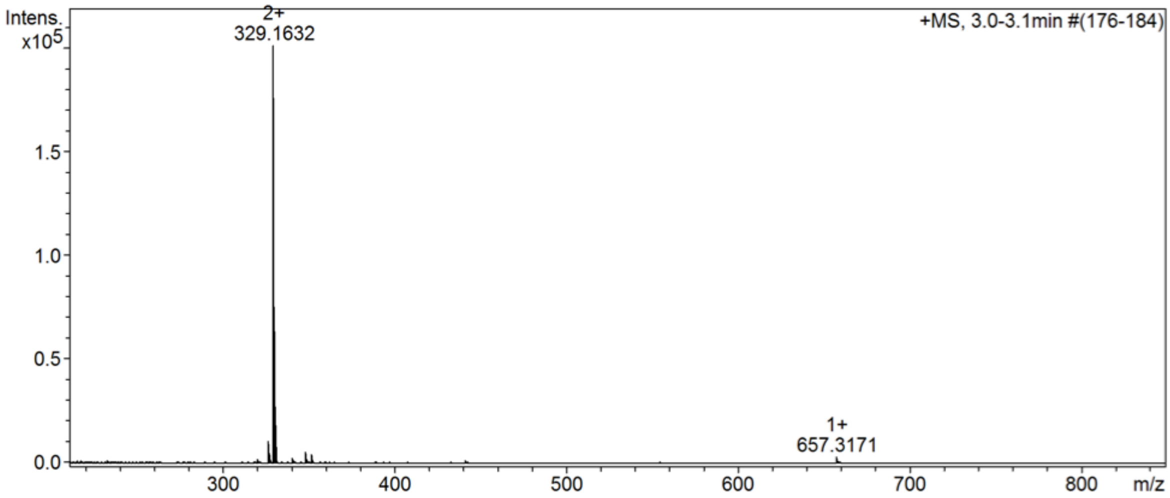

^{1}H NMR:

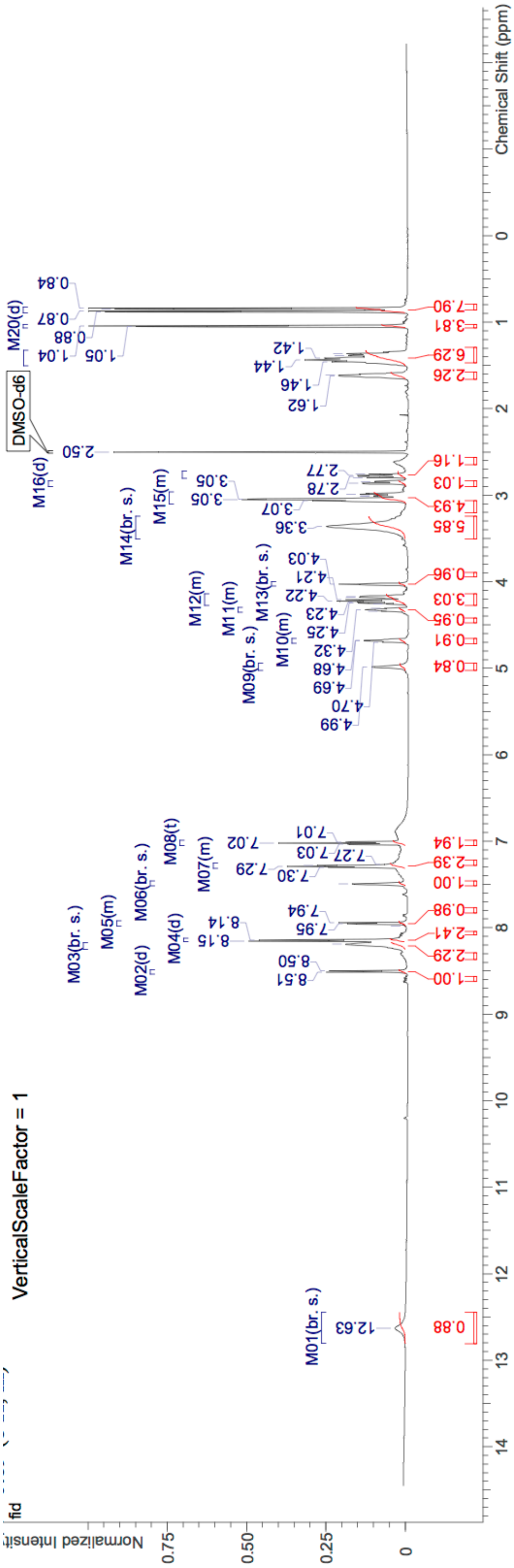
VerticalScaleFactor = 1
fid
Normalized Intensity
Chemical Shift (ppm)
DMSO-d6
2.50
M01(br. s.)
12.63
M02(d)
M03(br. s.)
M04(d)
M05(m)
M06(br. s.)
M07(m)
M08(t)
M09(br. s.)
M10(m)
M11(m)
M12(m)
M13(br. s.)
M14(br. s.)
M15(m)
M16(d)
M20(d)
8.51
8.50
8.15
8.14
7.95
7.94
7.30
7.29
7.27
7.03
7.02
7.01
4.99
4.70
4.69
4.68
4.32
4.25
4.23
4.22
4.21
4.03
3.36
3.07
3.05
3.05
2.78
2.77
1.62
1.46
1.44
1.42
1.05
1.04
0.88
0.87
0.84
1.00
2.29
2.41
0.98
1.00
2.39
1.94
0.84
0.91
0.95
3.03
0.96
5.85
4.93
1.03
1.16
2.26
6.29
3.81
7.90
0.88

CLR (3-NO_2-Y)T

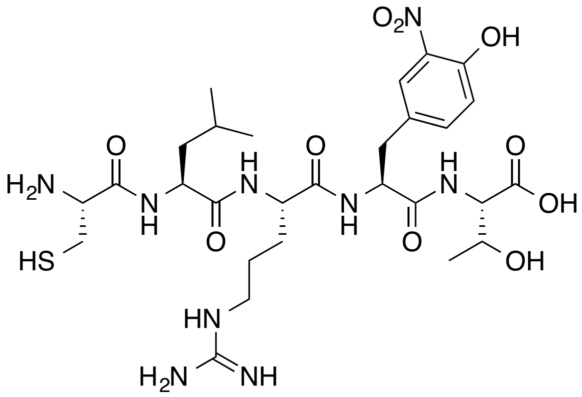

The peptide was synthesised using standard solid phase peptide synthesis techniques, with Fmoc-protected amino acids, yielding 66 mg (38%) of the product as an orange solid. ^{1}H NMR (600 MHz, DMSO-d_6) δ ppm 10.77 (1 H, br. s., Thr-COOH) 9.23 - 9.30 (1 H, m, Tyr-OH) 8.49 (1 H, d, *J*=7.32 Hz, Leu-NH) 8.17 - 8.25 (4 H, m, Thr-NH) 8.15 (1 H, d, *J*=7.32 Hz, Tyr-NH) 7.93 (1 H, d, *J*=7.32 Hz, Arg-NH) 7.84 (1 H, s, Tyr-ArH) 7.52 (1 H, br. s., Arg-$NH_{side-chain}$) 7.47 (1 H, d, *J*=9.77 Hz, Tyr-ArH) 7.00 (1 H, d, *J*=7.32 Hz, Tyr-ArH) 5.01 (1 H, br. s., Cys-SH) 4.65 - 4.73 (1 H, m, Arg-αH) 4.28 - 4.36 (1 H, m, Leu-αH) 4.16 - 4.26 (3 H, m, Thr-αH, Tyr-αH) 4.03 (1 H, br. s., Cys-αH) 2.94 - 3.10 (5 H, m, Arg-βH, Leu-βH, Tyr-βH) 2.82 - 2.89 (1 H, m Cys-βH) 2.75 (1 H, dd, *J*=14.65, 9.77 Hz, Arg-γH) 2.56 - 2.62 (1 H, m, Cys-βH) 1.61 (2 H, br. s., Leu-δH, Leu-γH) 1.34 - 1.52 (6 H, m, Arg-δH, Leu-γH, Thr-βH) 1.04 (3 H, d, *J*=7.32 Hz, Thr-γH) 0.82 - 0.88 (6 H, m, Leu-δH); Analytical HPLC (220 nm) 17.6 min; IR (neat) 3284, 1627, 1182 cm^{-1}; MS (ESI+) *m/z* (%) 700.2 ((M + H)$^+$, 91.0), 350.9 ((M + 2H)$^+$, 100.0)); HRMS (ESI+) for $C_{28}H_{45}N_9O_{10}S$ (M + H)$^+$ calcd 700.3083, found 700.3072.

HPLC:

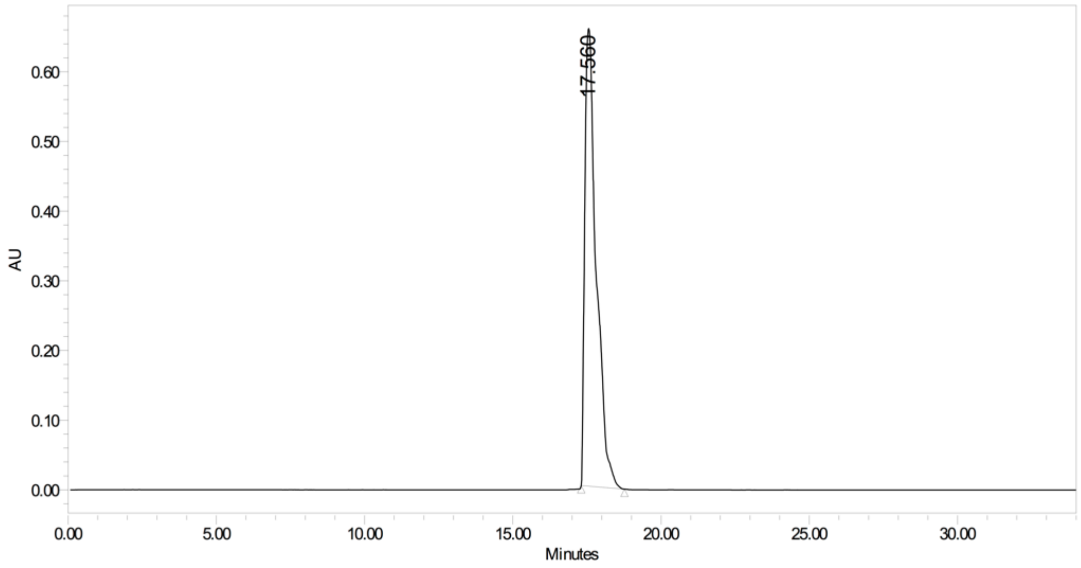

Mass Spectrum:

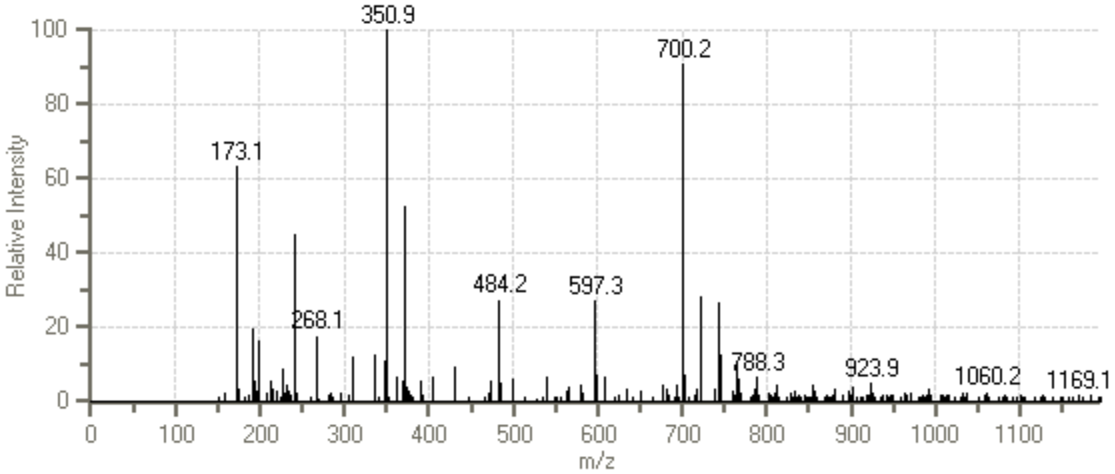

High resolution mass spectrum:

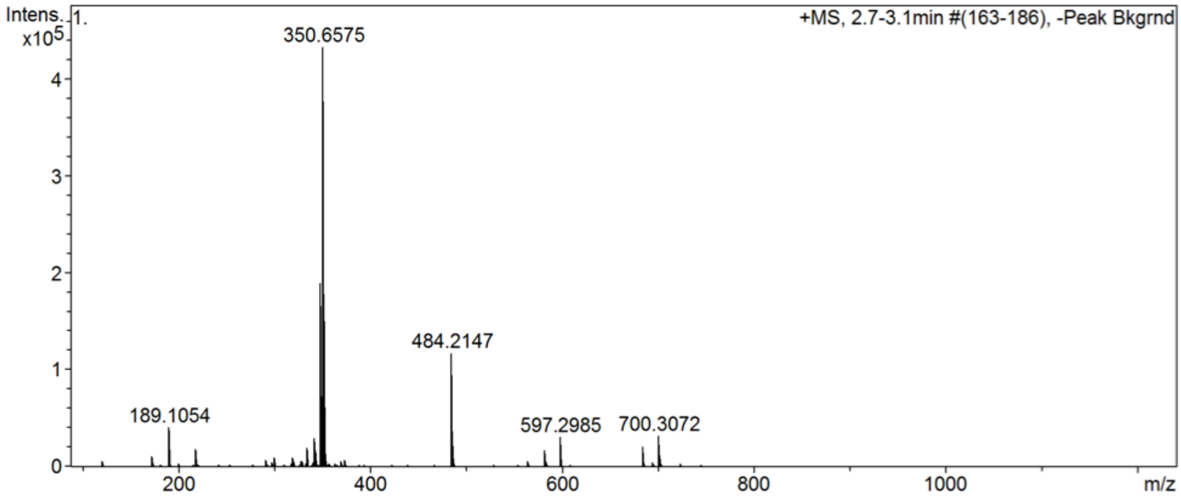

^{1}H NMR:

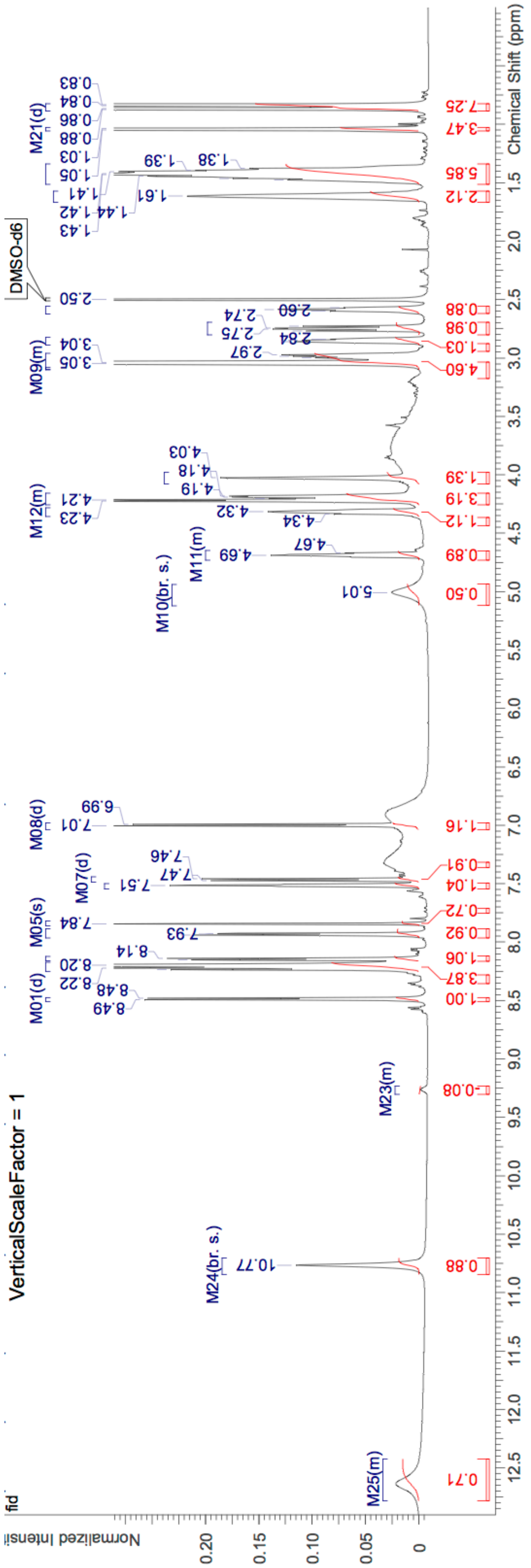
VerticalScaleFactor = 1
fid
DMSO-d6
M01(d)
M05(s)
M07(d)
M08(d)
M09(m)
M10(br. s.)
M11(m)
M12(m)
M21(d)
M23(m)
M24(br. s.)
M25(m)
Normalized Intensi
Chemical Shift (ppm)

Fluorescein-tagged CLR(4-Cl-F)T

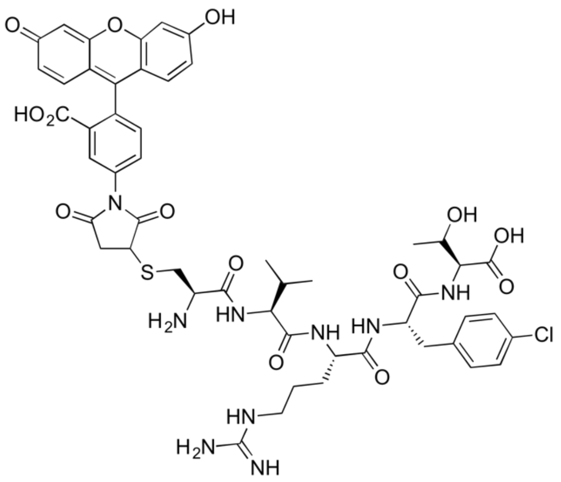

CLR(4-Cl-F)T was synthesized as detailed above. The peptide (4.2 mg, 5.8 μM) was dissolved in a 1:1 solution of DMF/H_2O (1 mL). Fluorescein-5-maleimide (4.2 mg, 11.6 μM) was added, and the solution stirred overnight, with the flask protected from light. The solvent was remover in vacuo, and the product purified by column chromatography, using a DCM/MeOH (85:15) to remove excess fluorescein-5-maleimid, followed by 100% methanol to elute the product. The solvent was removed in vacuo to yield 2.6 mgs of the product (41% yield) as a yellow solid. MS (ESI+) *m/z* (%) 550.7 ($(M + 2H)^+$, 35.0), 367.7 ($(M + 3H)^+$, 100.0)).

Mass Spectrum

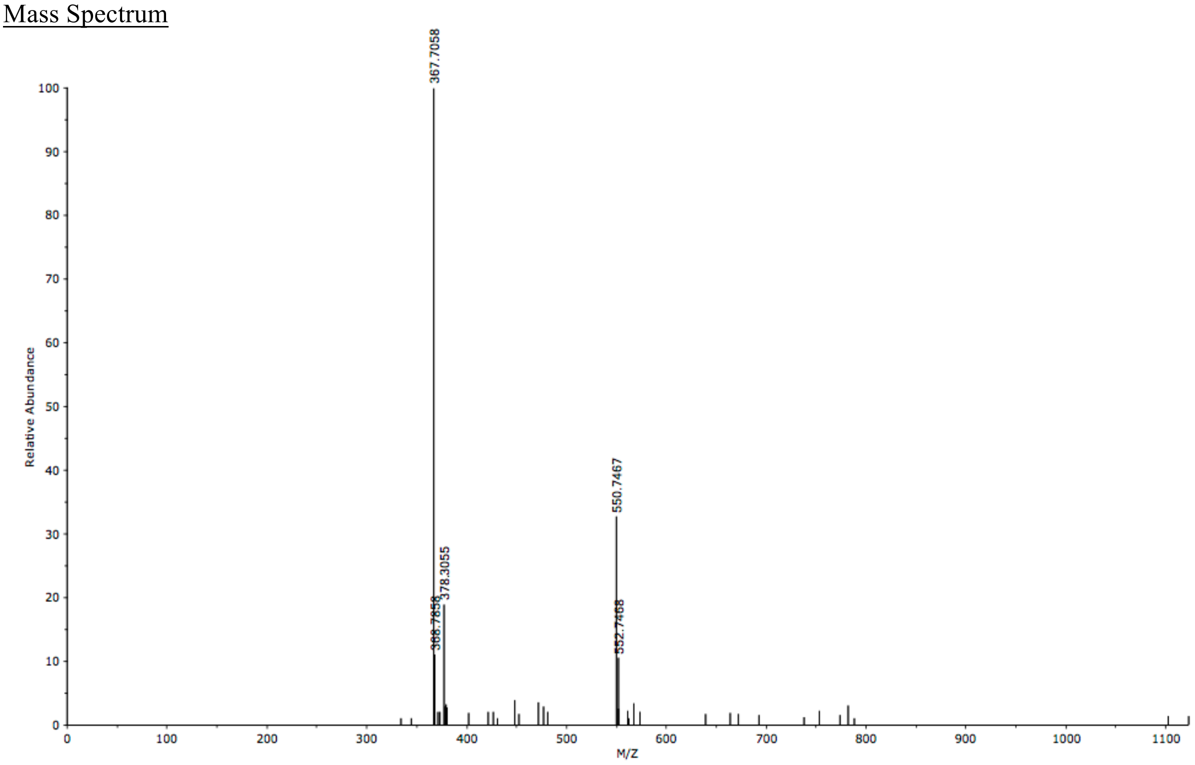

Fluorescein-tagged (4-Cl-F)CRTL

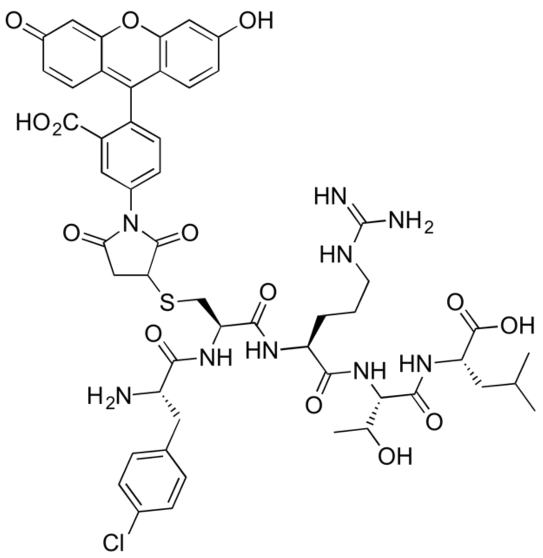

Fluorescein-tagged (4-Cl-F)CRTL was prepared as detailed above for fluorescein-tagged CLR(4-Cl-F)T. 2.2 mgs of the product (35% yield) was isolated as a yellow solid. MS (ESI+) *m/z* (%) 551.0 ((M + 2H)$^+$, 71.0), 367.6 ((M + 3H)$^+$, 100.0)).

Mass Spectrum

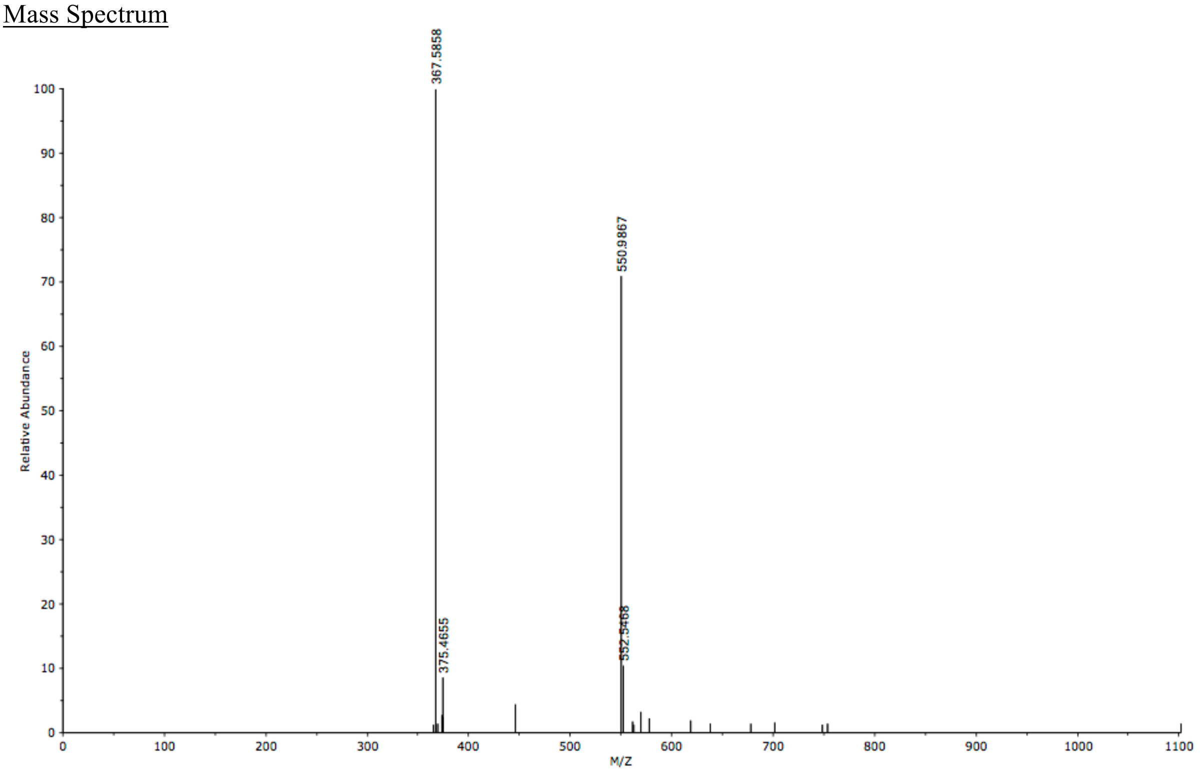

Supplement: Supplementary file 1 — Supplementary Information [file 41598_2017_3253_MOESM1_ESM.pdf]
